# Highly Selective Ortho‐Directed Dicarboxylation of Cyclopentadiene by Methylcarbonates and CO_2_ or COS – First Insight into Co‐ordination Chemistry of New Ambident Ligands

**DOI:** 10.1002/chem.202100300

**Published:** 2021-05-13

**Authors:** Tobias Vollgraff, Jörg Sundermeyer

**Affiliations:** ^1^ Fachbereich Chemie and Wissenschaftliches Zentrum für Materialwissenschaften (WZMW) Philipps-Universität Marburg Hans-Meerwein-Straße 4 35043 Marburg Germany

**Keywords:** Ambident Ligands, Carbon dioxide fixation, Carboxylate ligands, Cyclopentadienyl ligands, Ionic liquids

## Abstract

This research presents the highly regioselective syntheses of 1,2‐dicarboxylated cyclopentadienide salts [Cat]_2_[C_5_H_3_(CO_2_)_2_H] by reaction of a variety of organic cation methylcarbonate salts [Cat]OCO_2_Me (Cat=NR_4_^+^, PR_4_^+^, Im^+^) with cyclopentadiene (CpH) or by simply reacting organic cation cyclopentadienides Cat[Cp] (Cat=NR_4_^+^, PR_4_^+^, Im^+^) with CO_2_. One characteristic feature of these dianionic ligands is the acidic proton delocalized in an intramolecular hydrogen bridge (IHB) between the two carboxyl groups, as studied by ^1^H NMR spectroscopy and XRD analyses. The reaction cannot be stopped after the first carboxylation. Therefore, we propose a Kolbe‐Schmitt phenol‐carboxylation related mechanism where the acidic proton of the monocarboxylic acid intermediate plays an *ortho‐*directing and CO_2_ activating role for the second kinetically accelerated CO_2_ addition step exclusively in *ortho* position. The same and related thiocarboxylates [Cat]_2_[C_5_H_3_(COS)_2_H] are obtained by reaction of COS with Cat[Cp] (Cat=NR_4_^+^, PR_4_^+^, Im^+^). A preliminary study on [Cat]_2_[C_5_H_3_(CO_2_)_2_H] reveals, that its soft and hard coordination sites can selectively be addressed by soft Lewis acids (Mo^0^, Ru^2+^) and hard Lewis acids (Al^3+^, La^3+^).

## Graphical abstract

**A highly regioselective synthesis of 1,2‐dicarboxylated and ‐dithiocarboxylated cyclopentadienide salts [Cat]_2_[C_5_H_3_(CO_2_)_2_H]** by reaction of organic cation methylcarbonate salts Cat[OCO_2_Me] (Cat=DMP^+^, NMe_4_^+^, EMMIm^+^) with cyclopentadiene or by reaction of CO_2_/COS with Cat[Cp] (Cat=DMP^+^, NMe_4_^+^, PPh_4_^+^) was developed. First insight into their coordination chemistry reveals ambident ligand behaviour following Pearsons acid‐base concept. In some reactions with metal Lewis acids, a slow decarboxylation of the [C_5_H_3_(CO_2_)_2_H] ligand was observed. The mechanism is discussed.

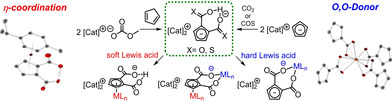

## Introduction

### Methylcarbonate ionic liquids and NHC−CO_2_ or NHO−CO_2_ adducts as carboxylation reagents

The use of dimethyl carbonate as a mild and non‐poisonous methylation agent for nucleophilic cation precursors is by far the greenest method to synthesize methyl‐onium cation methylcarbonate ionic liquids (ILs) and related organic methylcarbonates in a sustainable metal‐ and halide‐free fashion.[^1^, ^2^, ^3^, ^4^] The only limitation is the need for thermal stability of the resulting IL cations towards the basic and nucleophilic, potentially weakly solvated methyl carbonate anions and their cation carboxylation potential.[^4^, ^5^] The borderline cases with respect to cation stability are 1,3‐ di‐ and 1,2,3‐trialkylimidazolium methylcarbonates. They can be handled only in methanol solution where the basic methyl carbonate anions are stabilized by solvation via hydrogen bridges, otherwise these salts tend to decompose to form imidazolium‐carboxylate zwitterions NHC−CO_2_ and NHO−CO_2_ derived from n‐heterocyclic carbenes (NHC) and olefins (NHO).[^2^, ^3^, ^6^] According to literature reports, NHC−CO_2_ can be prepared selectively by feeding gaseous CO_2_ into a solution of the corresponding carbene,^[7]^ directly from 1‐methylimidazole and dimethyl carbonate,[^2^, ^5^] by continuous flow at 200 °C over an Al_2_O_3_ catalyst,^[8]^ and even at ambient temperature by introducing CO_2_ into imidazolium acetate ILs.^[9]^ Specifically, the first and last approaches allow extension to imidazolium‐2‐thio‐ and dithiocarboxylates.^[10]^ These compounds can be employed as masked proto‐carbenes in the formation of transition‐metal NHC complexes,^[11]^ act as CO_2_ transfer reagents and organocatalysts,[^12^, ^13^] and can be used as masked carbenes for the synthesis of ILs by reaction with sufficiently acidic reagents.[^2^, ^5^, ^14^] Beside this, the selective synthesis of imidazolium‐2‐methylenecarboxylates (NHO−CO_2_) can be achieved from the in situ generated NHOs and CO_2_ in a quite comparable way to the NHC−CO_2_ and can be also extended to COS and CS_2_ adducts.^[15]^ The NHOs, which are generated in situ have also been employed as highly basic starting materials for the synthesis of ionic liquids^[16]^ and as polymerization catalysts for propylene oxide,^[17]^ methyl methacrylate^[18]^ and lactones.^[19]^ They are also versatile organocatalysts for transesterification^[20]^ and base‐promoted alkylation reactions.^[21]^ Furthermore, they are interesting ligands in main‐group and transition‐metal complexes.^[22]^ The potential of NHC−CO_2_ adducts as transcarboxylation agent was demonstrated by louie and coworkers.^[23]^ Under action of MBPh_4_ (M=Na oder Li) as Lewis acid, acetophenone was converted to sodium or lithium benzoylacetates, respectively.

Biscarboxylation of acetonitrile to dianionic cyanomalonate was observed with NHC−CO_2_ type zwitterions 1,3‐di‐tert‐butylimidazolium‐2‐carboxylate^[23]^ and NHO−CO_2_ type zwitterions 1‐ethyl‐3‐methylimidazolium‐2‐methylenecarboxylate.^[6]^ Based on these promising results there is an enhanced focus on developing more C−C coupling reactions based on NHO−CO_2_ and NHC−CO_2_ adducts with CH acidic reaction partners, aiming for developing a protocol with only an organo catalytic amount of NHC− and NHO−CO_2_ in future.[^12^, ^15^, ^24^]

Besides self‐carboxylation of the imidazolium cation in imidazolium methylcarboxylates, the easily accessible and diverse methylcarbonate ILs with different organic cations were to the best our knowledge not used in C‐carboxylations of other CH‐acids until now. This aspect ahead, we present a method to synthesize 1,2‐dicarboxylated cyclopentadienide salts [Cat]_2_[C_5_H_3_(CO_2_)_2_H] selectively by reaction of methylcarbonate salts with CpH.

### Cyclopentadiene carboxylates

The carboxylation of CpH (pK_a_=∼16)[^25^, ^26^] was first mentioned by Thiele in 1900.[^25^, ^27^] He described the preparation of dicyclopentadiene dicarboxylic acid by reaction of KCp with CO_2_ accompanied by spontaneous DA dimerization of C_5_H_5_−COOH yielding mixtures of isomers during the acidic work‐up. Later on, it was observed that the corresponding sodium salt (NaCp) could be used as well,^[28]^ and the procedure was optimized yielding one major isomer of DA dimer.^[29]^

**Scheme 1:**
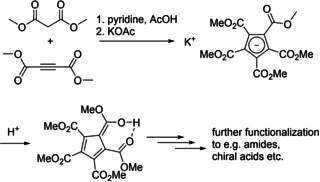
Synthesis of PCCP potassium salt from dimethyl malonate and dimethyl acetylenedicarboxylate.[^38^, ^39^]

Carboxylation of CpH and multiple carboxylated Cp ligands are of interest with respect to the design of water‐soluble metal complexes for biological applications.^[30]^ Targeting this aspect monocarboxylated cyclopentadiene species were obtained in form of their esters by reaction of NaCp with dimethyl carbonate^[31]^ or with methyl bromoformate.^[32]^ LiCp can be reacted directly with CO_2_ ^[33]^ yielding the *mono* carboxylic acid after acidic work‐up. Alternatively, the carboxyl group can be introduced on already synthesized and ring lithiated half‐sandwich complex CpMn(CO)_3_.^[34]^ 1,1′‐Ferrocenedicarboxylic acid ethylester, fc(COOEt)_2_, was obtained in one pot synthesis by ethoxycarbonylation of CpH/NaOEt with ethylcarbonate in the presence of FeCl_2_.^[35]^ The research towards higher functionalized Cp rings focuses mainly on pentacarboxylated compounds.[^36^, ^37^, ^38^, ^39^] Due to the incorporation of electron‐withdrawing ring substituents they approach the acidity of HCl(aq).^[37]^ These pentacarboxycyclopentadienes (PCCPs) have been used as cyclopentadienyl‐type ligands[^40^, ^41^, ^42^] in coordination chemistry. They served as a novel class of enantioselective Brønsted acid catalysts^[43]^ and Lewis acids.^[44]^ However, to the best of our knowledge, their potentially chelating carboxylate groups have never been used to bind a metal, e. g. next to a different (hetero)metal bound to the η^5^‐cyclopentadienyl carbanion. A typical synthesis of PCCPs and their deprotection via ester hydrolysis is shown in Scheme 1.^[45]^

**Scheme 2:**
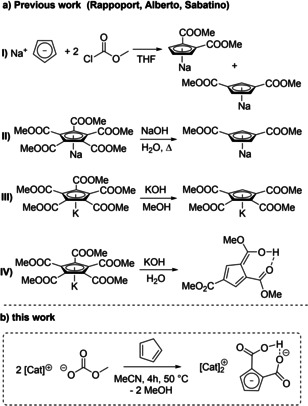
Previously reported multistep‐procedure to di‐, tri‐, and tetrasubstituted sodium and potassium cyclopentadienide carboxylate methylesters and our one‐step synthesis yielding [Cat]_2_[C_5_H_3_(CO_2_)_2_H] salts.[^30^, ^46^, ^48^, ^49^]

There are only limited examples of selective dicarboxylations of CpH known.[^30^, ^46^, ^47^, ^48^, ^49^] Alberto and coworkers observed the formation of 1,2‐ and 1,3‐dicarboxylated as well as 1,2,4‐tricarboxylated Cp salt mixtures in moderate yield by reacting NaCp with methyl chloroformate.^[30]^ The lack of regioselectivity can be overcome by an environmentally non‐preferable two‐step cation exchange with Tl_2_SO_4_ before continuing with further functionalization steps. These authors presented Re‐ and Tc‐complexes with this ester masked [1,2‐C_5_H_3_(CO_2_Me)_2_] ligand and its subsequent hydrolysis by hydroxide to yield the uncharged [(η^5^‐C_5_H_3_(1,2‐COOH)_2_)Re(CO)_3_] and [(η^5^‐C_5_H_3_(1,2‐COOH)_2_)^99m^Tc(CO)_3_] complexes planned to be used as molecular imaging agents.^[30]^ (Scheme 2).

In the following section, we combine both introduced aspects and describe the first example of a highly regioselective C−C bond‐forming carboxylation reaction of a prominent CH‐acid with above mentioned methylcarbonate salts Cat[OCO_2_Me]. The products [Cat]_2_[C_5_H_3_(CO_2_)_2_H] (**1 a**–**d**) are obtained in a facile one‐step synthesis. Their application as an ambident ligand with two coordination sites, the soft carbanionic backbone and the hard “acac‐related” or “carboxylate‐type” bidentate *O,O*‐donor character is exemplarily demonstrated.

## Results and Discussion

### Synthesis of the title compounds

**Scheme 3:**
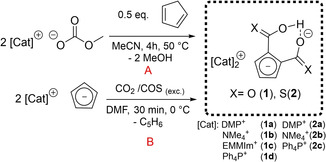
Synthetic approaches to [Cat]_2_[C_5_H_3_(COX)_2_H] salts **1 a**–**d** and **2 a**–**c** by two possible strategies (DMP=N,N’‐dimethylpyrrolidinium; EMMIm=1‐ethyl‐2,3‐dimethylimidazolium).

Our targets [Cat]_2_[C_5_H_3_(CO_2_)_2_H] (**1 a**–**d**) are accessible by the reaction of two equivalents of the corresponding methylcarbonate salt Cat[OCO_2_Me] (Cat=e. g. *N,N‐*dimethylpyrrolidinium DMP^+^, 1‐ethyl, 2,3‐dimethylimidazolium EMMIm^+^) and CpH in MeCN at 50 °C for 2 h. The desired compound precipitates from reaction mixture in high yield. Another synthetic approach is the reaction of the corresponding salt Cat[Cp] (Cat=e. g. DMP^+^, PPh_4_^+^) synthesized according to a procedure of Harder and co‐workers^[50]^ with CO_2_ gas by bubbling the gas through a DMF solution of the Cp salt at 0 °C. A change of solvent to MeCN leads to precipitation of the corresponding [Cat]_2_[C_5_H_3_(CO_2_)_2_H] salts. The yield can be increased by cooling the saturated MeCN solution down to −20 °C overnight. Scheme 3 displays the two independent procedures A and B and the investigated scope of cations. Following both strategies there are almost no restrictions concerning the variety of accessible cations. In this report we focus on further transformations of the salt [DMP]_2_[C_5_H_3_(CO_2_)_2_H] (**1 a**) as it shows sufficient solubility, good crystallization properties due to cation symmetry and cation inertness with respect to cation carboxylation in contrast to imidazolium salts. The preparation and characterization of related compounds **1 b**–**1 d** and **2 b**–**2 c** are described in the Supporting Information.

**Figure 1:**
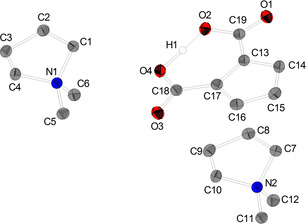
Molecular structure of [DMP]_2_[C_5_H_3_(CO_2_)_2_H] (**1 a**). Hydrogen atoms, solvent molecules and disordered atoms are neglected for clarity. Ellipsoids are shown at 50 % level. Selected bond lengths (Å) and angles (°) for a representative anion of **1 a**: O4−H1 1.2017(238), O2−H1 1.2308(241), O1−C19 1.247(2), O2−C19 1.309(2), O3−C18 1.244 (2), O4−C18 1.314(2), C13−C17 1.447(2), C14−C15 1.399(2), C15−C16 1.398(2), C13−C14 1.408(2), C16−C17 1.410(2), C13−C19 1.469(2), C17−C18 1.468(2), O3−C18−O4 119.95(13), O3−C18−C17 121.17(14), O4−C18−C17 118.88(13), O1−C19−O2 120.26(13), O1−C19−C13 121.23(13), O2−C19−C13 118.50(13).

Single crystals suitable for XRD analysis of [DMP]_2_[C_5_H_3_(CO_2_)_2_H] (**1 a**) were obtained by vapour diffusion of diethyl ether into a saturated solution of the target compound in MeCN. **1 a** crystallizes in the triclinic space group *P* $\bar{1}$ with Z=2 (Figure 1). Crystals isolated from mother liquor crystallize in the monoclinic space group *P*2_1_/*c* with Z=2 (see Supporting Information).

**Scheme 4:**
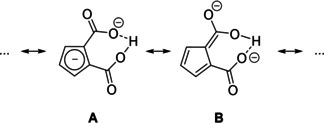
Resonance forms A and B of [Cat]_2_[C_5_H_3_(CO_2_)_2_H] salts.

Both carboxylic groups are almost in plane with the Cp ring according to the torsion angles (C18−C17−C13−C19: 0.7°; O4−C18−C17−C16: 1.0°). The C13−C19‐bond and C17−C18‐bond can be considered as single bonds (1.468(2); 1.469(3) Å), respectively. In contrast to reports describing the bonding situation of some disubstituted Cp carboxylic esters in solid state as enol‐type structure,[^46^, ^47^] in our examples, the molecular structure is best described as disubstituted cyclopentadienide anion (form A) with only little perturbation of aromaticity of the C_5_ ring. However, the bond C13−C17 (1.447(2) Å) connecting both carboxylic functions, which is pronounced longer than all other ring C−C distances C14−C15 1.399(2), C15−C16 1.398(2), C13−C14 1.408(2) and C16−C17 1.410(2) Å indicates some contribution of fulvalene enolate form B (Scheme 4).

The O1−C19 (1.247(2) Å) and the O3−C18 (1.244(2) Å) distances are best described as double bonds while O2−C19 (1.309(2) Å) and the O4−C18 (1.314(2) Å) are essentially single bonds with some double bond character. We expect the acidic proton position to be attached asymmetrically in an IHB closer to O4, since O4−C18 is slightly elongated compared to O2−C19 which is in agreement with the by the differential map determined position of the IHB hydrogen atom (O4−H1 1.202(2) Å and O2−H1 1.231(2) Å).

It was also possible to synthesize analogue dithiocarboxylated salts **2 a**–**c** by reacting Cat[Cp] with COS. Pure [DMP]_2_[C_5_H_3_(COS)_2_H] (**2 a**) and the related species **2 b**–**c** according to Scheme 3 were isolated in good yields after recrystallization of the precipitate from a MeCN/THF mixture.

**Figure 2:**
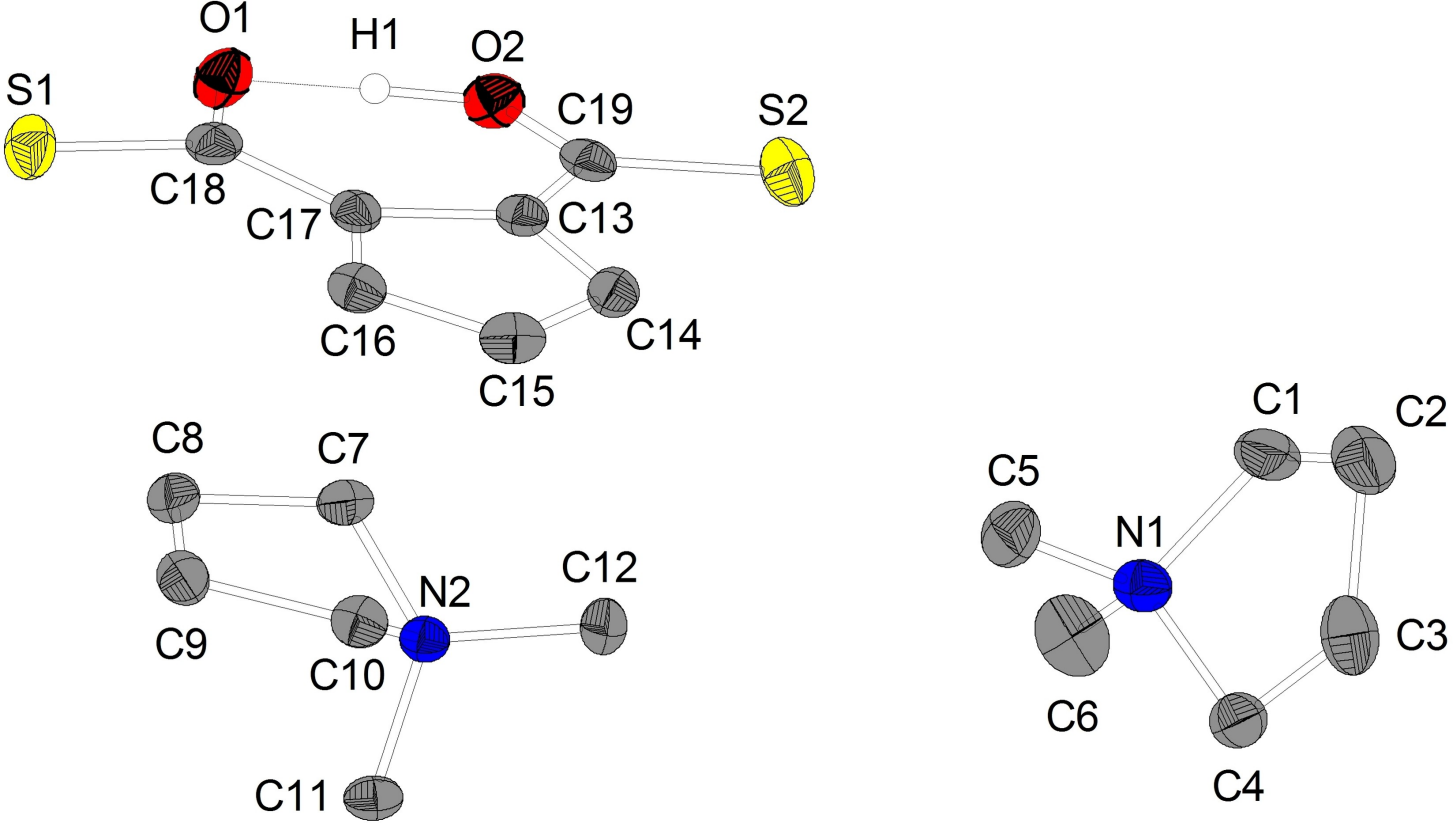
Molecular structure of [DMP]_2_[C_5_H_3_(COS)_2_H] (**2 a**). Hydrogen atoms and disordered atoms are neglected for clarity. Ellipsoids are shown at 50 % level. Selected bond lengths (Å) and angles (°) for a representative anion of **2 a**: O1−H1 1.3713(262), O2−H1 1.2017(238), S1−C18 1.716(2), O1−C18 1.293(2), O1−H1 1.37(3), S2−C19 1.698(2), O2−C19 1.299(2), O2−H1 1.05(3), C19−C13 1.440(3), C18−C17 1.438(3), C18−O1‐H1 111.6(12), C19−O2‐H1 111.5(16), O2−C19−C13 119.78(17), O2−C19−S2 117.55(14), C13−C19−S2 122.66(15), O1−C18−C17 119.90(16), O1−C18−S1 118.12(14), C17−C18−S1 121.98(14).

**Scheme 5:**
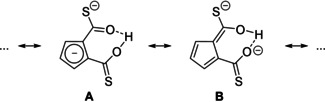
Two resonance forms of [Cat]_2_[C_5_H_3_(COX)_2_H] salts **2 a**–**c**.

Single crystals of [DMP]_2_[C_5_H_3_(COS)_2_H] (**2**) suitable for XRD analysis were obtained by vapour diffusion of diethyl ether into a saturated solution in MeCN. **2 a** crystallizes in the monoclinic space group *P*2_1_/*c* with Z=8 (Figure 2). Compared to the molecular structure of **1 a**, both thiocarboxyl groups are a bit more distorted from the Cp ring plane (C18−C17−C13−C19 3.6°; O1−C18−C17−C13 2.4°). Due to the less pronounced π‐donor character of S^2−^ compared to O^2−^, both exocyclic distances C13−C19 (1.441(3) Å) and C17−C18 (1.438(3) Å) are slightly shorter compared to the corresponding bonds of **1 a**, the fulvalene enolate form B tends to contribute slightly more to the ground state than in **1 a** (Scheme 5). This behaviour is also reflected in the lower π‐bond order of S1‐C18 1.716(2) and S2−C19 1.698(2) Å compared to C−O π‐bond order in corresponding **1 a**. Typical C−S bond distances with a bond order close to 2 fall into the region of about 1.6 Å.^[51]^

The less pronounced π‐bond order of C−S *vs*. C−O bonds in thiocarboxylates are in accord with orbital overlap arguments and have extensively been investigated by computational studies of thio esters with different substituents.^[52]^

**Figure 3:**
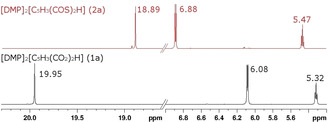
Comparison of the ^1^H NMR spectra of compounds **1 a** and **2 a** (DMSO‐d_6_, 300 MHz, 298 K).

The differences at electronic level can be confirmed by the comparison of the ^1^H NMR spectra of [DMP]_2_[C_5_H_3_(CO_2_)_2_H] (**1 a**) and [DMP]_2_[C_5_H_3_(COS)_2_H] (**2 a**) (Figure 3).

The protons in *ortho‐*position from the thiocarboxyl groups of **2 a** are more deshielded (6.88 ppm) compared to the corresponding protons of **1 a** (6.08 ppm). The proton of the H‐bridge is shifted towards high field (18.89 ppm).

As already mentioned, analogues salts of type [Cat]_2_[C_5_H_3_(CS_2_)_2_H] were not formed under identical conditions with CS_2_ replacing COS. Instead, we isolated only mono dithiocarboxylated species Cat[C_5_H_4_CS_2_H] and [Cat]_2_[C_5_H_4_CS_2_], an organic cation analogue to well‐known Na_2_[C_5_H_4_CS_2_].[^53^, ^54^] There must be a mechanistic reason, why this type of reactivity and selectivity is observed only for CO_2_ and COS, despite of a higher electrophilicity of the central carbon atom at CS_2_.

### Proposed mechanism

**Scheme 6:**
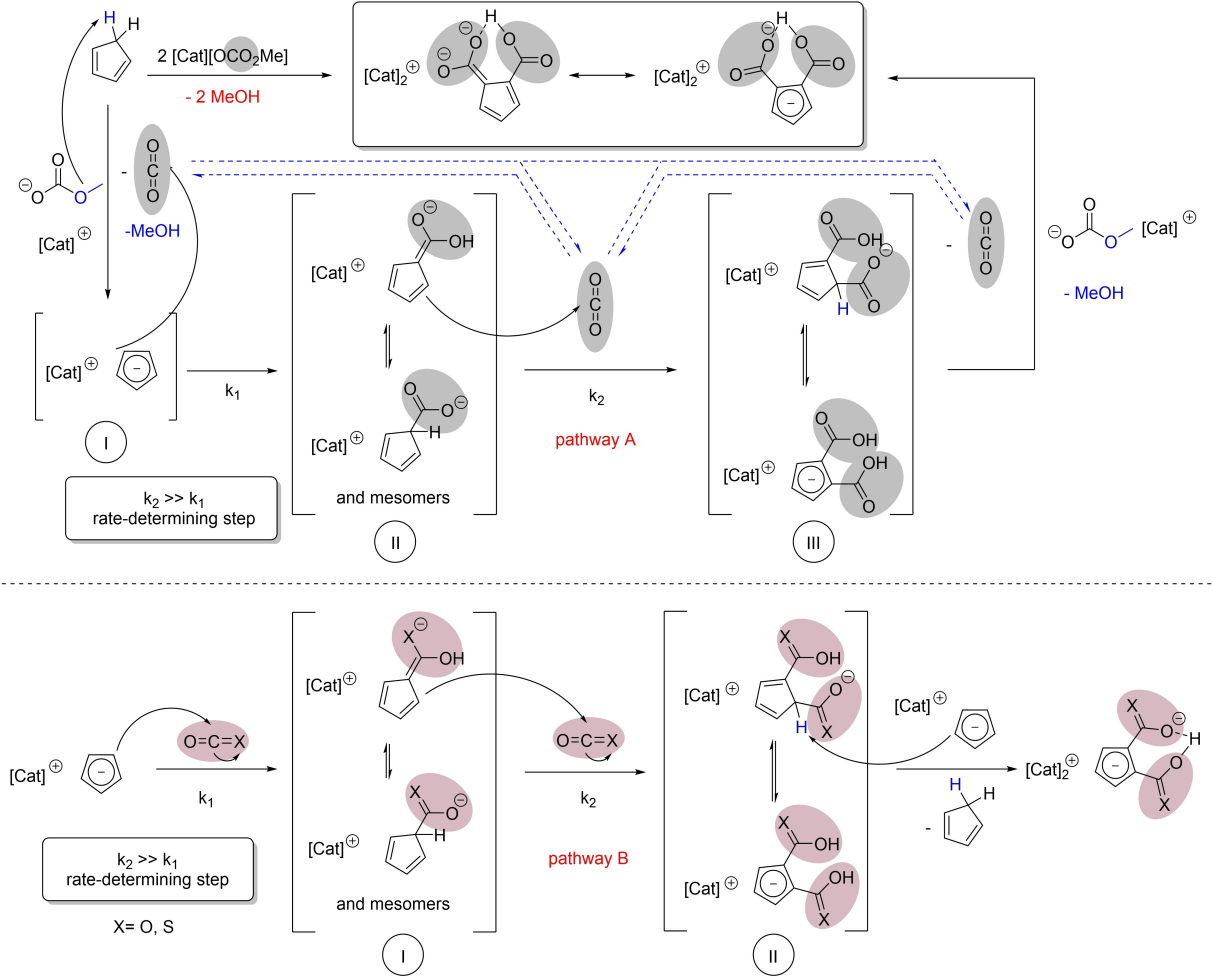
Proposed mechanism for the dicarboxylation of cyclopentadiene applying methylcarbonate anions as base (path A) and applying Cat[Cp] as nucleophile and base towards CO_2_ and COS (path B), respectively.

First, let us focus on a mechanistic discussion of the reaction of methylcarbonate anions with cyclopentadiene (Scheme 6, path A). It is suggested, that in a first step, the methylcarbonate anion acts as a base and partially or fully abstracts a proton of the CH‐acidic cyclopentadiene (I). This deprotonation equilibrium is shifted, as protonated methyl carbonate decomposes irreversibly into its components methanol and CO_2_. The reaction cannot be stopped here, we never isolated Cat[Cp] in a 1 : 1 reaction at this stage, even not with an excess of CpH. Clearly, a nucleophilic attack of the cyclopentadienyl anion at the carbon atom of dissolved CO_2_, the strongest electrophile in this system, is occurring at a rate competitive to deprotonation. A plausible, but never isolated intermediate is monocarboxylated cyclopentadienide anion II which should exist in several tautomers. Due to the electron‐withdrawing carboxylic group, it is suggested that the *O‐*protonated form of II is the more stable one in this equilibrium.

It seems plausible, that, attracted by a hydrogen bond interaction of the −COOH functionality with CO_2_ which is formed after the second carboxylation step of path A or with added COX in path B, the oxygen atoms of CO_2_ and COS act as weak proton acceptors. This hydrogen bond activates COX making it more electrophilic, at the same time this hydrogen bond directs the electrophile exclusively into the *ortho‐*position. Thus, an *ortho*‐directed, kinetically accelerated second (thio)carboxylation step with k_2_>k_1_ is occurring. When using Cat[OCO_2_Me] as carboxylating agent, a second equivalent of it is needed as base, to deprotonate intermediate III and to form the final dianionic product and CO_2_ which is available again for further nucleophilic addition. The precipitation of less soluble [Cat]_2_[C_5_H_3_(COX)_2_H] is another driver to shift these equilibria to the final product. Same intermediates are involved if Cat[Cp] is (thio)carboxylated by COX, however, a second equivalent of base Cat[Cp] (or a different base) is needed for the final deprotonation step.

**Scheme 7:**
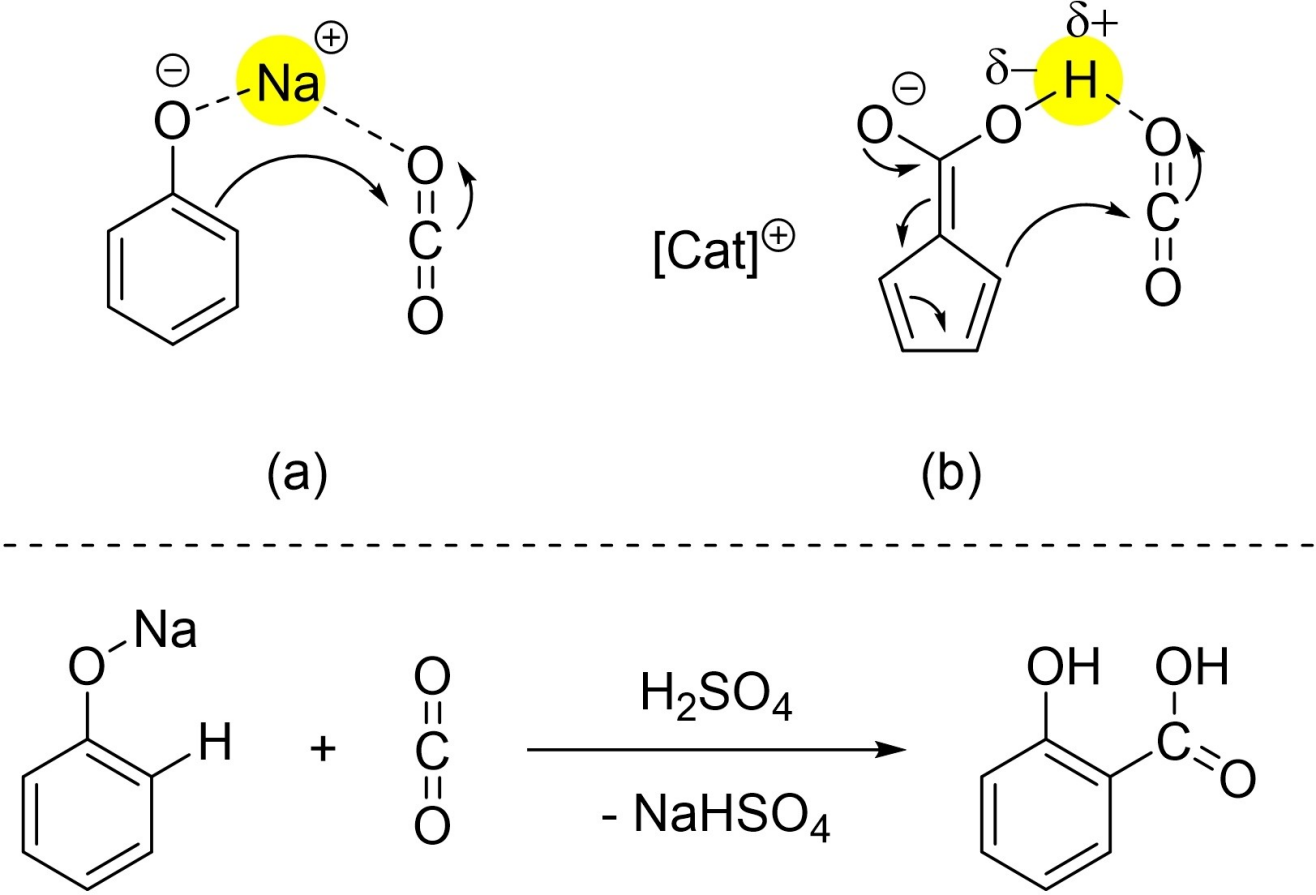
*Ortho‐*directing and activating groups in the Kolbe‐Schmitt reaction (a) and in our proposed transition state (b).[^55^, ^56^, ^57^]

This *ortho‐*directed dicarboxylation displays a remarkable resemblance to famous Kolbe‐Schmitt synthesis of salicylic acid via *ortho‐*directed carboxylation of phenolate (Scheme 7).[^55^, ^56^, ^57^] While in the archetype reaction pattern a sodium cation Na^+^ acts as acceptor towards CO_2_, a proton H^+^ is mediating, activating and directing the CO_2_ molecule in our case (Scheme 7).

The importance of such O−H⋅⋅⋅O hydrogen bond activation of COX is reflected by the fact, that CS_2_ is only inserted once, not twice into CH‐bonds of Cat[Cp] under similar conditions, plausibly a matter of weak or non‐existent S−H⋅⋅⋅S hydrogen bond interaction.[^54^, ^58^, ^59^]

In conclusion, under basic conditions we exclusively observed the *ortho‐*dicarboxylation of CpH or a di(thio)carboxylation of Cat[Cp]. Even when using a large excess of CpH in relation to the methylcarbonate salt, no monocarboxylated Cp species were detected. And even, when more than two equivalents of Cat[Cp] were used, no monocarboxylated and no tris‐ or even higher carboxylated species were detected.

### Ligand properties according to Pearson's acid‐base concept

The synthesized [Cat]_2_[C_5_H_3_(COX)_2_H] salts **1 a**–**d** and **2 a**–**c** can be used as ambident ligands bearing two coordination sites – the soft carbanionic backbone[^41^, ^42^, ^60^] and the hard “acac‐related” *O,O‐*chelating bidentate two carboxyl groups.^[41]^ Such coordination of biscarboxylated cyclopentadienes or higher carboxylated PCCPs as chelating ligand motif is unexplored. Depending on the choice of metal complex precursor, it is possible to address both coordination sites selectively. We decided to use [Mo(CO)_3_(MeCN)_3_] as precursor for a soft model Lewis acid [Mo(CO)_3_] trapping the carbanionic backbone in η^5^‐coordination mode and AlMe_3_ as precursor for the hard Lewis acid [AlMe_2_]^+^ trapping the potentially monoanionic *O,O‐*chelate ring in an “acac‐related” manner.

**Scheme 8:**
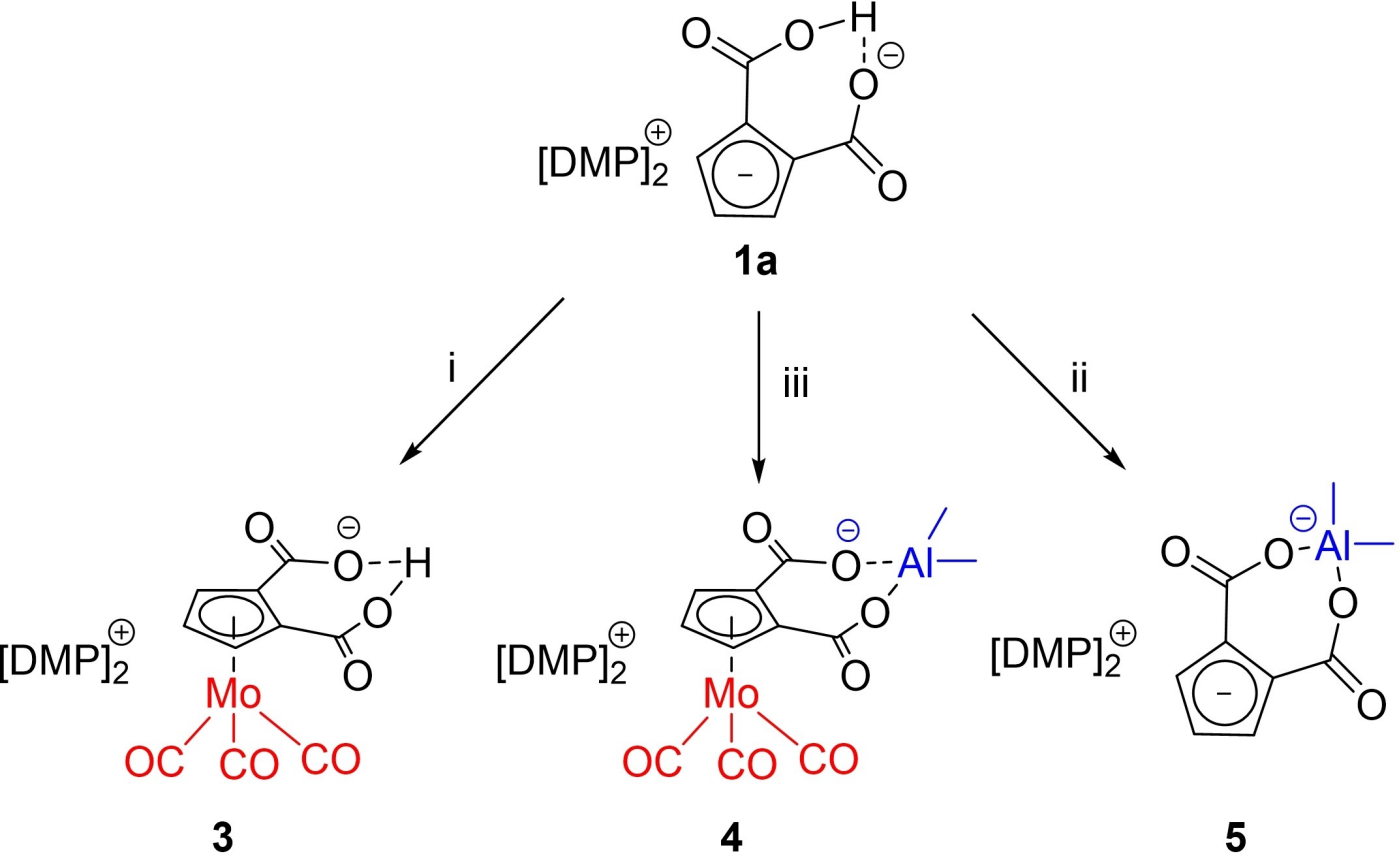
Reactivity of [DMP]_2_[C_5_H_3_(CO_2_)_2_H] (**1 a**) towards soft and hard Lewis acids. i) [Mo(CO)_3_(MeCN)_3_],MeCN, 2 h, r.t., ii) AlMe_3_ (1.03 M in toluene), toluene, 2 h, −20 °C to r.t., iii) 1. AlMe_3_ (1.03 M in toluene), toluene, 2 h, −20 °C to r.t., 2. [Mo(CO)_3_(MeCN)_3_], THF/MeCN (10 : 1), 2 h, r.t.

Reaction of [DMP]_2_[C_5_H_3_(CO_2_)_2_H] with [Mo(CO)_3_(MeCN)_3_] in MeCN for 2 h leads to [DMP]_2_[(η^5^‐C_5_H_3_(CO_2_)_2_H)Mo(CO)_3_] (**3**) while reaction of the same starting material in a toluene suspension with AlMe_3_ leads to [DMP]_2_[C_5_H_3_(CO_2_)_2_AlMe_2_] (**4**) (Scheme 8).

**Figure 4:**
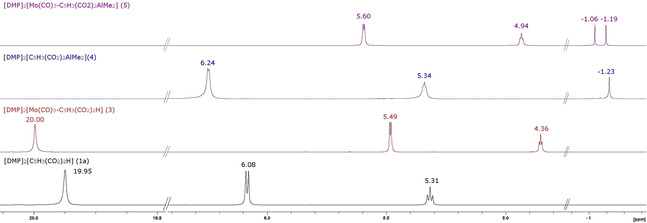
Comparison of the ^1^H NMR spectra of compounds **1 a** and **3**–**5** (DMSO‐d_6_, 300 MHz, 298 K).

Both, soft and hard Lewis acids can be combined in one dinuclear complex of ligand **1**: [DMP]_2_[C_5_H_3_(CO_2_)_2_AlMe_2_] (**4**) can be further reacted with [Mo(CO)_3_(MeCN)_3_] in THF/MeCN (10 : 1) to form complex [DMP]_2_[(η^5^‐C_5_H_3_(CO_2_)_2_AlMe_2_)Mo(CO)_3_] (**5**). The conversion can easily be monitored by ^1^H NMR spectroscopy. Figure 4 displays the characteristic shifts of **3**–**5** with DMP cations observed in DMSO‐d_6_.

The DMP cations are excluded from discussion since there is no significant shift of their signals, meaning, anions and cations are completely dissociated in DMSO‐d_6_. The Cp ring protons of [DMP]_2_[C_5_H_3_(CO_2_)_2_H] are detected in the region 4 to 6 ppm as a doublet and triplet. In non‐coordinated **1 a** they are observed at 5.31 and 6.08 ppm. In **1 a**, the acidic proton of the O−H⋅⋅⋅O IHB is deshielded and shifted towards higher frequencies at 19.95 ppm. In contrast, ring protons of [DMP]_2_[(η^5^‐C_5_H_3_(CO_2_)_2_H)Mo(CO)_3_] (**3**) are high‐field shifted (4.36 and 5.49 ppm) compared to **1 a**, a consequence of losing some degree of their aromaticity by ring η^5^‐coordination. The acidic IHB proton in **3** is shifted slightly to lower‐field, an indicator that the electron density of coordinated Cp ring is reduced and O−H⋅⋅⋅O acidity is slightly enhanced upon ring coordination to [Mo(CO)_3_]. Replacing the IHB proton of **1 a** by a [AlMe_2_]^+^ fragment in an “acac‐related” bidentate coordination mode of [DMP]_2_[C_5_H_3_(CO_2_)_2_AlMe_2_] (**4**) leads again to even slightly more low field‐shifted ring protons (5.34 and 6.24 ppm) compared to protonated form **1 a**. The chemically equivalent Al−CH_3_ groups in **4** are observed low‐field at −1.23 ppm. As expected, this equivalence is lost upon η^5^‐coordination of the ring in [DMP]_2_[(η^5^‐C_5_H_3_(CO_2_)_2_AlMe_2_)Mo(CO)_3_] (**5**): Al−CH_3_ signals are split into diasterotopic *endo* and *exo* methyl groups at −1.06 and −1.19 ppm.

The soft *S‐* and hard *O‐*donor functionality in dithiocarboxylate [DMP]_2_[C_5_H_3_(COS)_2_H] (**2 b**) is clearly differentiated in its reaction with AlMe_3_. The formation of [DMP]_2_[C_5_H_3_(COS)_2_AlMe_2_] (**6**) with *O‐*bonded aluminum is selective – as expected. The differentiation between soft *S‐* and soft *C5‐*ring coordination sites in their reaction with soft metal Lewis acids will be a challenge to be investigated in future.

**Figure 5:**
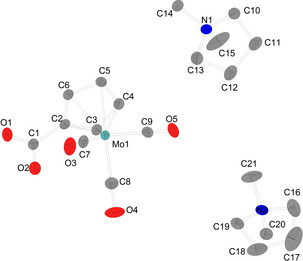
Molecular structure of [DMP]_2_[(C_5_H_4_(CO_2_))Mo(CO)_3_] (**3 a**). Hydrogen atoms and disordered atoms are neglected for clarity. Ellipsoids are shown at 50 % level. Selected bond lengths (Å) and angles (°) for a representative anion of **3 a**: Mo1−C_Centroid_ 2.0532(4), Mo1−C7 1.9304(17), O1−C1 1.2653(19), C1−O2 1.2551(19), C1−C2 1.504(2), C2−C6 1.430(2), C2−C3 1.4370(19), C3−C4 1.412(2), O3−C7 1.171(2), C4−C5 1.434(2), C5−C6 1.417(2); C8−Mo1−C9 86.86(7), O2−C1−O1 125.67(15), O2−C1−C2 117.35(14), O1−C1−C2 116.93(13), C6−C2−C3 106.61(14), C6−C2−C1 126.93(13), C3−C2−C1 126.27(13), C4−C3−C2 108.80(13), C3−C4−C5 107.96(13), C6−C5−C4 107.65(13), C5−C6−C2 108.98(13).

While **3** is fully characterized by elemental analysis and spectroscopy, we were not able to obtain refractive single crystals of **3** suitable for XRD analysis. Finally, after a few weeks of crystallization and layering the MeCN solution with ether, we isolated a few single crystals. Surprisingly, XRD analysis revealed that a fraction of partially decarboxylated **3 a** had formed (Figure 5).

**3 a** crystallizes in the monoclinic space group *P*2/*n* with Z=2 (Figure 4). As expected, the molybdenum coordination sphere can be described as *pseudo‐*tetrahedral. The Mo–C_centroid_ distance is about 2.053(4) Å which is consistent with Mo−Cp distances in literature.^[61]^ In addition to spectroscopy the presence of two cations is another indicator that the carboxylic group does not carry any proton: The free ligand may be considered as masked fulvene dianion (H_4_C_4_C=CO_2_)^2− [62]^Klicken oder tippen Sie hier, um Text einzugeben., it has not been investigated in its coordination chemistry. However, in η^5^‐coordinated form the C1−C2 distance has to be considered as C−C single bond. The twofold negative charge is delocalized in one part over the Cp ring the other in between the two oxygen atoms of the carboxyl group. Nothing is indicating any fulvene‐type bond situation in the coordinated ligand.

As the ligand salts **1 a**–**d** can be indefinitely stored in a glovebox at 25 °C and even exposure to air for 24 h does not lead to decomposition of the hygroscopic compound, the observed decarboxylation rises the question of long‐term stability of the title ligand in metal cation containing solutions. From NMR measurements of **3** in DMSO‐d_6_, it is evident, that impurities of monocarboxylated species are not present in any observable amount (>1 %). When the solid **1 b** is submitted to a TGA/DSC under protective nitrogen atmosphere, it is proven, that decomposition starts only at an offset temperature >222 °C (3 % mass loss at 10 K/min heating rate, see Supporting Information). There is no indication for a distinctive mass loss corresponding to one CO_2_ moiety.

**Scheme 9:**
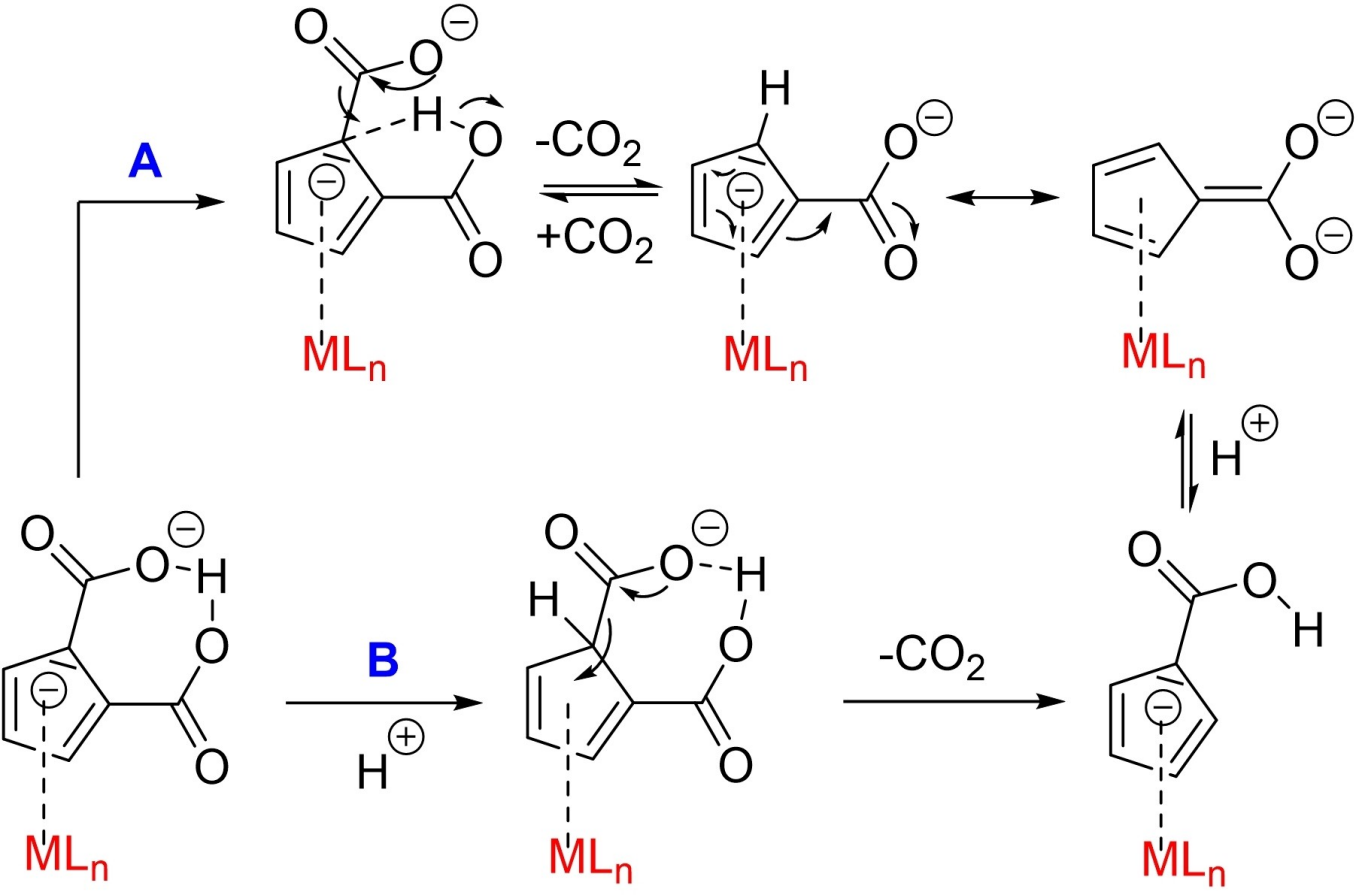
Two plausible decarboxylation paths of [C_5_H_3_(CO_2_)_2_H]^2−^ ligand in metal complexes under neutral or basic (buffered) conditions (A) or acidic conditions (B).

Two plausible decarboxylation paths of the parent dianions or of their (η^5^‐C_5_H_3_(CO_2_)_2_H)^2−^ ligand complexes are displayed in Scheme 9. Path A is plausible under neutral and basic conditions, path B is plausible under acidic conditions with extra added protons.

Path A might be induced by cleavage of the thermodynamically favoured O−H⋅⋅⋅O IHB and by alternative interaction of the acidic OH proton with the carbanionic *ortho* carbon atom in reaching distance, followed by elimination of CO_2_. Path B might be induced by direct protonation of a carbanionic ring carbon atom by extra added protons. In order to evaluate the discussed pathways, some NMR experiments were performed allowing deeper insight into the mechanism of decarboxylation: The first set of experiments was done with the non‐coordinated ligand salt **1 a** in DMSO‐d_6_. There is no detectable decarboxylation after 14 h at 25 °C, even not after additional 10 h at 60 °C, and even not after additional 14 h at 85 °C. However, after thermal treatment of **1 a** at 140 °C (5 h) we observe an unselective thermal degradation in DMSO (Supporting Information, Figure S37). We learn that trapping of traces of partially decarboxylated ligand salt by metal complexes is a rather implausible way to produce larger quantities of decarboxylated products at room temperature. If one equivalent TFA as external proton source is added to the free ligand salt **1 a** in DMSO, an immediate unselective decomposition is observed at 25 °C (Figure S36). Not unexpectedly, proton induced decarboxylation at a non‐coordinated Cp‐type ligand tends to lead to a number of products. What about decarboxylation of the [Mo(CO)_3_] coordinated, stabilized and masked carbanion? **3** does not show any decarboxylation in DMSO‐d_6_ after 14 h at 25 °C. If **3** is heated for additional 10 h at 60 °C, a first NMR signature of trace amounts of decarboxylated **3 a** is occurring (Figure S38), the signals become more prominent after another 14 h at 85 °C. But even after another 24 h at 85 °C the highly selective decarboxylation towards **3 a** is not progressing. In the closed system of a carefully sealed NMR tube a carboxylation / decarboxylation equilibrium has obviously been reached. We conclude that masking the carbanion by coordination to [Mo(CO)_3_] is leading to delocalization of the negative ring charge, a ring stabilization and a kinetic acceleration of a selective decarboxylation path towards **3 a** compared to the thermal process of the free ligand anion. Is this selective path A in fact induced by the available proton of the IHB? Indeed, if the IHB proton of **3** is substituted by a [AlMe_2_]^+^ moiety in **5**, much to our surprise, we do not observe any decarboxylation under identical thermal conditions as observed for **3 a** (Figure S39). From these experiments we learn that a selective decarboxylation path requires the intramolecularly available proton of the IHB. By heating, this buffered proton seems to be leaving its thermodynamically favored chelating position and is transferred to a ring carbanion position in reaching distance. This proton shift (first step of path A) plausibly induces a selective decarboxylation path. In a closed system without escaping CO_2_ this decarboxylation equilibrium does not proceed to full conversion, however, less soluble monocarboxylated species begin to crystallize from the mixture.

**Scheme 10:**
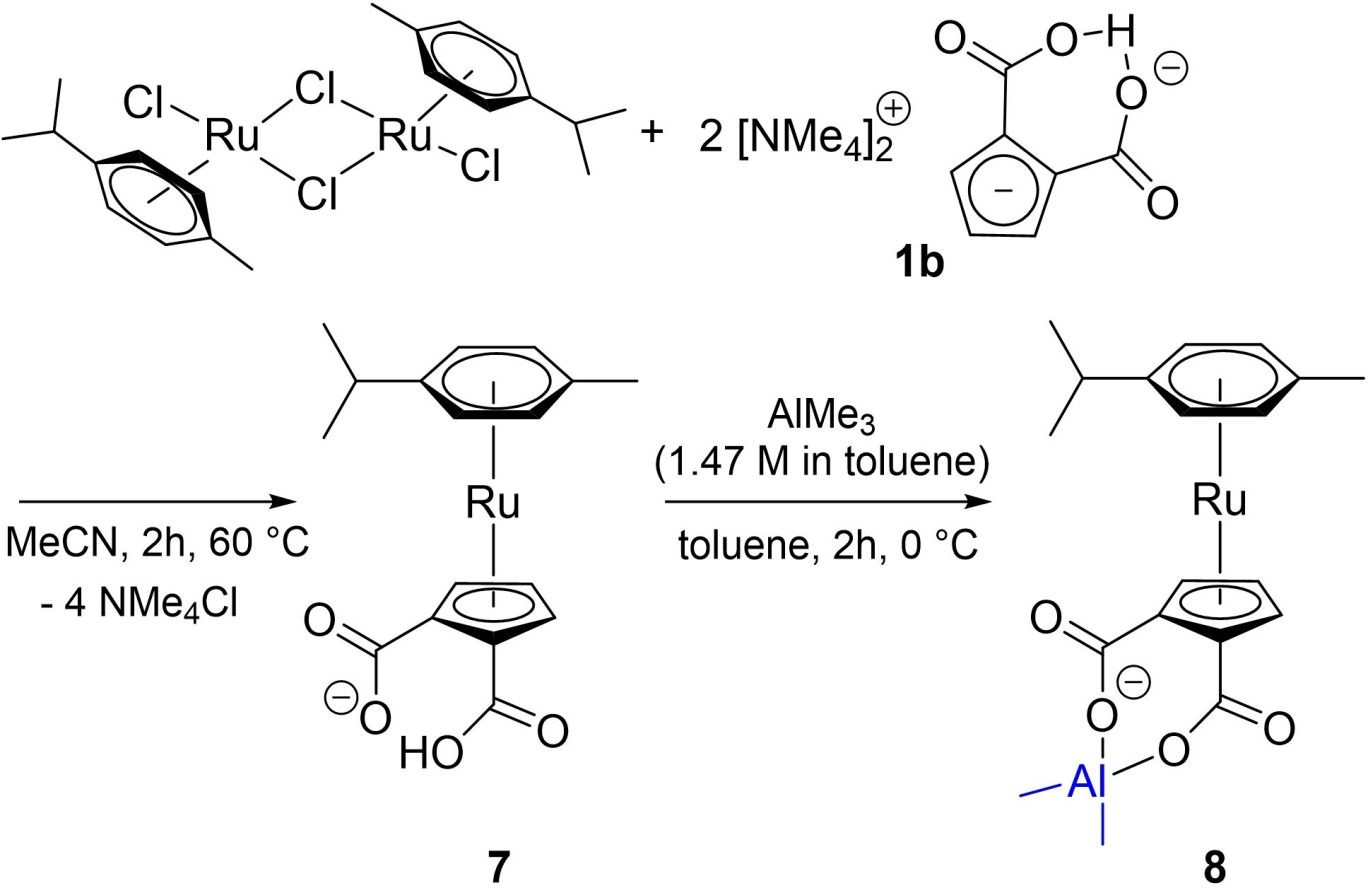
Synthesis of [(η^6^‐p‐cymene)Ru(η^5^‐C_5_H_3_(CO_2_)_2_H)] (**7**) and [(η^6^‐p‐cymene)Ru(η^5^‐C_5_H_3_(CO_2_)_2_AlMe_2_)] (**8**).

Furthermore, we realized the synthesis of an uncommon non‐charged [CpRu(arene)] sandwich complexes **7** and **8** (Scheme 10). Rather common cationic complexes [CpRu(arene)]^+^ are typically obtained either by arene exchange in labile naphthalene complex [CpRu(C_10_H_8_)]^+[63]^ or by introducing a Cp anion at [(arene)RuCl_2_]_2_ in the presence of weakly coordinating and exchanged anions.^[64]^ In our approach p‐cymene‐ruthenium dichloride dimer is simply reacted with Cp dianion synthon [NMe_4_]_2_[[C_5_H_3_(CO_2_)_2_H] (**1 b**) (Scheme 10).

**Figure 6:**
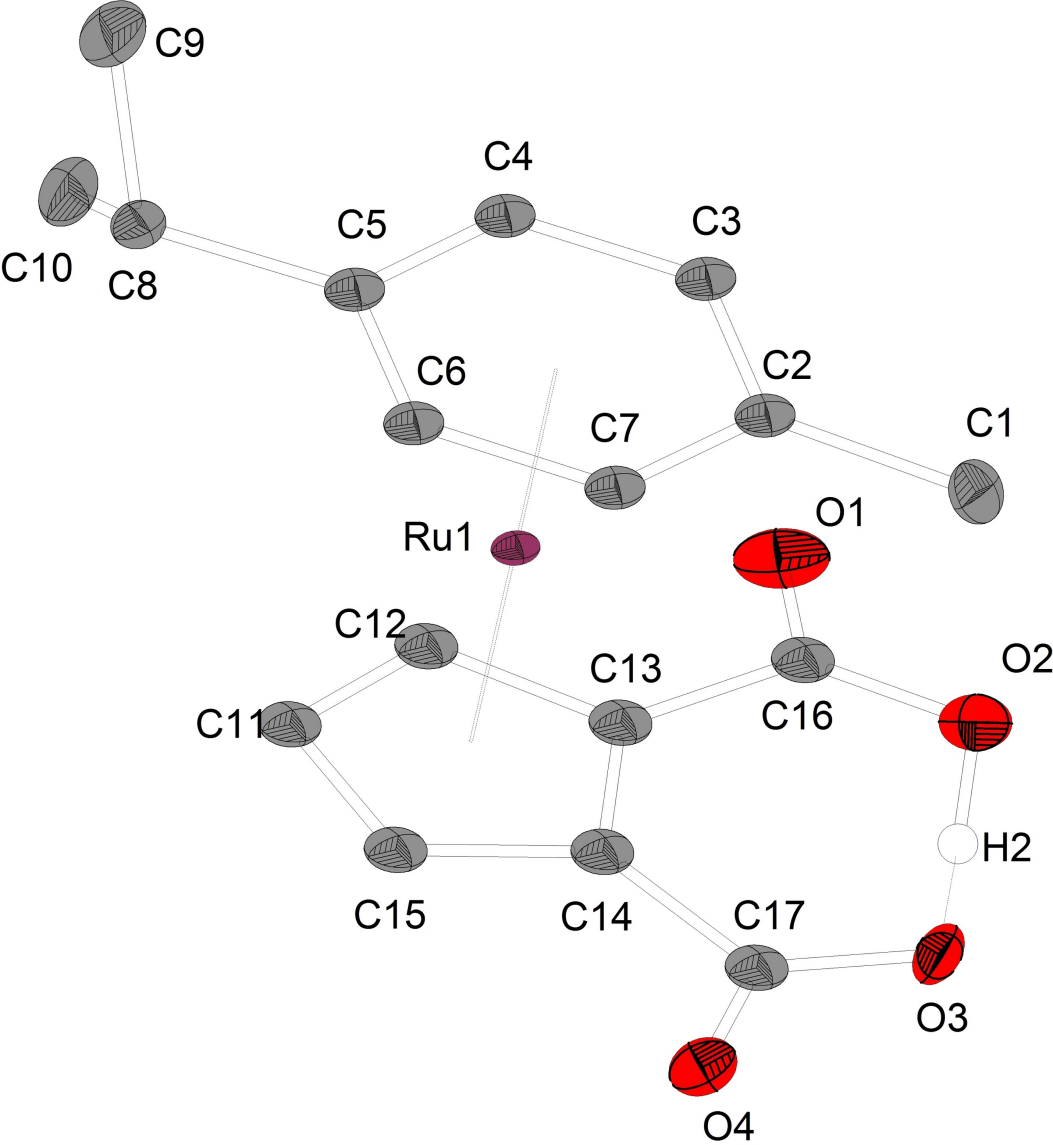
Molecular structure of [(η^6^‐p‐cymene)Ru(η^5^‐C_5_H_3_(CO_2_)_2_H)] (**7**). Hydrogen atoms and disordered atoms are neglected for clarity. Ellipsoids are shown at 50 % level. Selected bond lengths (Å) and angles (°) for a representative anion of **7**: O2−H2 1.1324(463), O3−H2 1.3008(453), Ru1−C_centroid_ (Cp) 1.8178(6), Ru1−C_centroid_ (p‐cymene) 1.6994(6), O1−C16 1.206(7), C1−C2 1.505(7), O2−C16 1.305(6), O2−H2 1.14(3), C2−C3 1.413(8), C2−C7 1.432(8), C13−C16 1.519(7), C17−C14 1.501(7), O4−C17−O3 125.5(5), O4−C17−C14 118.5(5), O3−C17−C14 116.0(5), O1−C16−O2 124.3(5), O1−C16−C13 119.3(5), O2−C16−C13 116.4(4).

The neutral sandwich complex [(η^6^‐p‐cymene)Ru(η^5^‐C_5_H_3_(CO_2_)_2_H)] (**7**) was extracted with 1,2‐dichloroethane while the precipitated [NMe_4_]Cl could simply be filtered off. Single crystals suitable for XRD analysis of [(η^6^‐p‐cymene)Ru(η^5^‐C_5_H_3_(CO_2_)_2_H)] (**7**) were obtained by vapour diffusion of diethyl ether into a saturated solution of the target compound in MeCN. **7** crystallizes in the monoclinic space group *P*2_1_ with Z=4 (Figure 6).

The C17−O4 and C16−O1 distances indicate a more pronounced double bond character (1.207(7); 1.206(7) Å) and the C17−O3 and C16−O2 distances increased single bond character (1.296(6); 1.306(7) Å) compared to the molecular structure of **1 a**, respectively. The C_centroid_−Ru bond to the p‐cymene ligand (1.6994(6) Å) is shorter than the C_centroid_−Ru to the Cp ring (1.8178(6) Å) which show the better π‐backbonding of the p‐cymene ligand compared to Cp rings.

**Scheme 11:**
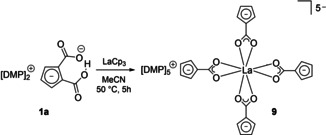
Unexpected formation of [DMP]_5_[La(κ2‐O_2_C−C_5_H_4_)_4_] (**9**) via a decarboxylation.

The protolytically reactive carboxyl groups of **7** were again addressed by reaction with AlMe_3_ in toluene yielding a dinuclear complex [(η^6^‐p‐cymene)Ru(η^5^‐C_5_H_3_(CO_2_)_2_AlMe_2_)] (**8**) selectively. With the challenging goal to simply replace main group Lewis acid fragment [Me_2_Al]^+^ in **8** by lanthanocene fragment [Cp_2_La]^+^ we also investigated the reaction of [DMP]_2_[C_5_H_3_(CO_2_)_2_H] with LaCp_3_ instead of AlMe_3_ under similar conditions. However, the expected lanthanocene [DMP]_2_[C_5_H_3_(CO_2_)_2_LaCp_2_] was not isolated as an identified product. Instead of expected protolytic elimination of CpH and inserting an intact lanthanocene cation unit into the O−H−O IHB, a completely unexpected carboxylato lanthanate [DMP]_5_[La(κ^2^‐O_2_C−C_5_H_4_)_4_] (**9**), again a product of decarboxylation, was formed in good and reproducible yield 69 % (calcd. on basis of La, Scheme 11).

Similar to **3 a**, but at much higher and reproducible yields, interaction of **1 a** with Lewis acids seems to open a metal mediated decarboxylation path resulting in coordination of the so far unexplored masked fulvene dianion (H_4_C_4_C=CO_2_)^2−^ towards metals. In contrast to **3 a**, this dianionic ligand is selectively bonded via the carboxylate groups leaving the anionic cyclopentadienyl ligand part non‐bonded. We propose, that this penta‐anion (**9**) will be a nice template to bind preferably cationic Cp anion receptor complex fragments into heteronuclear complex arrays. This strategy will be investigated in more detail. It should be noted, that decarboxylation of multi‐carboxylated Cp species to monocarboxylated^[33]^ or at least less carboxylated Cp rings has been observed in literature.[^30^, ^46^, ^48^, ^49^] On the other hand, Evan and coworkers demonstrated that CO_2_ readily inserts into metal carbon bonds of organo lanthanides LnCp_3_ to form carboxylate ligands.^[65]^

**Figure 7:**
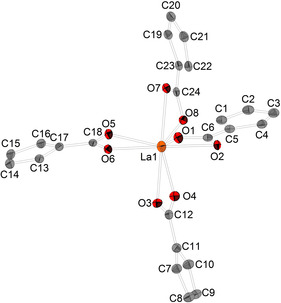
Molecular structure of [DMP]_5_[La(κ^2^‐O_2_C−C_5_H_4_)_4_] (**9**). Cations, hydrogen atoms and disordered atoms are neglected for clarity. Ellipsoids are shown at 50 % level. Selected bond lengths (Å) and angles (°) for a representative anion of **9**: La1−O1 2.507(6), La1−O2 2.562(5), La1−C6 2.920(9), O1−C6 1.292(11), O2−C6 1.284(10), C1−C2 1.425(12), C2−C3 1.423(15), C3−C4 1.402(14), 1.405(13), C1−C5 1.425(12), C5−C6 1.448(12), O1−La1−O8 126.6(2), O1−La1−O5 82.06(19), O8−La1−O5 123.4(2), O8−La1−O3 134.22(18), O5−La1−O3 91.2(2), O8−La1−O7 52.08(19), O3−La1−O7 172.70(18), C6−O1−La1 95.0(5), C6−O2−La1 92.7(5), O2−C6−O1 119.6(8).

Single crystals suitable for XRD analysis of [DMP]_5_[La(κ^2^‐O_2_C−C_5_H_4_)_4_] (**9**) were obtained by cooling a saturated solution of the target compound in MeCN to 0 °C. **9** crystallizes in the monoclinic space group *P*2_1_/*c* with Z=4 (Figure 7).

The coordination sphere can be considered as distorted square. In contrast to some known metal complexes with cyclopentadienedithiocarboxylate (C_5_H_4_CS_2_)^2−^ ligand^[58]^, the C5‐C6 bond has to be described as single bond (1.45(1) Å) and with respect to the La−O bond lengths (La1−O1 2.507(6) Å, La1−OO2 2.562(5) Å) and ring C−C bond lengths (average 1.407 Å) in the molecular structure, it is plausible, that one electron of this dianion is delocalized over the CO_2_ moiety while the other is allocated over the Cp backbone. The Cp backbone is only slightly out of the plain defined by the CO_2_ moiety.

## Conclusions

We reported a highly regioselective 1,2‐dicarboxylation and 1,2‐dithiocarboxylation of probably the most important carbanionic ligand (C_5_H_5_)^−^ of organometallic chemistry. Two different synthetic approaches towards the same targets are introduced involving either direct carboxylation of classical CH‐acid Cp−H by a number of organic cation methylcarbonate ionic liquids and salts Cat[OCO_2_Me], or via reaction of Cat[C_5_H_5_] with CO_2_ and COS. These simple one‐pot transformations are limited to oxygen containing synthons CO_2_ and COS, they do not occur with the more electrophilic but much less pronounced proton acceptor CS_2_. Therefore, we discussed an interaction of the first C−H inserted (thio)carboxylate group via an Cp−C(X)O−H^+^⋅⋅⋅O=C=X (X=O, S) hydrogen bond as activating and strictly *ortho‐*directing mechanistic principle explaining the characteristic kinetically accelerated second (thio)carboxylation step. It bears some resemblance to famous Kolbe‐Schmitt synthesis of salicylic acid via *ortho‐*directed carboxylation of phenolate via Ph−O−Na^+^⋅⋅⋅O=C=O interaction. The dianionic ligand salts [Cat]_2_[C_5_H_3_(COX)_2_H] **1 a**–**d** and **2 a**–**c** (Cat=DMP^+^, NMe_4_^+^, EMMIm^+^, PPh_4_^+^) were fully characterized including XRD analyses. They reveal a characteristic intramolecular O−H⋅⋅⋅O hydrogen bond. In a preliminary study, it was proven, that soft cyclopentadienyl and hard O‐donor sites of these ambident ligands can selectively be addressed by soft Lewis acids [Mo(CO)_3_] or [(aren)Ru]^2+^ and by hard Lewis acids [Me_2_Al]^+^ and La^3+^. Therefore, such ligands can be used as templates for heterodinuclear complexes such as [DMP]_2_[(η^5^‐C_5_H_3_(CO_2_)_2_AlMe_2_)Mo(CO)_3_] (**5**) and [(η^6^‐p‐cymene)Ru(η^5^‐C_5_H_3_(CO_2_)_2_AlMe_2_)] (**8**). In some reactions with metal Lewis acids, decarboxylation of ligand **1** was observed. The latter is leading to complexes of unexplored ligand dianion (H_4_C_5_CO_2_)^2−^, which was XRD structurally characterized as π‐*C‐*donor in [DMP]_2_[(η^5^‐C_5_H_4_CO_2_)Mo(CO)_3_] (**3 a**) or as σ‐*O‐*donor in [DMP]_5_[La(κ^2^‐O_2_C−C_5_H_4_)_4_] (**9**). We are convinced, that our simple and selective access to this class of reactive and transferable C, O, and S ligands will establish a diversity of further transformations of **1**‐ and **2**‐type dianions. These will be of interest for designing mono‐ and heterodinuclear catalysts with cooperating Lewis acid and/or protic acid functionality or for water‐soluble active agents beyond the well‐investigated reactivity patterns of cyclopentadiene carboxylate esters and off the beaten track of stable ferrocene carboxylic acids.

Deposition Number(s) 2055719 (for **1 a** ⋅ MeCN ), 2055720 (for **1 a** ⋅ MeOH ), 2055723 (for **2**), 2055722 (for **3 a**), 2055721 (for **7**), and 2055724 (for **9**) contain(s) the supplementary crystallographic data for this paper. These data are provided free of charge by the joint Cambridge Crystallographic Data Centre and Fachinformationszentrum Karlsruhe Access Structures service www.ccdc.cam.ac.uk/structures.

## Conflict of interest

The authors declare no conflict of interest.

## Supporting information

As a service to our authors and readers, this journal provides supporting information supplied by the authors. Such materials are peer reviewed and may be re‐organized for online delivery, but are not copy‐edited or typeset. Technical support issues arising from supporting information (other than missing files) should be addressed to the authors.

Supplementary
