# Supplementary material for: Highly Selective Ortho‐Directed Dicarboxylation of Cyclopentadiene by Methylcarbonates and CO2 or COS – First Insight into Co‐ordination Chemistry of New Ambident Ligands
Source: Chemistry. 2021 May 13;27(33):8517–27. doi: 10.1002/chem.202100300 (PMC8252773; doi:10.1002/chem.202100300)
Supplement: Supplementary file 1 — Supplementary [file CHEM-27-8517-s001.pdf]

Chemistry–A European Journal

Supporting Information

Highly Selective Ortho-Directed Dicarboxylation of Cyclopentadiene by Methylcarbonates and CO_2 or COS – First Insight into Co-ordination Chemistry of New Ambident Ligands

Tobias Vollgraff and Jörg Sundermeyer*

Table of contents

1. Experimental section 2
2. NMR spectra 4
   a. $[DMP]_2[C_5H_3(CO_2)_2H]$ (**1a**) 4
   b. $[NMe_4]_2[C_5H_3(CO_2)_2H]$ (**1b**) 5
   c. $[EMMIm]_2[C_5H_3(CO_2)_2H]$ (**1c**) 6
   d. $[PPh_4]_2[C_5H_3(CO_2)_2H]$ (**1d**) 7
   e. $[DMP]_2[C_5H_3(COS)_2H]$ (**2a**) 8
   f. $[NMe_4]_2[C_5H_3(COS)_2H]$ (**2b**) 9
   g. $[PPh_4]_2[C_5H_3(COS)_2H]$ (**2c**) 10
   h. $[DMP]_2[(\eta^5\text{-}C_5H_3(CO_2)_2H)Mo(CO)_3]$ (**3**) 11
   i. $[DMP]_2[C_5H_3(CO_2)_2AlMe_2]$ (**4**) 12
   j. $[DMP]_2[(\eta^5\text{-}C_5H_3(CO_2)_2AlMe_2)Mo(CO)_3]$ (**5**) 13
   k. $[DMP]_2[C_5H_3(COS)_2AlMe_2]$ (**6**) 14
   l. $[\eta^6$-p-cymene-Ru-$\eta^5\text{-}C_5H_3(CO_2)_2H]$ (**7**) 15
   m. $[\eta^6$-p-cymene-Ru-$\eta^5\text{-}C_5H_3(CO_2)_2AlMe_2]$ (**8**) 16
   n. $[DMO]_5[La(C_5H_4CO_2)_4]$ (**9**) 17
3. Single crystal x-ray structures 18
   a. Crystal Data 18
   b. Molecular structures of the title compounds 21
4. Thermogravimetric measurement of $[NMe_4]_2[(C_5H_3(CO_2)_2H]$ (1b) 24
5. Acidic and thermal decarboxylation study of 1a, 3 and 5 25
6. References 27

1. Experimental section

Methods and devices

All preparative operations were conducted by using standard Schlenk techniques and freshly dried solvents. All solvents were dried according to common procedures[1] and passed through columns of aluminium oxide, 3 Å molecular sieves and R3-11G-catalyst (BASF) or stored over molecular sieves (3 Å or 4 Å). Cyclopentadiene was freshly distilled. Other reagents were used as received unless stated otherwise.

Elemental analyses (C, H, N, S) were carried out by the service department for routine analysis with a vario MICRO cube (Elementar). Samples for the elemental analysis were weighted into tin capsules inside a nitrogen filled glovebox (slightly higher N values). ^{1}H and proton decoupled ^{13}C-NMR spectra were recorded in automation or by the service department with a Bruker Avance II 300 spectrometer and a Bruker Avance II HD 300 spectrometer. All spectra were recorded at ambient temperature. ^{1}H and ^{13}C NMR spectra were calibrated using residual proton signals of the solvent (DMSO-d$_6$: δ_H = 2.50 ppm, δ_C = 39.52 ppm). Multiplicity is abbreviated as follows: s (singlet), d (doublet), t (triplet), q (quartet), m (multiplet), br. IR spectra were recorded on a Bruker APLPHA FT-IR spectrometer with Platinum ATR sampling (diamond single crystal). High-resolution LIFDI mass spectra were acquired with an AccuTOF-GCv TOF mass spectrometer (JEOL). TGA measurements starting at 25°C were conducted with a DSC-TGA 3 (Mettler Toledo) in a nitrogen filled glovebox. Decomposition temperatures were determined monitoring synchronously DSC data.

Synthesis of title compounds

Synthesis of [DMP]$_2$[C$_5$H$_3$(CO$_2$)$_2$H] (1) Procedure A. [DMP][OCO$_2$Me] (1.00 g, 5.71 mmol, 2.0 equiv.) was solved in MeCN (10 mL). CpH (190 mg, 236 µL, 2.89 mmol, 1.0 quiv.) was added and the solution was stirred for 2h at 50 °C. During the reaction the solution turned purple and a colourless precipitate was formed. After cooling the reaction to -20 °C the precipitate was filtered off, was washed with THF (10 mL) and pentane (10 mL) and dried *in vacuo*. [DMP]$_2$[C$_5$H$_3$(CO$_2$)$_2$H] was obtained as a colourless solid in a yield of 855 mg (4.85 mmol, 85%). Colourless needles of X-ray diffraction quality were obtained by cooling the reaction solution slowly down to r.t. overnight or by storing the filtrate at -20 °C. **Procedure B.** DMP[Cp] (1.00 g, 6.05 mmol, 2.0 equiv.) made from [DMP]Cl and NaCp following a modified procedure of Harder and co-workers[2] was dissolved in DMF (10 mL). An excess of CO$_2$ was

bubbled into the solution for 5 minutes. The deep brown solution immediately turned to a lighter brown. The solvent was removed and the residue was dissolved in MeCN (10 mL) and after warming for some minutes and sonification the white powder was filtered and worked-up in the same way described in Procedure A. $[DMP]_2[C_5H_3(CO_2)_2H]$ was obtained as a colourless solid in a yield of 694 mg (3.94 mmol, 69%). The yield can be increased by cooling the filtrate to -20 °C overnight. IR (neat): $\tilde{\nu}_{max}$= 3091 (w), 3064 (w), 3003 (w), 2890 (w), 1658 (w), 1465 (m), 1398 (m), 1366 (m), 1339 (m), 1273 (m), 1260 (w), 1102 (m), 1037 (m), 1005 (m), 979 (m), 934 (m), 893 (w), 823 (m), 795 (m), 739 (s), 562 (w), 511 (m), 432 (w) cm^{-1}; ^{1}H NMR (300.1 MHz, DMSO-d_6): δ_H = 2.04 (br s, 4H, CH_2), 3.02 (s, 6H, NMe_2), 3.38 (br s, 4H, NCH_2), 5.32 (t, $^{3}J_{HH}$= 3.3 Hz, 1H, Cp*H*), 6.08 (d, $^{3}J_{HH}$ = 3.3 Hz, 2H, Cp*H*), 19.95 (s, 1H, COO*H*) ppm. ^{13}C NMR (75.5 MHz, DMSO-d_6): δ_C = 21.3 (2C, *C*H_2), 50.7 (t,$^{1}J_{CN}$= 3.9 Hz, 2C, NMe_2), 64.5 (t, $^{1}J_{CN}$= 3.1 Hz, 2C, N*C*H_2), 105.9 (2C, Cp), 115.3 (2C, Cp), 116.8 (1C, Cp), 170.9 (1C, *C*OOH) ppm; elemental analysis calcd (%) for $C_{19}H_{32}N_2O_4$ (352.48 g mol^{-1}): C 64.74; H 9.15; N 7.95; found: C 64.43; H 9.07; N 7.82.

Synthesis of $[DMP]_2[C_5H_3(COS)_2H]$ (2a). DMP[Cp] (1.00 g, 6.05 mmol, 2.0 equiv.) made from [DMP]Cl and NaCp following a modified procedure of Harder and co-workers[2] was dissolved in DMF (10 mL). An excess of COS was condensed into a the Schlenk tube at -196 °C with the help of a condensation line. The frozen DMF solution was warmed up to -56 °C (dry ice/n-octane) where the mixture liquidized again. After 1h, the deep brown solution turned yellow. The solvent was removed and the residue was dissolved in MeCN (10 mL) and after warming for some minutes and sonification the yellow solid was filtered, washed with THF (10 mL) and n-pentane (10mL) and dried in vacuo. $[DMP]_2[C_5H_3(COS)_2H]$ was obtained as a yellow solid in a yield of 628 mg (1.63 mmol, 54%). The yield can be increased by cooling the filtrate to -20 °C overnight.

Yellow needles of X-ray diffraction quality were obtained by layering a saturated solution of (**2**) in MeCN with Et_2O at r.t. IR (neat): $\tilde{\nu}_{max}$= 3002 (w), 2890 (w), 1506 (w), 1458 (m), 1384 (m), 1354 (m), 1230 (s), 1117 (s), 1098 (m), 1036 (m), 999 (vs), 934 (m), 876 (s), 834 (vs), 761(m), 742 (vs), 704 (s), 680 (m), 579 (s), 557 (m), 514 (m), 464 (w), 446 (m), 435 (s), 420 (s) cm^{-1}; ^{1}H NMR (300.1 MHz, DMSO-d_6): δ_H = 2.05 (br s, 4H, CH_2), 3.05 (s, 6H, NMe_2), 3.40 (br s, 4H, NCH_2), 5.47 (t, $^{3}J_{HH}$ = 3.6 Hz, 1H, Cp*H*), 6.88 (d, $^{3}J_{HH}$ = 3.6 Hz, 2H, Cp*H*), 18.89 (s, 1H, CSO*H*) ppm. ^{13}C NMR (75.5 MHz, DMSO-d_6): δ_C = 21.3 (2C, *C*H2), 50.8 (t,$^{1}J_{CN}$= 3.9 Hz, 2C, N*Me2*), 64.6 (t, $^{1}J_{CN}$= 3.1 Hz, 2C, N*C*H_2), 110.8 (2C, Cp), 126.5 (2C, Cp), 128.0 (1C, Cp),

206.9 (1C, *C*SOH) ppm; elemental analysis calcd (%) for $C_{19}H_{32}N_2O_2S_2$ (384.60 g mol^{-1}): C 59.34; H 8.39; N 7.28; found: C 58.97, H 8.17, N 7.13.

Synthesis of [DMP]$_2$[(η^5-$C_5H_3(CO_2)_2$H)Mo(CO)$_3$] (3). [DMP]$_2$[$C_5H_3(CO_2)_2$H] (100mg, 0.28 mmol, 1.0 equiv.) was slightly dissolved in MeCN (10 mL) and Mo(CO)$_3$(MeCN)$_3$ (86 mg, 0.28 mmol, 1.0 equiv.), dissolved in MeCN (10 mL) was added. After 2h stirring at r.t. all solids were dissolved and the solvent was removed *in vacuo*. The yellow residue was suspended and sonicated in Et_2O (20 mL) before the solvent was removed by syringe and the solid was finally dried in vacuo again. [DMP]$_2$[($\eta^5$$C_5H_3(CO_2)_2$H)Mo(CO)$_3$] was obtained as yellow solid in quantitative yield. IR (neat): $\tilde{\nu}_{max}$= 3026 (w), 2969 (w), 1896 (vs), 1763 (vs), 1581 (m), 1469 (m), 1442 (m), 1355 (m), 1323 (m), 1277 (w), 1197 (m), 1093 (m), 1026 (w), 1001 (m), 933 (w), 836 (w), 783 (m), 615 (m), 516 (m), 430 (m) cm^{-1}; ^{1}H NMR (300.1 MHz, DMSO-d$_6$): δ_H = 2.11 (br s, 4H, C*H*$_2$), 3.10 (s, 6H, N*Me*$_2$), 3.47 (br s, 4H, NC*H*$_2$), 4.86 (t, $^3J_{HH}$= 3.0 Hz, 1H, Cp*H*), 5.49 (d, $^3J_{HH}$ = 3.0 Hz, 2H, Cp*H*), 20.01 (s, 1H, COO*H*) ppm. ^{13}C NMR (75.5 MHz, DMSO-d$_6$): δ_C = 21.4 (2C, *C*H$_2$), 51.0 (t,$^1J_{CN}$ = 4.0 Hz, 2C, N*Me*$_2$), 64.8 (t, $^1J_{CN}$= 3.2 Hz, 2C, N*C*H$_2$), 84.7 (2C, Cp), 93.7 (2C, Cp), 98.4 (1C, Cp), 168.0 (1C, *C*OOH), 233.8 (3C, Mo(*C*O)$_3$) ppm; elemental analysis calcd (%) for $C_{22}H_{32}MoN_2O_7$ (532.47 g mol^{-1}): C 49.63; H 6.06; N 5.26; found: C 49.51, H 5.98, N 5.27.

Synthesis of [DMP]$_2$[$C_5H_3(CO_2)_2$AlMe$_2$] (4). [DMP]$_2$[$C_5H_3(CO_2)_2$H] (100mg, 0.28 mmol, 1.0 equiv.) was suspended in toluene (10 mL) and $AlMe_3$ (1.03 M in toluene, 0.28 mL, 0.28 mmol, 1.0 equiv.) was added at -20 °C. After 2h stirring at r.t. the methane evolution stopped, the solvent was removed *in vacuo*. The colourless residue was suspended and sonicated in pentane (20 mL) before the solvent was removed by syringe and the solid was finally dried in vacuo again. [DMP]$_2$[$C_5H_3(CO_2)_2$AlMe$_2$] was obtained as colourless solid in quantitative yield. IR (neat): $\tilde{\nu}_{max}$= 3076 (w), 3020 (w), 2916 (w), 2814 (w), 1568 (s), 1470 (m), 1412 (m), 1383 (m), 1345 (w), 1301 (m), 1228 (m), 1205 (w), 1167 (m), 1086 (m), 1045 (m), 1004 (w), 979 (w), 936 (w), 881 (m), 835 (m), 791 (w), 737 (m), 682 (w), 652 (w), 568 (m), 520 (w), 451 (w), 431 (m) cm^{-1}; ^{1}H NMR (300.1 MHz, DMSO-d$_6$): δ_H = -1.23 (s, 6H, Al*Me*2), 2.06 (br s, 4H, C*H*$_2$), 3.03 (s, 6H, N*Me*$_2$), 3.39 (br s, 4H, NC*H*$_2$), 5.34 (t, $^3J_{HH}$ = 2.9 Hz, 1H, Cp*H*), 6.24 (d, $^3J_{HH}$ = 2.9 Hz, 2H, Cp*H*) ppm. ^{13}C NMR (75.5 MHz, DMSO-d$_6$): δ_C = -8.30 (2C, Al*Me*$_2$), 21.3 (2C, *C*H$_2$), 50.8 (t,$^1J_{CN}$= 4.0 Hz, 2C, N*Me*$_2$), 64.6 (t, $^1J_{CN}$= 3.1 Hz, 2C, N*C*H2), 106.3 (2C, Cp), 93.7 (2C, Cp), 119.1 (1C, Cp), 168.8 (1C, *C*OO-

$AlMe_2$) ppm; elemental analysis calcd (%) for $C_{21}H_{37}AlN_2O_4$ (408.52 g mol^{-1}): C 61.74; H 9.13; N 6.86; found: C 61.31, H 9.02, N 6.86.

Synthesis of [DMP]$_2$[(η^5-$C_5H_3(CO_2)_2AlMe_2$)Mo(CO)$_3$] (5). [DMP]$_2$[$C_5H_3(CO_2)_2AlMe_2$] (116 mg, 0.28 mmol, 1.0 equiv.) was prepared according to the described procedure and was suspended with $Mo(CO)_3(MeCN)_3$ (86 mg, 0.28 mmol, 1.0 equiv.) in THF (20 mL). MeCN (2 mL) was added. The solution turned orange and after 1 h stirring at r.t. the solvent was removed *in vacuo*. The orange residue was suspended and sonicated in Et_2O (20 mL) before the solvent was removed by syringe and the solid was finally dried *in vacuo* again. [DMP]$_2$[(η^5- $C_5H_3(CO_2)_2AlMe_2$)Mo(CO)$_3$] was obtained as yellow solid in quantitative yield. IR (neat): $\tilde{\nu}_{max}$= 3025 (w), 2920 (w), 1892 (vs), 1760 (vs), 1639 (s), 1608 (m), 1473 (m), 1444 (m), 1416 (m), 1393 (w), 1361 (w), 1317 (m), 1195 (m), 1088 (w), 1040 (m), 1000 (m), 976 (m), 934 (w), 828 (m), 783 (m), 694 (m), 666 (m), 641 (w), 612 (m), 576 (w), 536 (w), 510 (m), 457 (m) cm^{-1}; ^{1}H NMR (300.1 MHz, DMSO-d$_6$): δ_H = -1.18 (s, 3H, Al*Me*$_2$), -1.06 (s, 3H, Al*Me*$_2$), 2.11 (br s, 4H, C*H*$_2$), 3.09 (s, 6H, N*Me*$_2$), 3.46 (br s, 4H, NC*H*$_2$), 4.94 (t, $^3J_{HH}$ = 2.9 Hz, 1H, Cp*H*), 5.60 (d, $^3J_{HH}$ = 2.9 Hz, 2H, Cp*H*) ppm. ^{13}C NMR (75.5 MHz, DMSO-d$_6$): δ_C = 21.9 (2C, *C*H$_2$), 51.5 (t,$^1J_{CN}$ = 4.0 Hz, 2C, N*Me*$_2$), 65.3 (t, $^1J_{CN}$ = 3.1 Hz, 2C, N*C*H$_2$), 86.0 (2C, Cp), 96.7 (2C, Cp), 98.2 (1C, Cp), 166.3 (1C, *C*OO-AlMe$_2$), 233.9 (3C, Mo(*C*O)$_3$) ppm; elemental analysis calcd (%) for $C_{24}H_{37}AlMoN_2O_7$ (588.51 g mol^{-1}): C, 48.98; H, 6.34; N, 4.76; found: C 48.64, H 6.37, N 4.71. Note: The ^{13}C-NMR signal of the $AlMe_2$ groups could not be detected.

Synthesis of [DMP]$_2$[$C_5H_3(COS)_2AlMe_2$] (6). [DMP]$_2$[$C_5H_3(COS)_2H$] (100 mg, 0.23 mmol, 1.0 equiv.) was suspended in toluene (10 mL) and $AlMe_3$ (1.03 M in toluene, 0.23 mL, 0.23 mmol, 1.0 equiv.) was added at -20 °C. After 2h stirring at r.t. the methane evolution stopped, the solvent was removed *in vacuo*. The yellow residue was suspended and sonicated in n-pentane (20 mL) before the solvent was removed by syringe and the solid was finally dried in vacuo again. [DMP]$_2$[$C_5H_3(COS)_2AlMe_2$] was obtained as yellow solid in quantitative yield. IR (neat): $\tilde{\nu}_{max}$= 3004 (w), 2922 (w) 2885 (w), 1625 (w), 1467 (m), 1382 (m), 1250 (m), 1295 (m), 1218 (s), 1172 (m), 1063 (w), 1023 (s) 977 (w) 933 (w), 827 (vs), 742 (m), 704 (m), 661 (s), 580 (w), 511 (w), 430 (vw) cm^{-1}; ^{1}H NMR (300.1 MHz, DMSO-d$_6$): δ_H = -1.14 (s, 3H, Al*Me*$_2$), 2.07 (br s, 4H, C*H*$_2$), 3.05 (s, 6H, N*Me*$_2$), 3.41 (br s, 4H, NC*H*$_2$), 5.45 (t, $^3J_{HH}$= 2.9 Hz, 1H, Cp*H*), 7.11 (d, $^3J_{HH}$ = 2.9 Hz, 2H, Cp*H*) ppm. ^{13}C NMR (75.5 MHz, DMSO-d$_6$): δ_C = -9.40 (2C, Al*Me*$_2$), 21.3 (2C, *C*H$_2$), 50.9 (t, $^1J_{CN}$= 4.0 Hz, 2C, N*Me*$_2$), 64.7 (t, $^1J_{CN}$ = 3.1 Hz, 2C, N*C*H$_2$), 111.3 (2C, Cp), 126.7 (2C, Cp), 132.5 (1C, Cp), 209.8 (1C, *C*SO-

$AlMe_2$) ppm; elemental analysis calcd (%) for $C_{21}H_{37}AlN_2O_2S_2$ (440.64 g mol^{-1}): C 57.24; H 8.46; N 6.36; S 14.55; found: C 56.94, H 8.16, N 6.25.

Synthesis of [η^6-(p-cymene)Ru-η^5-($C_5H_3(CO_2)_2H$] (7). $[NMe_4]_2[C_5H_3(CO_2)_2H]$ was synthesized by reaction of $[NMe_4][OCO_2Me]$ with CpH in the same way described for $[DMP]_2[C_5H_3(CO_2)_2H]$. $[NMe_4]_2[C_5H_3(CO_2)_2H]$ (400mg, 1.33 mmol, 1.0 equiv.) and [Ru(p-cymene)$_2Cl_2$)]$_2$ (651 mg, 0.67 mmol, 0.5 equiv.) were suspended in MeCN (20 mL) and heated to 60 °C for 2h. After cooling to r.t. the solvent was removed *in vacuo*. The residue was threated with 1,2-dichloroethane (15 mL) and insoluble $[NMe_4]Cl$ was removed by filtration through Celite®. The filtrate was dried *in vacuo* to obtain a colourless crystalline solid that was washed with Et_2O (20 mL) before it was finally dried *in vacuo* again. [η^6-(p-cymene)Ru-η^5-($C_5H_3(CO_2)_2H$] was obtained as colourless solid in a yield of 469 mg (1.21 mmol, 91%). Colourless blocks of X-ray diffraction quality were obtained by layering a saturated solution of (**7**) in MeCN with Et_2O at r.t. IR (neat): $\tilde{\nu}_{max}$= 3067 (w), 2972 (w), 1593 (s), 1475 (m), 1355 (m), 1327 (m), 1194 (m), 1089 (m), 1013 (m), 938 (w), 913 (w), 876 (m), 783 (w), 683 (m), 624 (m), 575 (m), 535 (m), 446 (m) cm^{-1}; ^{1}H NMR (300.1 MHz, DMSO-d$_6$): δ_H = 1.19 (s, 6H, CH(C*H*$_3$)$_2$), 2.14 (s, 3H, Me), 2.66 (m, 1H, C*H*(CH$_3$)$_2$), 5.49 (t, $^3J_{HH}$ = 2.4 Hz, 2H, Cp*H*), 5.73 (d, $^3J_{HH}$ = 2.4 Hz, 2H, Cp*H*), 6.11 (d, $^3J_{HH}$ = 6.6 Hz, 2H, $H_{arom.}$), 6.23 (d, $^3J_{HH}$ = 6.6 Hz, 2H, $H_{arom.}$), 20.04 (1H, COO*H*) ppm; ^{13}C NMR (75.5 MHz, DMSO-d$_6$): δ_C = 17.3 (1C, Me), 22.6 (2C, CH(*C*H$_3$)$_2$), 30.9 (1C, *C*H(CH$_3$)$_2$), 80.7 ($C_{arom.}$), 84.5 ($C_{arom.}$), 86.6 ($C_{arom.}$), 87.8 ($C_{arom.}$), 87.9 (2C, Cp), 101.4 (2C, Cp), 112.2 (1C, Cp), 164.9 (1C, *C*OOH) ppm; elemental analysis calcd (%) for $C_{17}H_{18}O_4Ru$ (387.40 g mol^{-1}): C 52.71; H 4.68; found: C 52.41; H 4.72.

Synthesis of [η^6-(p-cymene)Ru-η^5-($C_5H_3(CO_2)_2AlMe_2$] (8). [η^6-(p-cymene)Ru-η^5-($C_5H_3(CO_2)_2H$] (100 mg, 0.26 mmol, 1.0 equiv.) was suspended in toluene (10 mL) and $AlMe_3$ (1.03 M in toluene, 0.26 mL, 0.26 mmol, 1.0 equiv.) was added at -20 °C. After 2h stirring at r.t. the methane evolution stopped and the solvent was removed *in vacuo*. The yellow residue was suspended and sonicated in pentane (20 mL) before the solvent was removed by syringe and the solid was finally dried *in vacuo* again. [η^6-(p-cymene)Ru-η^5-($C_5H_3(CO_2)_2AlMe_2$] was obtained as yellow solid in quantitative yield. IR (neat): $\tilde{\nu}_{max}$= 3066 (w), 2925 (w), 1633 (s), 1602 (s), 1477 (m), 1438 (m), 1373 (m), 1327 (m), 1180 (w), 1080 (m), 1036 (m), 1009 (w), 927 (w), 889 (m), 861 (m), 822 (w), 783 (m), 667 (m), 579 (w), 528 (m), 449 (m) cm^{-1}; ^{1}H NMR (300.1 MHz, DMSO-d$_6$): δ_H = -1.07 (br s, 6H, AlMe$_2$), 1.20 (s, 6H, CH(C*H*$_3$)$_2$), 2.17 (s, 3H, Me), 2.69 (m, 1H, C*H*(CH$_3$)$_2$), 5.58 (t, $^3J_{HH}$ = 2.4 Hz, 2H, Cp*H*),

5.80 (d, $^3J_{HH}$ = 2.4 Hz, 2H, Cp*H*), 6.12 (d, $^3J_{HH}$ = 6.6 Hz, 2H, $H_{arom.}$), 6.22 (d, $^3J_{HH}$ = 6.6 Hz, 2H, $H_{arom.}$) ppm; ^{13}C NMR (75.5 MHz, DMSO-d$_6$): δ_C = -9.7 (Al*Me*$_2$) 17.6 (1C, Me), 22.5 (2C, CH(*C*H$_3$)$_2$), 30.8 (1C, *C*H(CH$_3$)$_2$), 81.0 ($C_{arom.}$), 86.1 ($C_{arom.}$), 86.3 ($C_{arom.}$), 88.1 ($C_{arom.}$), 88.2 (2C, Cp), 101.9 (2C, Cp), 112.5 (1C, Cp), 163.1 (1C, *C*OO$^-$) ppm; elemental analysis calcd (%) for $C_{19}H_{23}AlO_4Ru$ (443.44 g mol^{-1}): C 51.46; H 5.23; found: C 51.34; H 5.35.

Synthesis of [DMP]$_5$[La(κ^2-O$_2$C-C$_5$H$_4$)$_4$] (9). [DMP]$_2$[C$_5$H$_3$(CO$_2$)$_2$H] (100 mg, 0.28 mmol, 1.5 equiv.) and LaCp$_3$ (63 mg, 0.19 mmol, 1.0 equiv.) were slightly dissolved in MeCN (10 mL) and the suspension was heated to 50 °C for 2h. After cooling down to r.t., the excess of [DMP]$_2$[C$_5$H$_3$(CO$_2$)$_2$H] was removed by syringe filtration. The solution was concentrated to 3-4 mL and cooled to 0 °C for some days. The formed crystals were isolated by removing the solvent by syringe and washing the crystals with Et$_2$O (10 mL). The crystals were dried *in vacuo*. [DMP]$_5$[La(C$_5$H$_4$CO$_2$)$_4$] was obtained as purple-brown crystals in a yield of (56 mg, 0.13 mmol, 69% (based on La). IR (neat): $\tilde{\nu}_{max}$= 3015 (w), 2963 (w), 2889 (w), 2684 (w), 1634 (s), 1464 (m), 1387 (w), 1345 (m), 1207 (w), 1181 (m), 1026 (m), 1001 (m), 934 (m), 915 (m), 853 (w), 783 (w), 709 (m), 616 (m), 582 (m), 470 (m) cm^{-1}; ^{1}H NMR (300.1 MHz, DMSO-d$_6$): δ_H = 1.93 (br s, 20H, C*H*$_2$), 2.84 (s, 30H, N*Me*$_2$), 3.22 (br s, 20H, NC*H*$_2$), 5.40 (t, $^3J_{HH}$ = 2.7 Hz, 2H, Cp*H*), 5.48 (t, $^3J_{HH}$ = 2.7 Hz, 2H, Cp*H*) ppm. ^{13}C NMR (75.5 MHz, DMSO-d$_6$): δ_C = 21.4 (10C, *C*H$_2$), 50.9 (t, $^1J_{CN}$= 4.0 Hz, 10C, N*Me*$_2$), 64.6 (t, $^1J_{CN}$= 3.1 Hz, 10C, N*C*H$_2$), 103.0 (8C, Cp), 105.7 (4C, Cp), 115.2 (8C, Cp); elemental analysis calcd (%) for $C_{54}H_{86}LaN_5O_8$ (1072.21 g mol^{-1}): C 60.49; H 8.08; N 6.53; found: C 60.12, H 7.92, N 6.69. Note: The ^{13}C-NMR signal of the carboxyl groups could not be detected.**Additional syntheses**

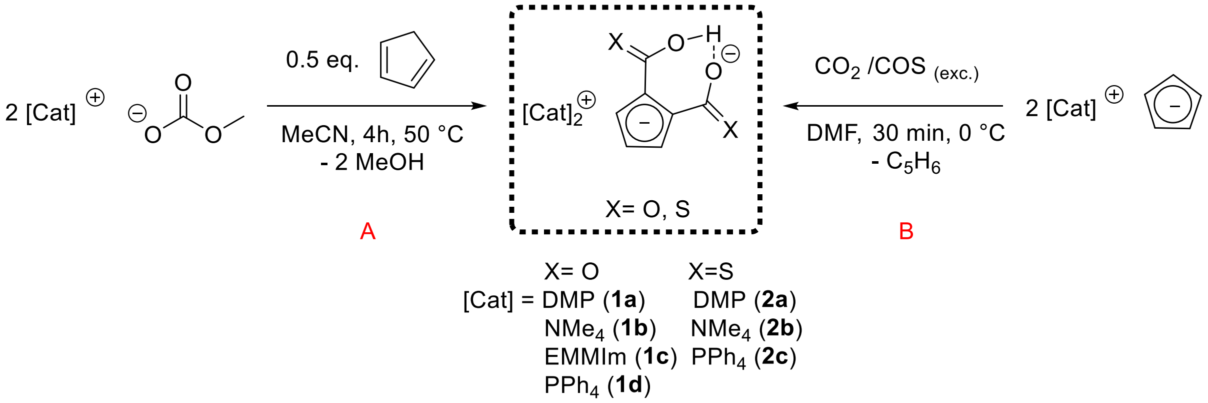

Scheme S1: Synthesis of [NMe$_4$]$_2$[C$_5$H$_3$(CO$_2$)$_2$H] by Method A or B.

Synthesis of [NMe$_4$]$_2$[C$_5$H$_3$(CO$_2$)$_2$H] (1b)

The reaction was performed according to Procedure A by reacting $NMe_4[OCO_2Me]$ with an excess of CpH. Yield: 78%, colourless solid. **^{1}H-NMR** (300.1 MHz, DMSO-d$_6$): δ_H = 3.07 (s, 24H, Me), 5.30 (t, $^3J_{HH}$ = 3.2 Hz, 1H, H-4), 6.06 (d, $^3J_{HH}$ = 3.3 Hz, 2H, H-3/5), 19.9 (bs, 1H, -COO*H*) ppm. **^{13}C-NMR** (75.5 MHz, DMSO-d$_6$): δ_C = 54.2 (t, $^1J_{CN}$= 3.9 Hz, Me), 105.7 (C-1/C-2), 115.3 (C-3/C-5), 170.8 (-COOH) ppm. **elemental analysis**: $C_{15}H_{28}N_2O_4$, *M* = 300.40 g·mol$^-$1, calc. C 59.98, H 9.40, N 9.33%; found C 59.20, H 9.46, N 9.87%.,

Synthesis of $[EMMIm]_2[C_5H_3(CO_2)_2H]$ (1c)

The reaction was performed according to Procedure A by reacting EMMIm$[OCO_2Me]$ with an excess of CpH. Yield: 72%, colourless solid.**^{1}H-NMR** (300.1 MHz, DMSO-d$_6$): δ_H = 1.32 (t, $^3J_{HH}$ = 7.2 Hz, 6H, H-7‘), 2.56 (s, 6H, H-9‘), 3.73 (s, 6H, H-8‘), 4.12 (q, $^3J_{HH}$ = 7.4 Hz, 4H, H-6‘), 5.27 (t, 1H, $^3J_{HH}$ = 3.2 Hz, H-4), 6.03 (d, 2H, $^3J_{HH}$ = 3.3 Hz, H-3/H-5), 7.61 (d, 2H, $^3J_{HH}$ = 1.9 Hz, H-4‘/5‘) 7.65 (d, 2H, $^3J_{HH}$ = 1.9 Hz, H-4‘/5‘), 19.9 (bs, 1H, -COO*H*) ppm. **^{13}C-NMR** (75.5 MHz, DMSO-d$_6$): δ_C = 8.9 (C-9’), 14.7 (C-7’), 34.5 (C-8’), 42.6 (C-6’), 105.6 (C-1/C-2), 115.0 (C-3/C-5), 116.9 (C-4), 120.3 (C-4‘/5’), 122.3 (C-4‘/5’), 143.9 (C-2’), 170.8 (-COOH) ppm. **elemental analysis**: $C_{21}H_{30}N_4O_4$, *M* = 402.50 g·mol^{-1}, calc. C 62.67, H 7.51, N 13.92%; found C 61.92, H 7.53, N 14.29%.

Synthesis of $[PPh_4]_2[C_5H_3(CO_2)_2H]$ (1d)

The reaction was performed according to Procedure B by reacting PPh_4[Cp] with CO_2. Yield: 67%, yellow solid.**^{1}H-NMR** (300.1 MHz, DMSO-*d*$_6$): δ_H =5.24 (t, $^3J_{HH}$ = 3.2 Hz,1H, H-4), 6.04 (d, $^3J_{HH}$ = 3.2 Hz, 2H, H-3/5) 7.70-7.98 (m, PPh_4) 19.94 (bs, 1H,-COO*H*) ppm. **^{13}C-NMR** (75.5 MHz, DMSO-*d*$_6$): δ_C = 105.6 (C-1/2), 115.2 (C-3/C-5), 116.9 (C-4), 117.7 (d, $^1J_{PC}$ = 89.2 Hz, C-1‘), 130.4 (d, $^2J_{PC}$ = 12.8 Hz, C-2‘), 134.5 (d, $^3J_{PC}$ = 10.5 Hz, C-3‘), 135.3 (d, $^4J_{PC}$ = 2.9 Hz, C-4‘), 170.9 (-COOH) ppm. **elemental analysis**: $C_{54}H_{44}O_4P_2$, *M* = 830.90 g·mol^{-1}, calc. C 79.50, H 5.34%; found C 79.29, H 5.27%.

Synthesis of $[NMe_4]_2[C_5H_3(COS)_2H]$ (2b)

The reaction was performed according to Procedure B by reacting NMe_4[Cp] with COS. Yield: 82%, colourless solid. **^{1}H-NMR** (300.1 MHz, DMSO-d$_6$): δ_H = 3.08 (s, 24H, Me), 5.44(t, $^3J_{HH}$ = 3.6 Hz, 1H, H-4), 6.86 (d, $^3J_{HH}$ = 3.6 Hz, 2H, H-3/5), 18.73 (bs, 1H, -COS*H*) ppm. **^{13}C-NMR** (75.5 MHz, DMSO-d$_6$): δ_C = 54.4 (t, $^1J_{CN}$= 4.0 Hz, Me), 110.7 (C-1/C-2), 126.5 (C-4), 127.9 (C3-C5), 206.8 (-COSH) ppm. **elemental analysis**: $C_{15}H_{28}N_2O_2S_2$, *M* = 332.52 g·mol^{-1}, calc. C 54.18, H 8.49, N 8.42%; found C 53.88, H 8.32, N 8.62%.

Synthesis of $[PPh_4]_2[C_5H_3(COS)_2H]$ (2c)

The reaction was performed according to Procedure B by reacting $PPh_4[Cp]$ with COS. Yield: 69%, yellow solid. **^{1}H-NMR** (300.1 MHz, DMSO-d_6): δ_H =5.44 (t, $^3J_{HH}$ = 3.5 Hz,1H, H-4), 6.89 (d, $^3J_{HH}$ = 3.5 Hz, 2H, H-3/5) 7.70-7.99 (m, PPh_4) 18.71 (bs, 1H,-COS*H*) ppm. **^{13}C-NMR** (75.5 MHz, DMSO-d_6): δ_C = 111.0 (C-1/2), 117.6 (d, $^1J_{PC}$ = 89.1 Hz, C-1‘), 126.3 (C-4), 128.4 (C-3/C-5), 130.4 (d, $^2J_{PC}$ = 12.8 Hz, C-2‘), 134.5 (d, $^3J_{PC}$ = 10.5 Hz, C-3‘), 135.3 (d, $^4J_{PC}$ = 2.9 Hz, C-4‘), 208-8 (-COSH) ppm. **elemental analysis**: $C_{55}H_{44}O_2P_2S_2$, M = 863.02 g·mol^{-1}, calc. C 76.55, H 5.14%; found C 76.13.92, H 5.02%.

2. NMR spectra

a. *[DMP]$_2$[C$_5$H$_3$(CO$_2$)$_2$H] (**1a**)*

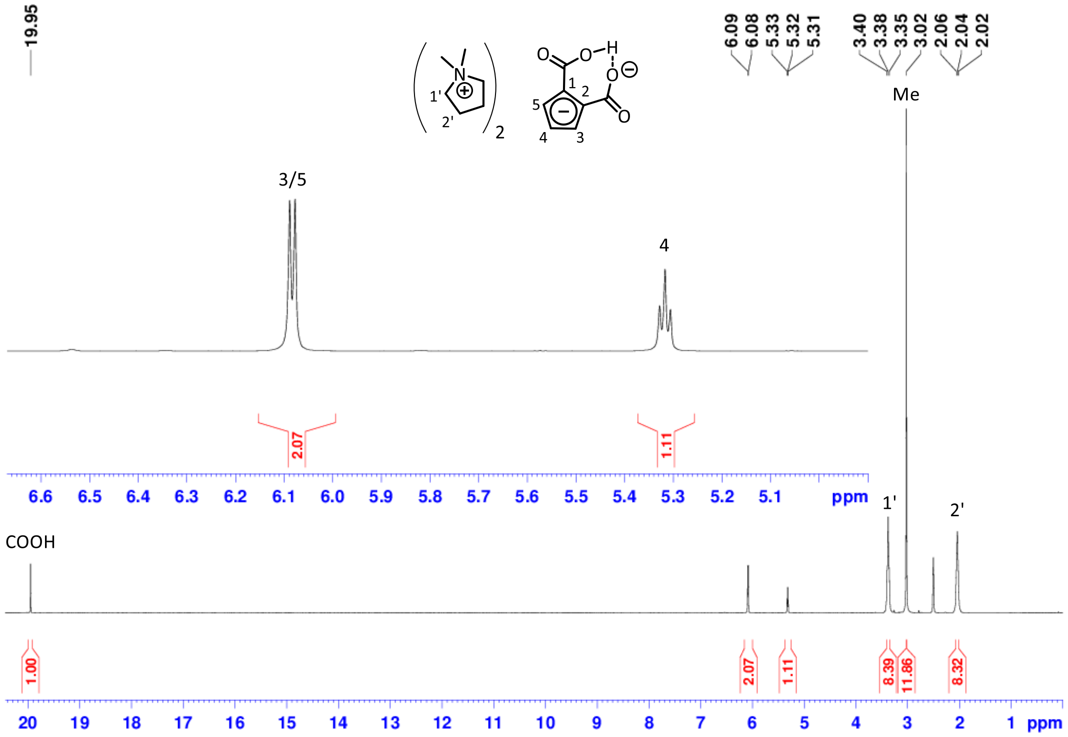

Figure S1. ^{1}H NMR spectrum (300.1 MHz, DMSO-d$_6$) of [DMP]$_2$[C$_5$H$_3$(CO$_2$)$_2$H] (**1a**).

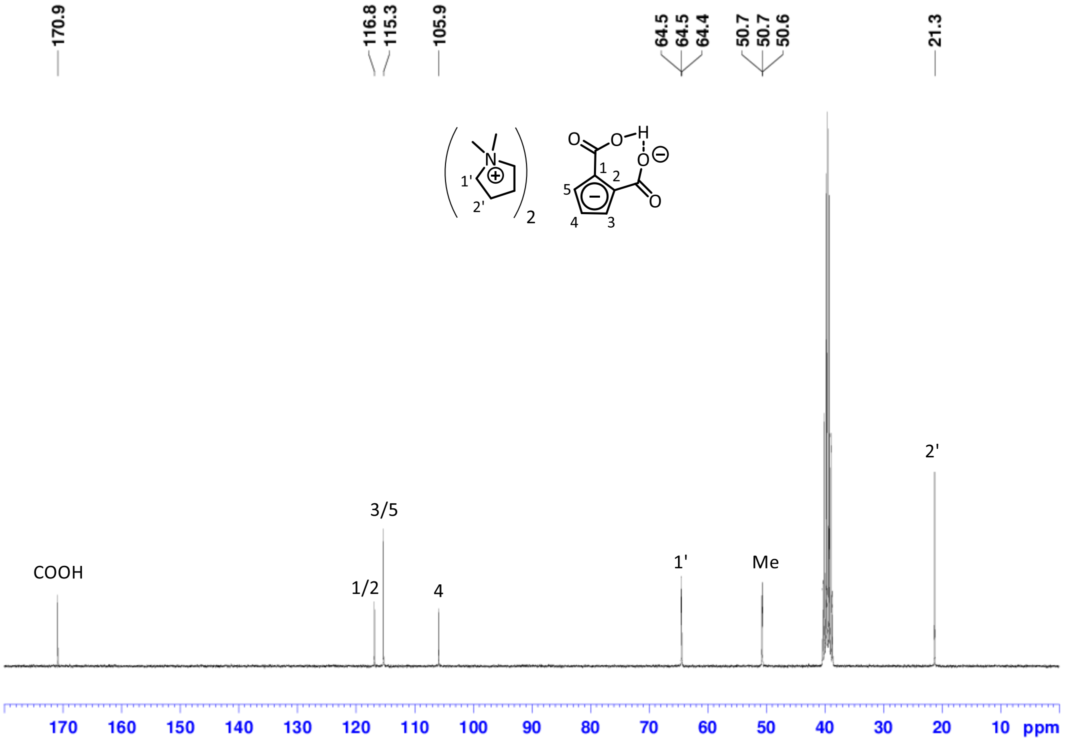

Figure S2. ^{13}C NMR spectrum (75.5 MHz, DMSO-d$_6$) of [DMP]$_2$[C$_5$H$_3$(CO$_2$)$_2$H] (**1a**).

*b. [NMe$_4$]$_2$[C$_5$H$_3$(CO$_2$)$_2$H] (**1b**)*

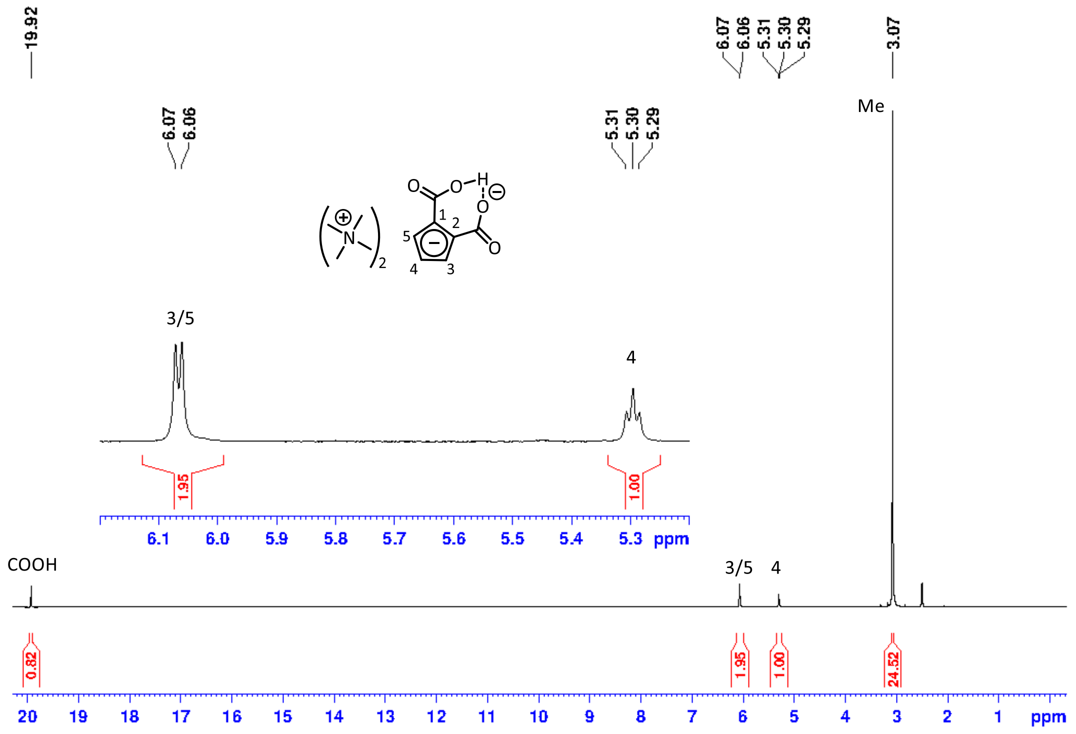

Figure S3. ^{1}H NMR spectrum (300.1 MHz, DMSO-d$_6$) of [NMe$_4$]$_2$[C$_5$H$_3$(CO$_2$)$_2$H] (**1b**).

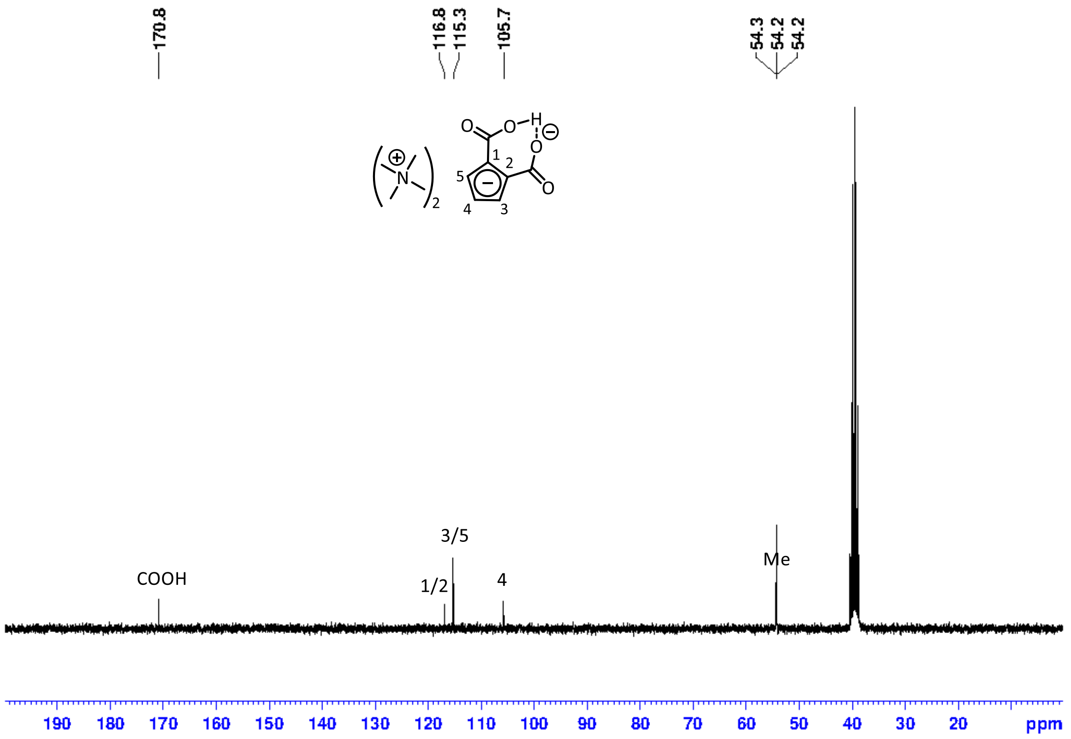

Figure S4. ^{13}C NMR spectrum (75.5 MHz, DMSO-d$_6$) of [NMe$_4$]$_2$[C$_5$H$_3$(CO$_2$)$_2$H] (**1b**).

c. *[EMMIm]$_2$[C$_5$H$_3$(CO$_2$)$_2$H] (**1c**)*

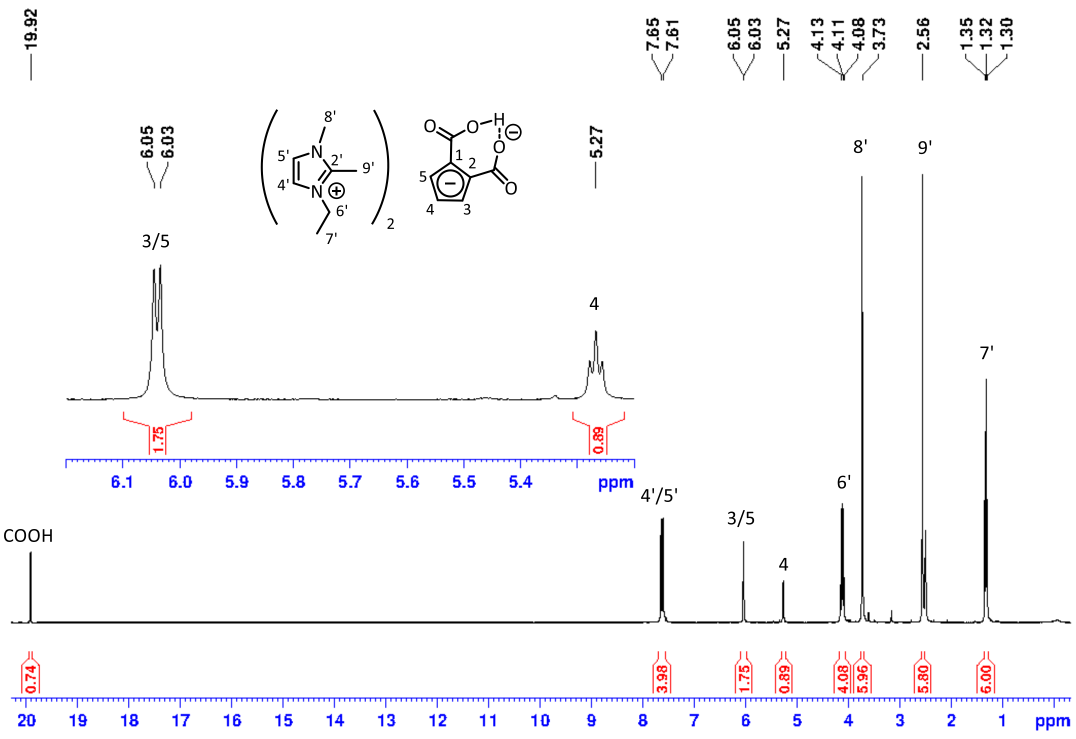

Figure S5. ^{1}H NMR spectrum (300.1 MHz, DMSO-d$_6$) of [EMMIm]$_2$[C$_5$H$_3$(CO$_2$)$_2$H] (**1c**).

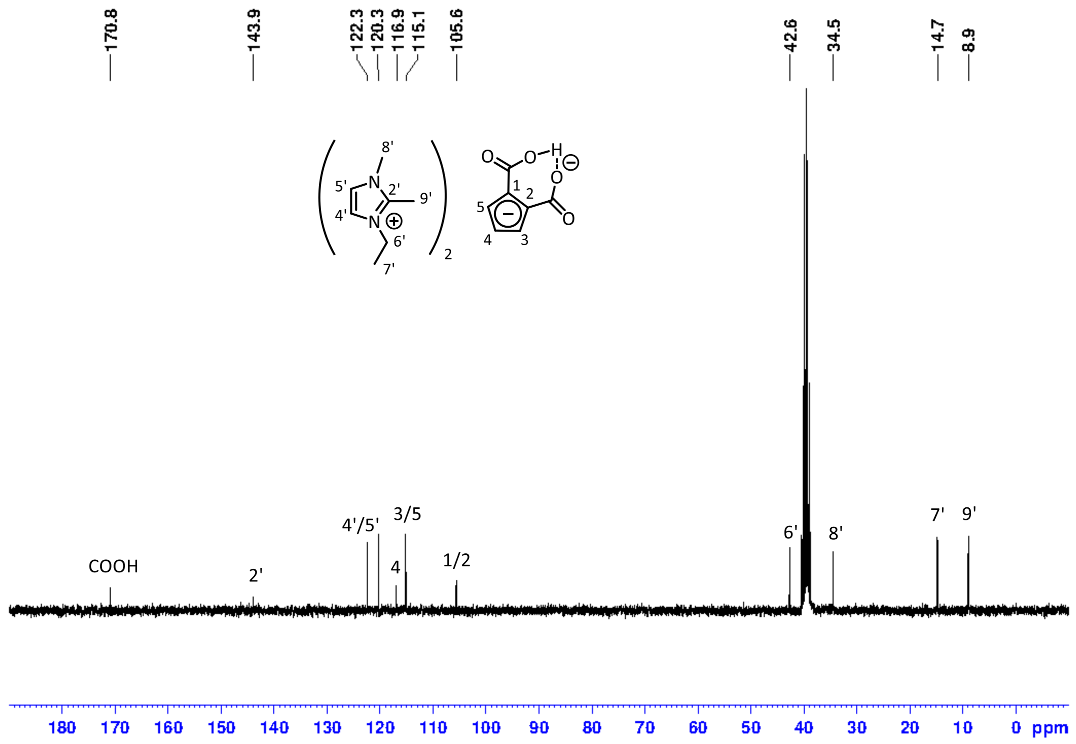

Figure S6. ^{13}C NMR spectrum (75.5 MHz, DMSO-d$_6$) of [EMMIm]$_2$[C$_5$H$_3$(CO$_2$)$_2$H] (**1c**).

d. [PPh4]2[C5H3(CO2)2H] (***1d***)

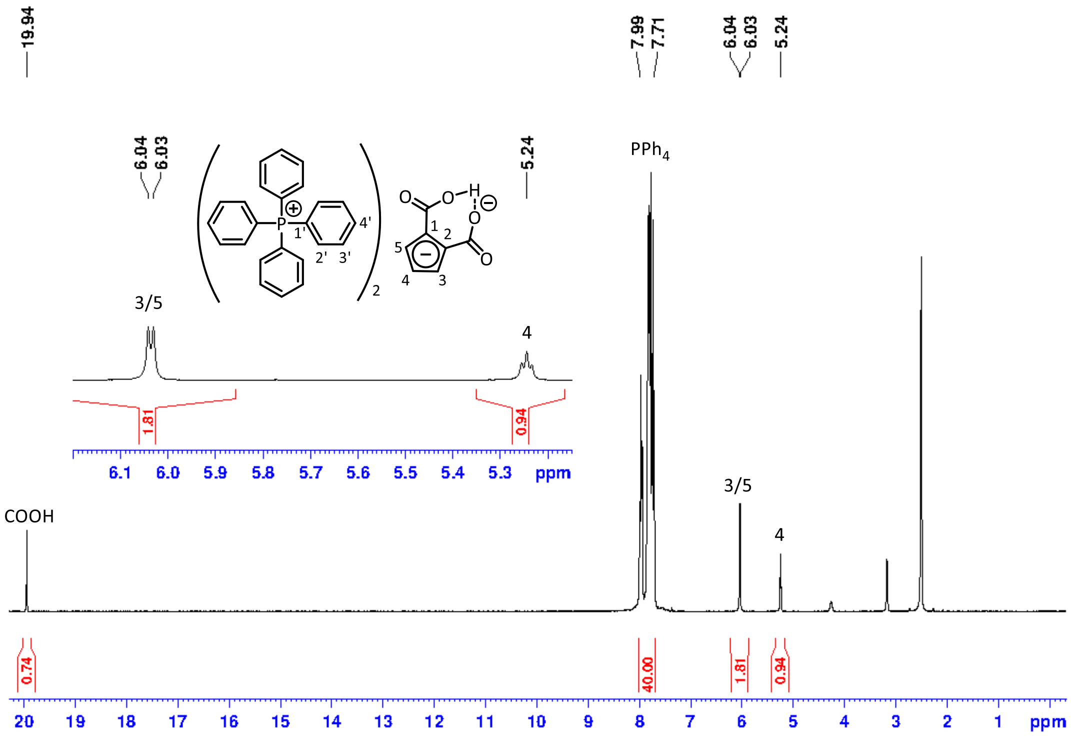

Figure S7. ^{1}H NMR spectrum (300.1 MHz, DMSO-d6) of $[PPh_4]_2[C_5H_3(CO_2)_2H]$ (**1d**).

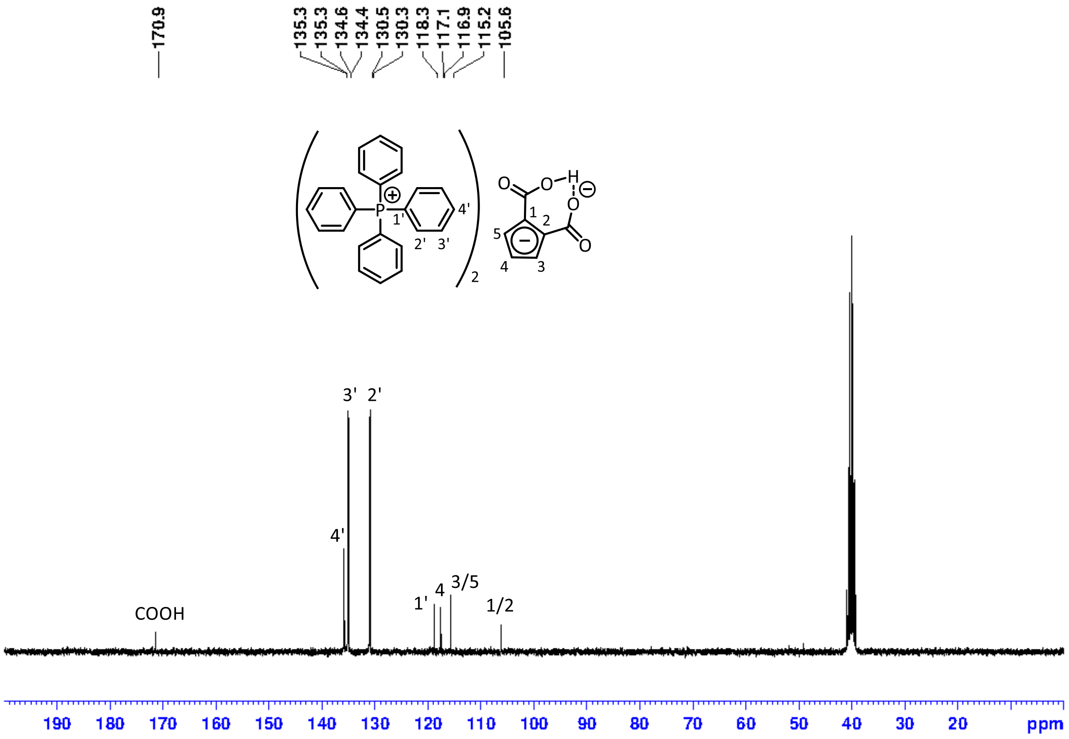

Figure S8. ^{13}C NMR spectrum (75.5 MHz, DMSO-d6) of $[PPh_4]_2[C_5H_3(CO_2)_2H]$ (**1d**).

*e. [DMP]$_2$[C$_5$H$_3$(COS)$_2$H] (**2a**)*

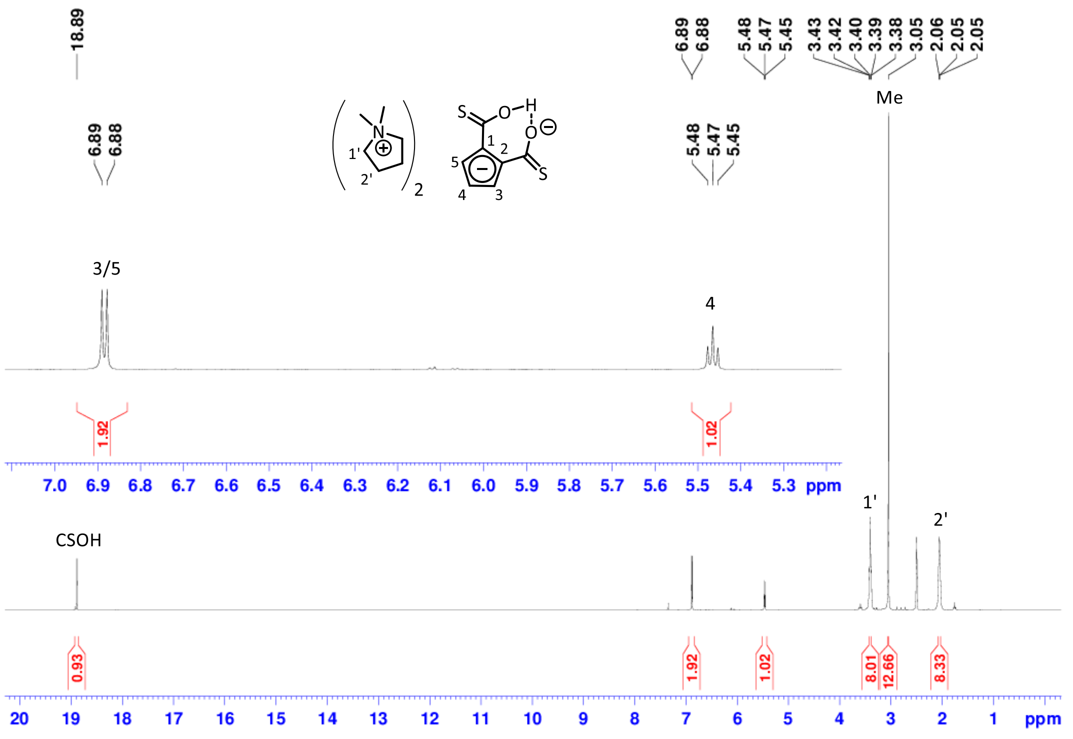

Figure S9. ^{1}H NMR spectrum (300.1 MHz, DMSO-d$_6$) of [DMP]$_2$[C$_5$H$_3$(COS)$_2$H] (**2a**).

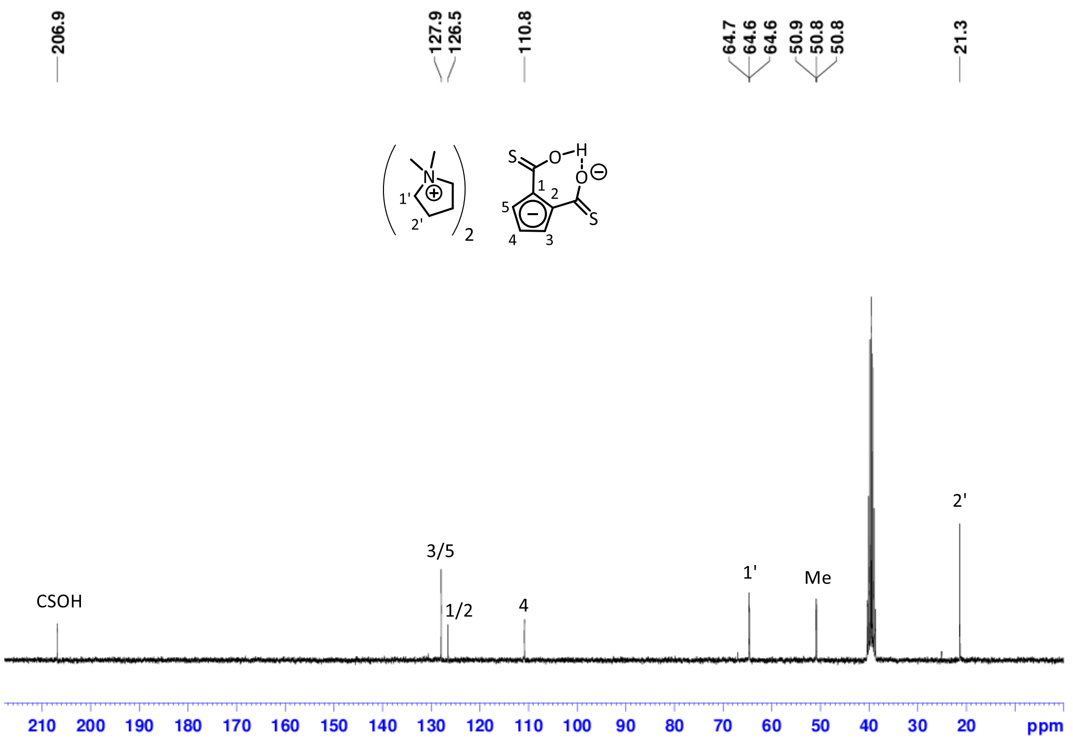

Figure S10. ^{13}C NMR spectrum (75.5 MHz, DMSO-d$_6$) of [DMP]$_2$[C$_5$H$_3$(COS)$_2$H] (**2a**).

f. *[NMe$_4$]$_2$[C$_5$H$_3$(COS)$_2$H]* ***(2b)***

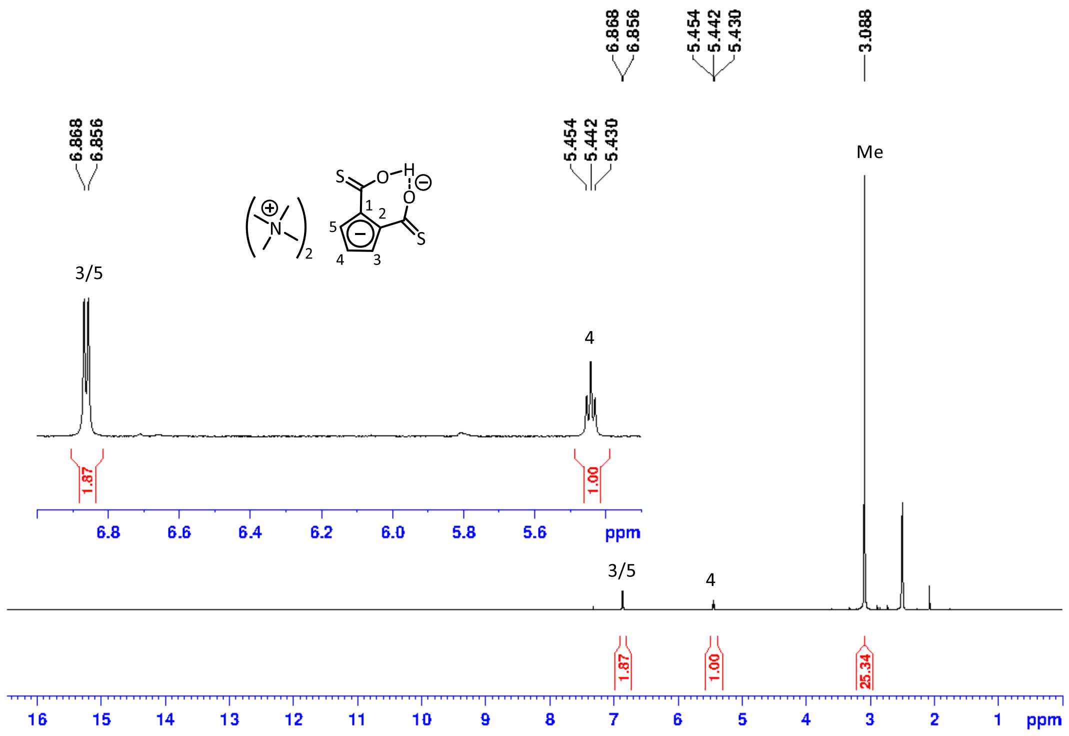

Figure S11. ^{1}H NMR spectrum (300.1 MHz, DMSO-d$_6$) of [NMe$_4$]$_2$[C$_5$H$_3$(COS)$_2$H] (**2b**).

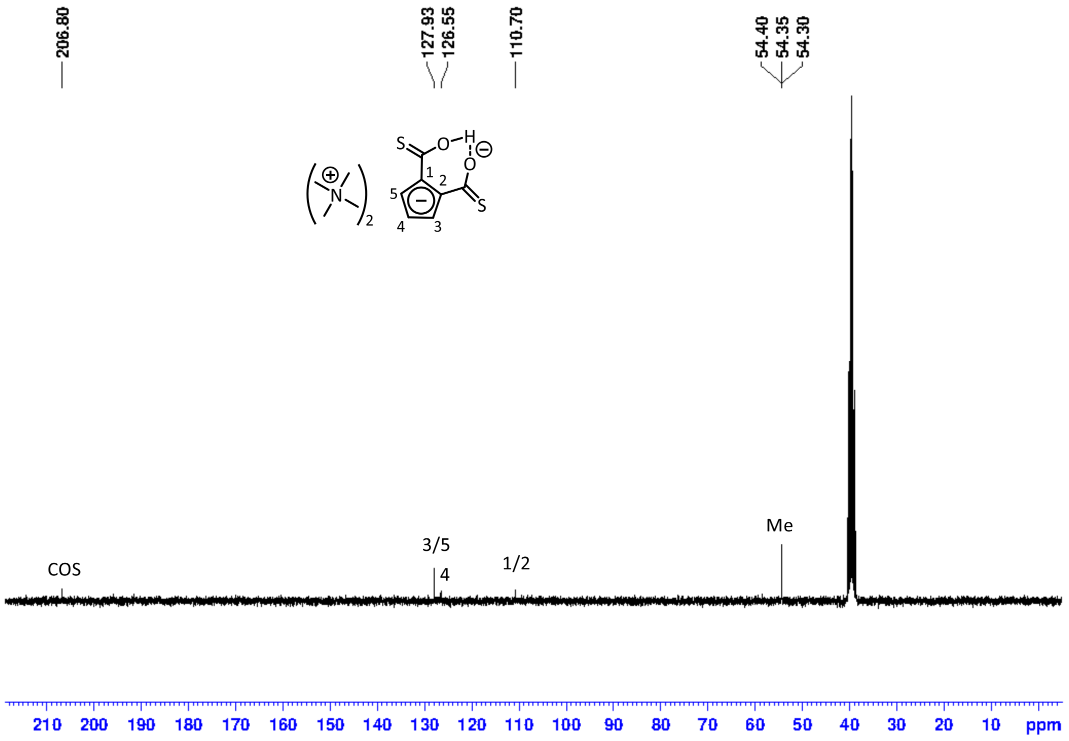

Figure S12. ^{13}C NMR spectrum (75.5 MHz, DMSO-d$_6$) of [NMe$_4$]$_2$[C$_5$H$_3$(CO$_2$)$_2$H] (**2b**).

*g. [PPh$_4$]$_2$[C$_5$H$_3$(COS)$_2$H] (**2c**)*

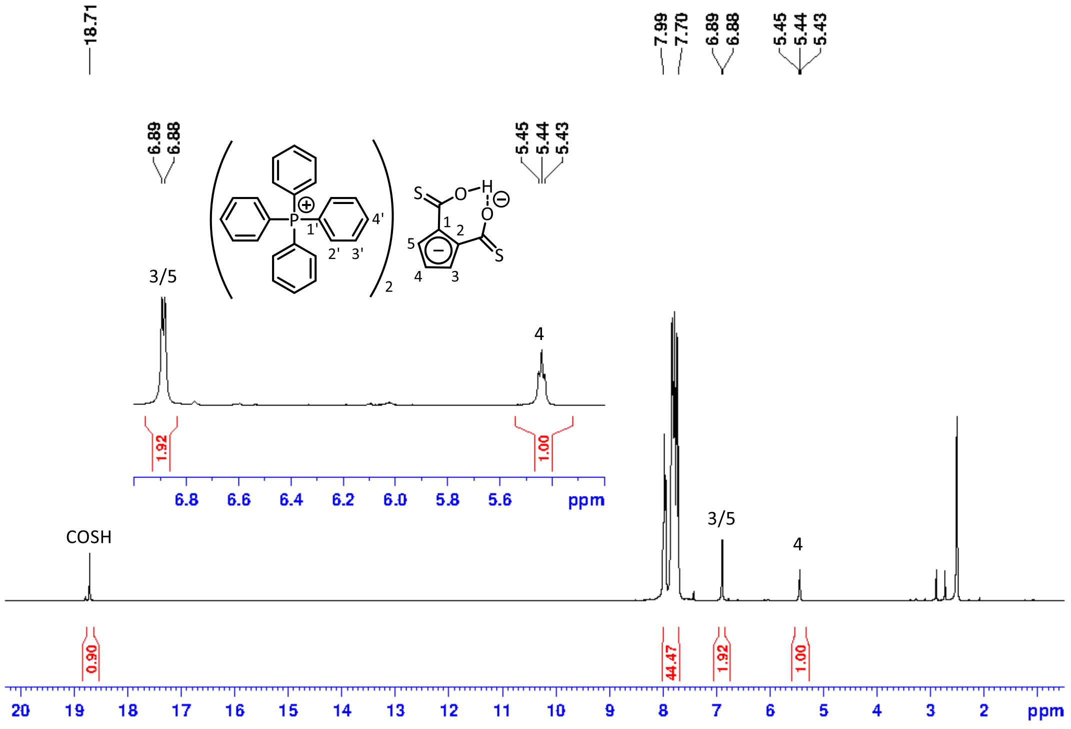

Figure S13. ^{1}H NMR spectrum (300.1 MHz, DMSO-d$_6$) of [PPh$_4$]$_2$[C$_5$H$_3$(COS)$_2$H] (**2c**).

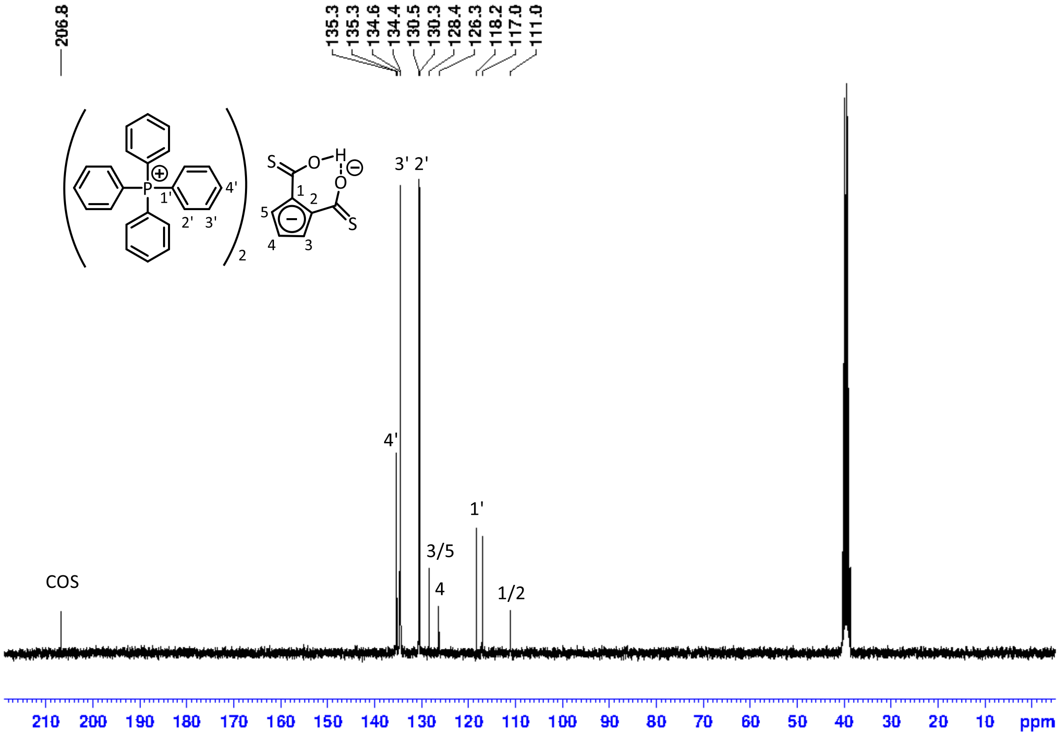

Figure S14. ^{13}C NMR spectrum (75.5 MHz, DMSO-d$_6$) of [PPh$_4$]$_2$[C$_5$H$_3$(CO$_2$)$_2$H] (**2c**).

h. *[DMP]$_2$[(η^5-$C_5H_3(CO_2)_2H$)Mo(CO)$_3$]* ***(3)***

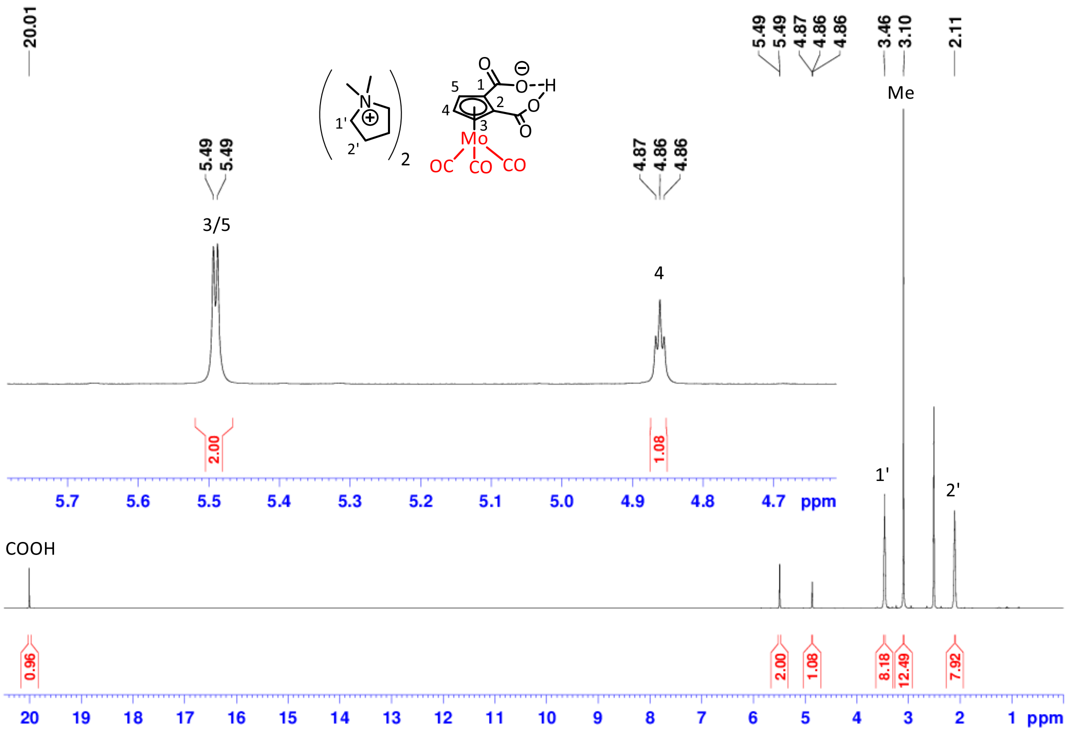

Figure S15. ^{1}H NMR spectrum (300.1 MHz, DMSO-d$_6$) of [DMP]$_2$[(η^5-$C_5H_3(CO_2)_2H$)Mo(CO)$_3$] (**3**).

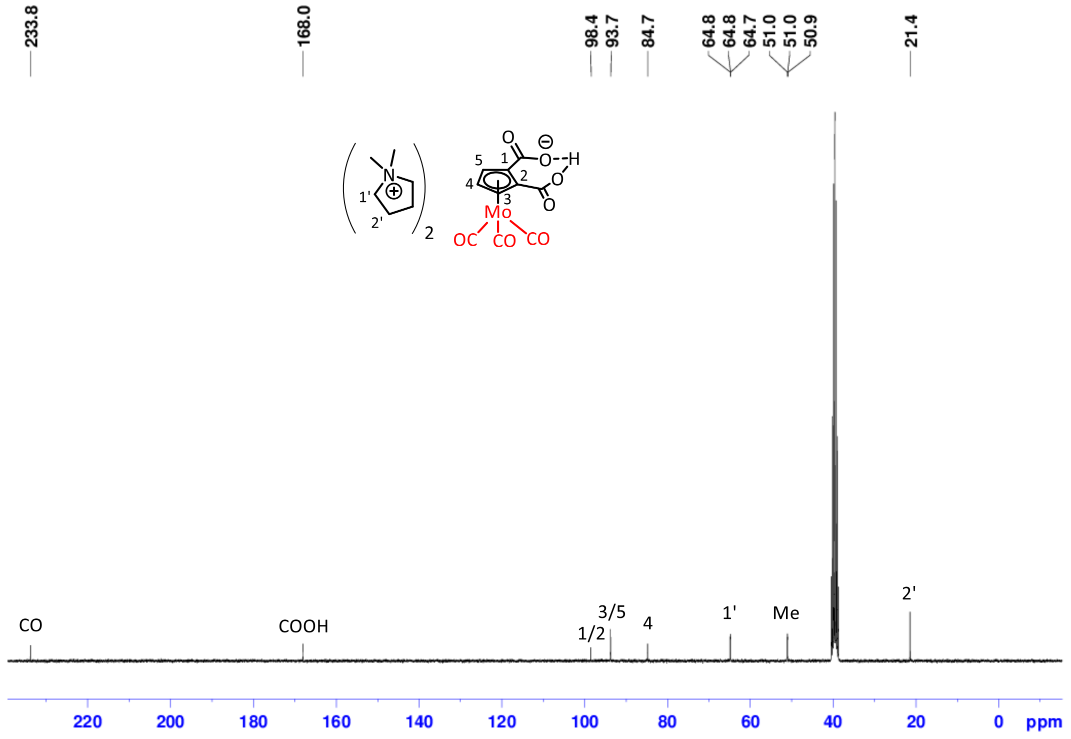

Figure S16. ^{13}C NMR spectrum (125.8 MHz, DMSO-d$_6$) of [DMP]$_2$[(η^5-$C_5H_3(CO_2)_2H$)Mo(CO)$_3$] (**3**).

i. *[DMP]$_2$[C$_5$H$_3$(CO$_2$)$_2$AlMe$_2$] (**4**)*

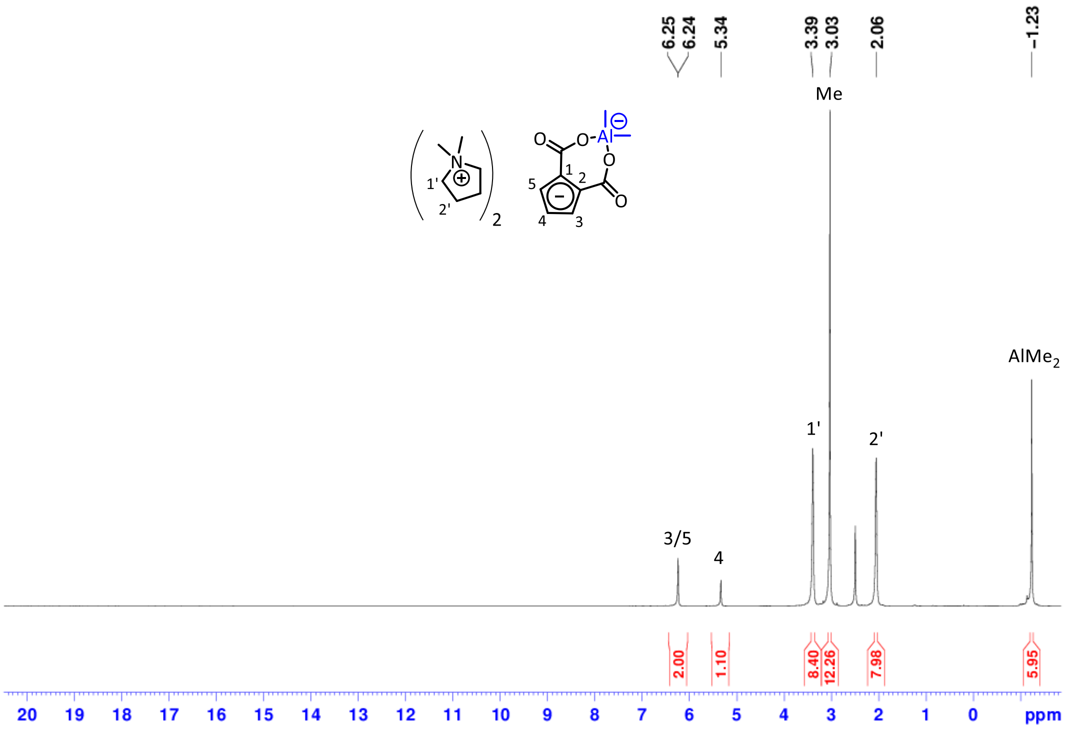

Figure S17. ^{1}H NMR spectrum (300.3 MHz, DMSO-d$_6$) of [DMP]$_2$[C$_5$H$_3$(CO$_2$)$_2$AlMe$_2$] (**4**).

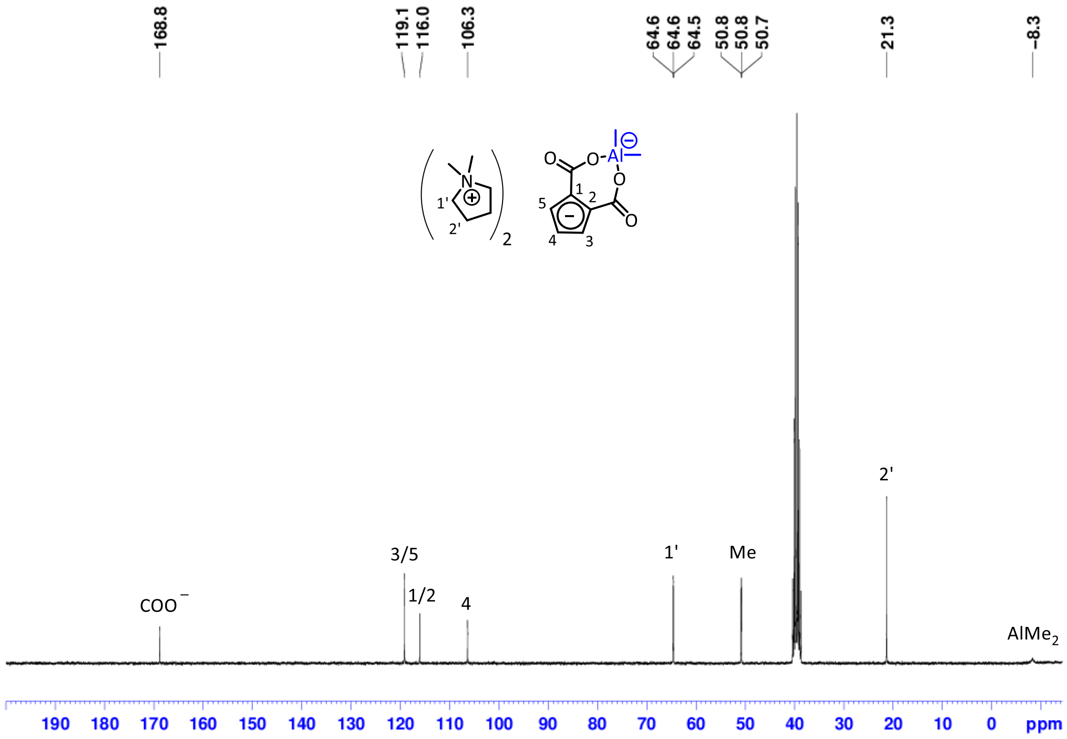

Figure S18. ^{13}C NMR spectrum (75.5 MHz, DMSO-d$_6$) of [DMP]$_2$[C$_5$H$_3$(CO$_2$)$_2$AlMe$_2$] (**4**).

*j. [DMP]$_2$[(η^5-$C_5H_3(CO_2)_2AlMe_2$)Mo(CO)$_3$] (**5**)*

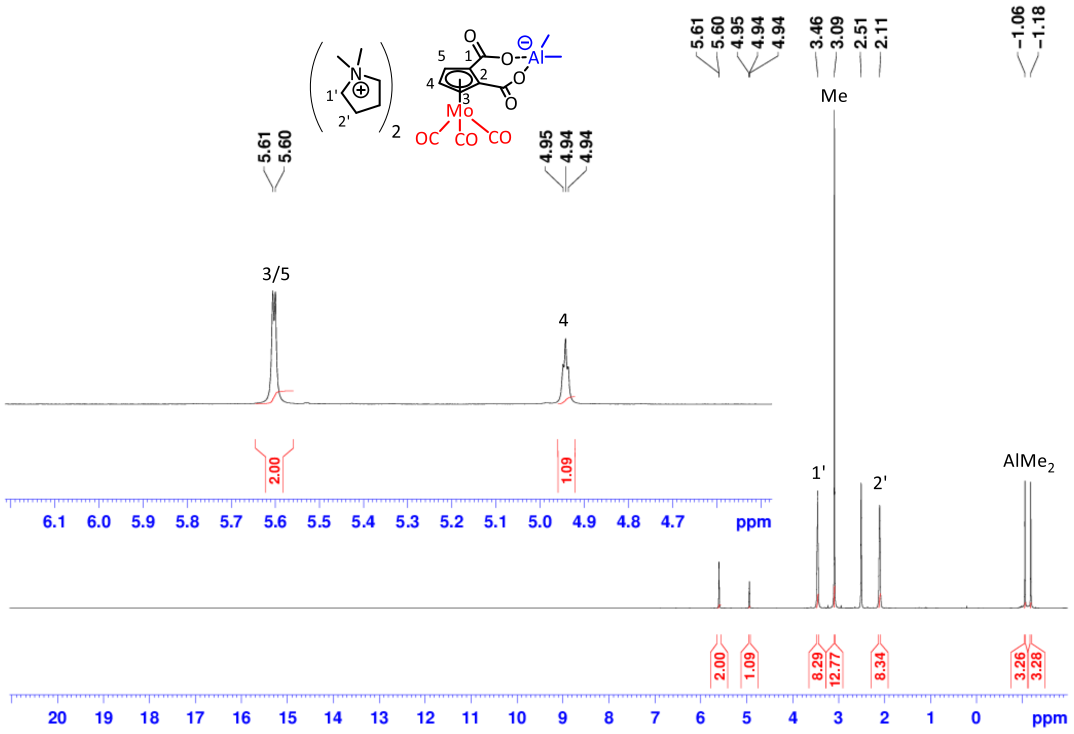

Figure S19. ^{1}H NMR spectrum (300.3 MHz, DMSO-d$_6$) of [DMP]$_2$[(η^5-$C_5H_3(CO_2)_2AlMe_2$)Mo(CO)$_3$] (**5**).

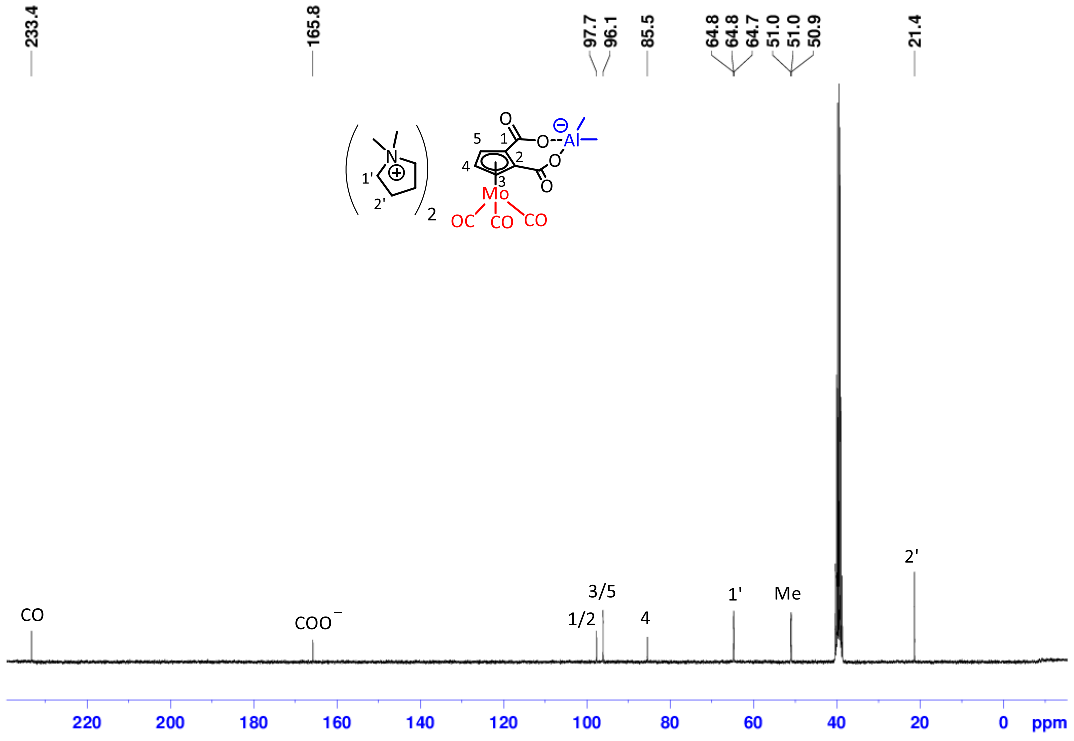

Figure S20. ^{13}C NMR spectrum (75.5 MHz, DMSO-d$_6$) of [DMP]$_2$[(η^5-$C_5H_3(CO_2)_2AlMe_2$)Mo(CO)$_3$] (**5**).

*k. [DMP]$_2$[$C_5H_3(COS)_2AlMe_2$] (**6**)*

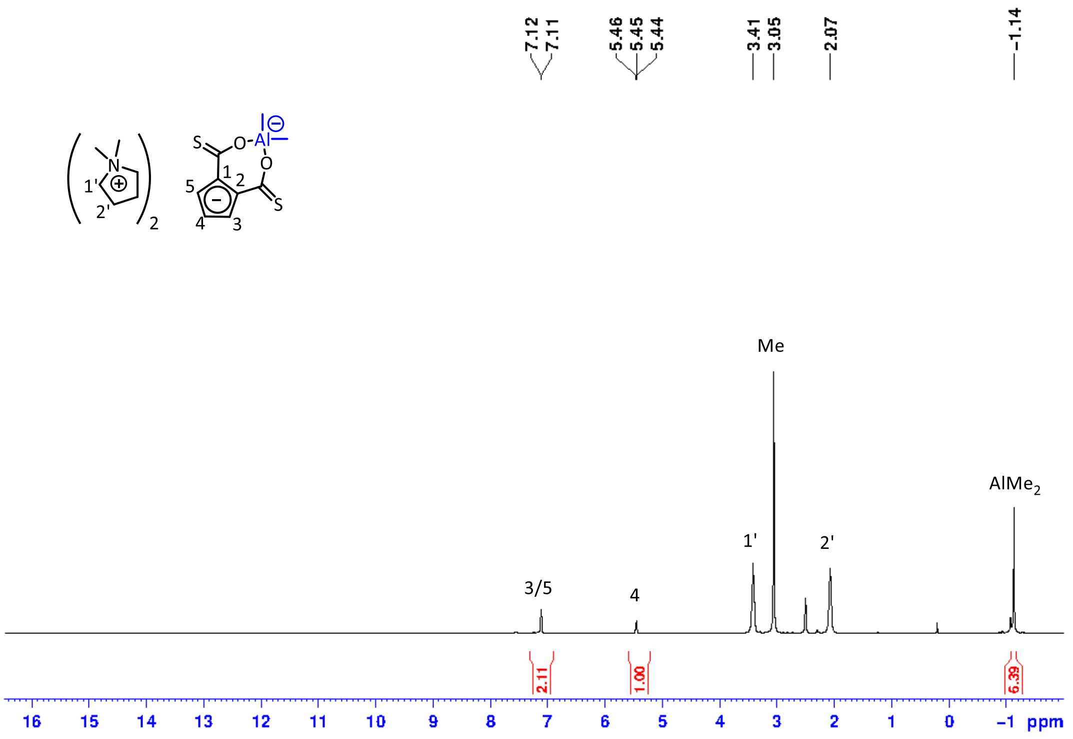

Figure S21. ^{1}H NMR spectrum (300.3 MHz, DMSO-d$_6$) of [DMP]$_2$[$C_5H_3(COS)_2AlMe_2$] (**6**).

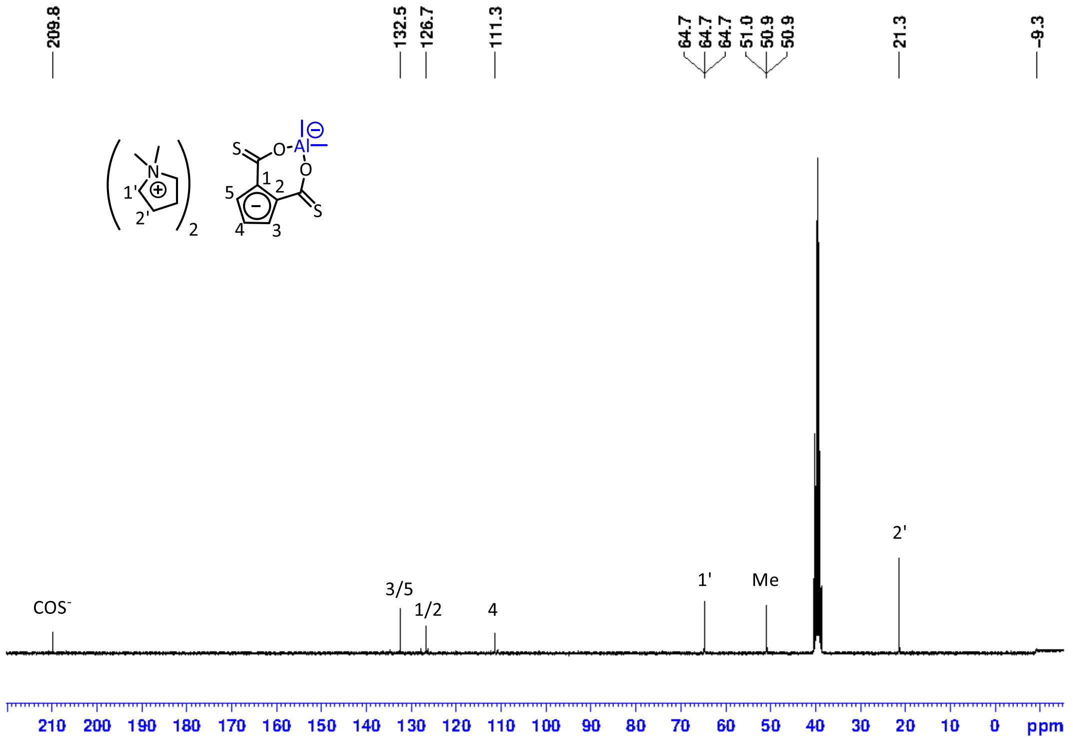

Figure S22. ^{13}C NMR spectrum (75.5 MHz, DMSO-d$_6$) of [DMP]$_2$[$C_5H_3(COS)_2AlMe_2$] (**6**).

*l. [η^6-p-cymene-Ru-η^5-$C_5H_3(CO_2)_2H$] (**7**)*

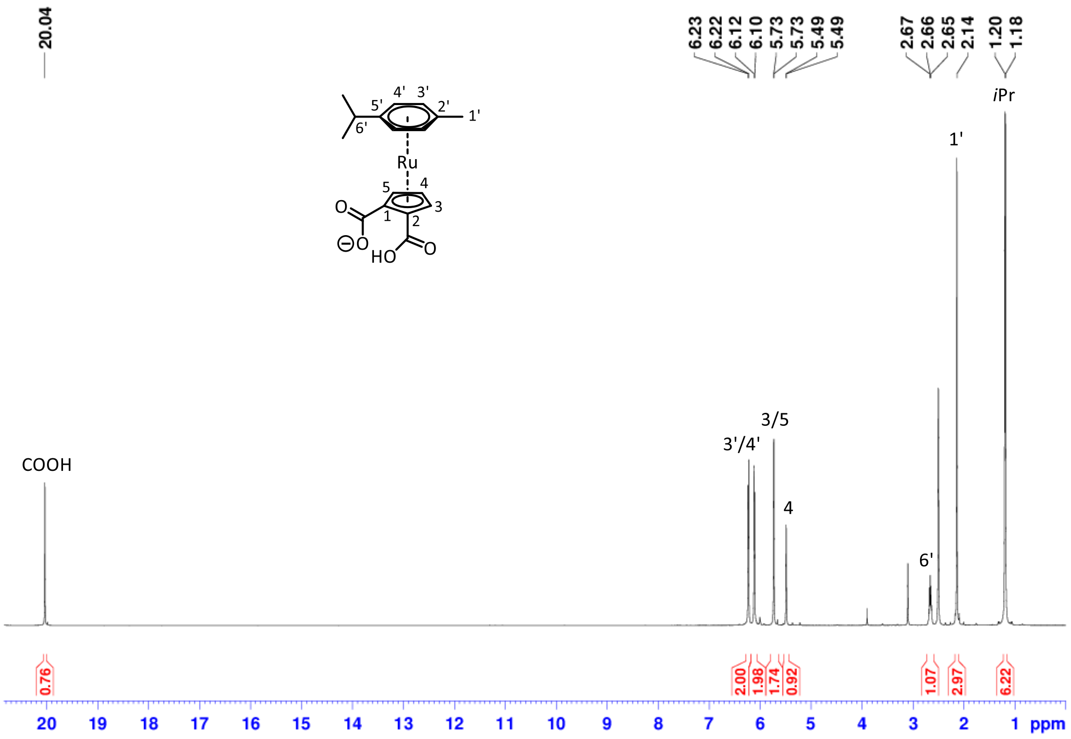

Figure S23. ^{1}H NMR spectrum (300.3 MHz, DMSO-d_6) of [η^6-p-cymene-Ru-η^5-$C_5H_3(CO_2)_2H$] (**7**).

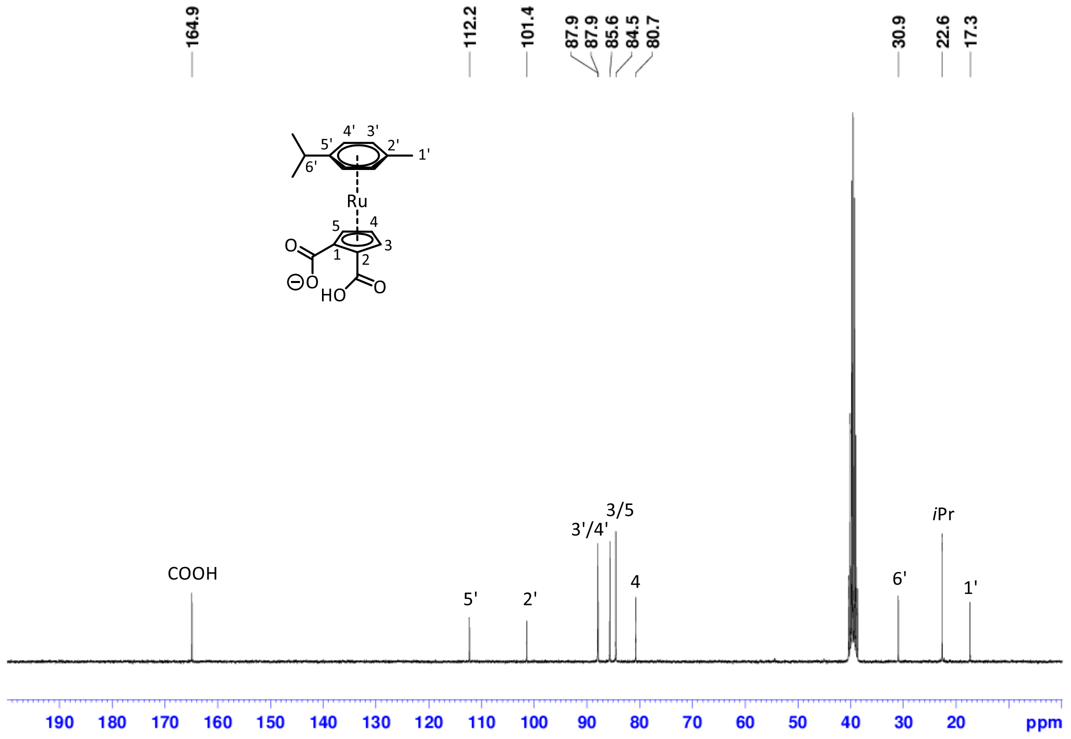

Figure S24. ^{13}C NMR spectrum (75.5 MHz, DMSO-d_6) of [η^6-p-cymene-Ru- η^5-$C_5H_3(CO_2)_2H$] (**7**).

*m. [η^6-p-cymene-Ru-η^5-$C_5H_3(CO_2)_2AlMe_2$] (**8**)*

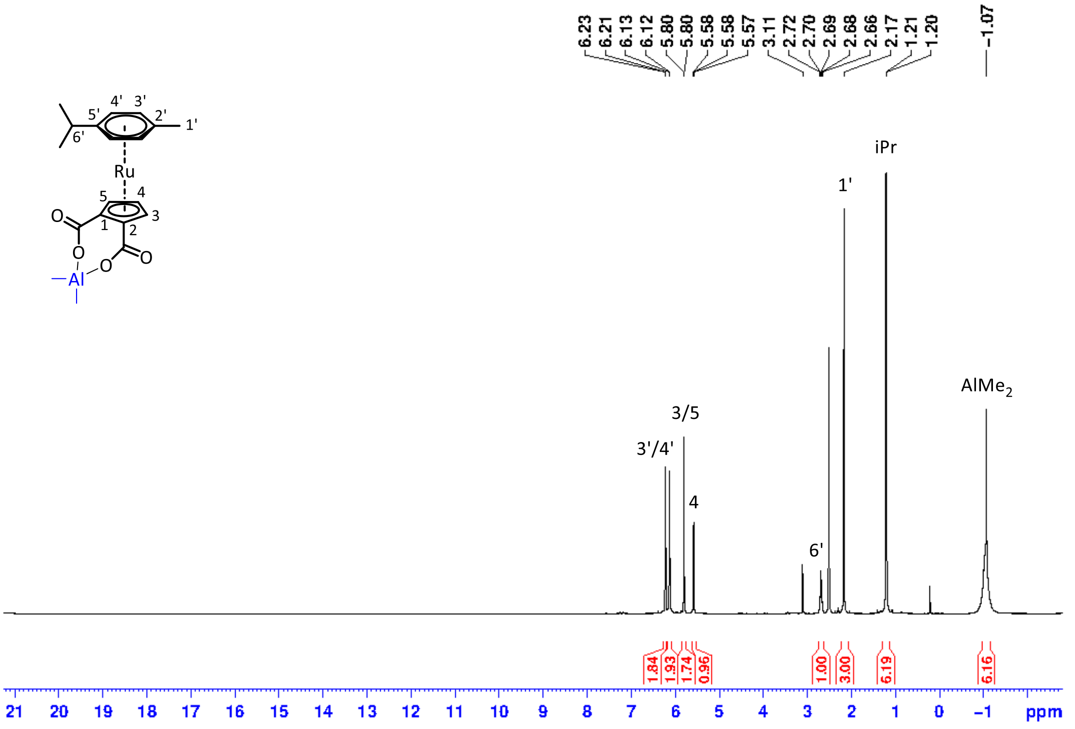

Figure S25. ^{1}H NMR spectrum (300.3 MHz, DMSO-d_6) of [η^6-p-cymene-Ru- η^5-$C_5H_3(CO_2)_2AlMe_2$] (**8**).

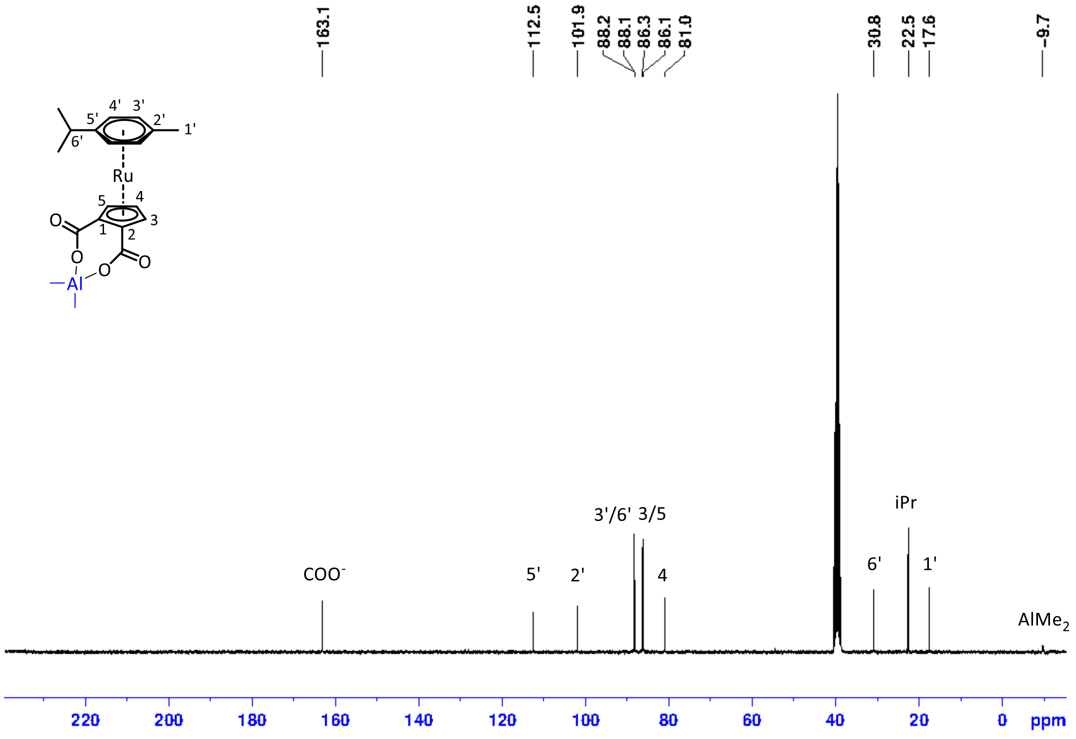

Figure S26. ^{13}C NMR spectrum (75.5 MHz DMSO-d_6) of [η^6-p-cymene-Ru- η^5-$C_5H_3(CO_2)_2AlMe_2$] (**8**).

n. *[DMO]$_5$[La($C_5H_4CO_2$)$_4$]* ***(9)***

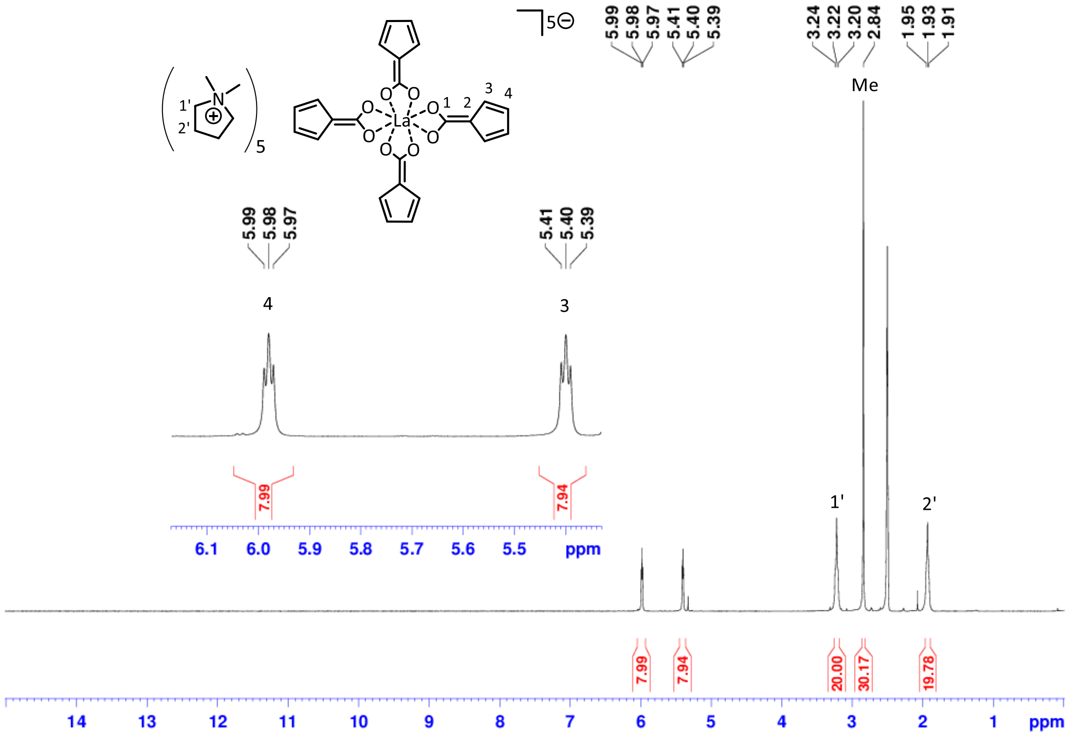

Figure S27. ^{1}H NMR spectrum (300.3 MHz, DMSO-d$_6$) of [DMP]$_5$[La($C_5H_4CO_2$)$_4$] (**9**).

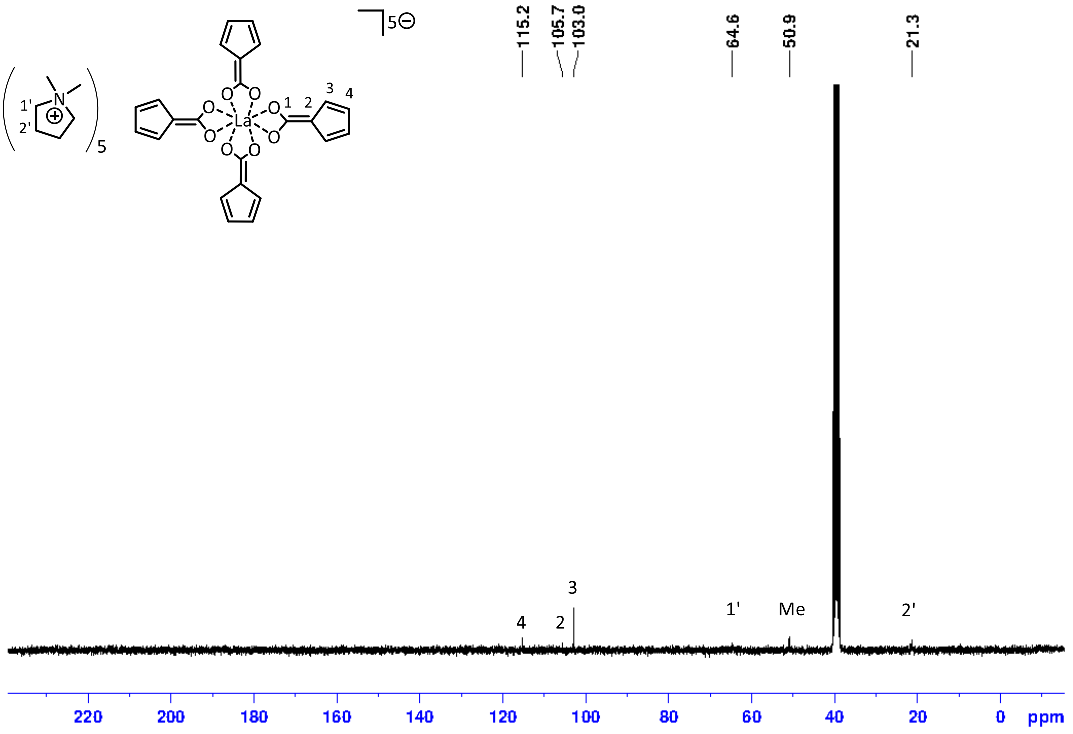

Figure S28. ^{13}C NMR spectrum (75.5 MHz, DMSO-d$_6$) of [DMP]$_5$[La($C_5H_4CO_2$)$_4$] (**9**).

3. Single crystal x-ray structures

The data collection for the single-crystal structure determination was performed on a Stoe Stadivari diffractometer or a Bruker D8 Quest diffractometer (Mo-K_α radiation: λ = 71.073 pm, Cu-K_α radiation: λ = 154.186 pm) by the X-ray service department of the Fachbereich Chemie, University of Marburg at 100K. Information concerning the used hardware, and software used for Data collection, cell refinement and data reduction as well as structure solution and refinement can be reviewed in the attached CIF-files. After the solution (Shelxt)[3] and refinement process (Shelxl 2018/3)[4] the data was validated by using Platon.[5] All non-hydrogen atoms were refined with anisotropic displacement parameters. All hydrogen atoms were placed at calculated positions and included in the refinement using a riding model. All graphic representations were created with Diamond 4.[6] Thermal ellipsoid plots were prepared with 50% of probability displacements for non-hydrogen atoms.

a. Crystal Data

Table S1: XRD-data of **1·MeCN, 1·MeOH** and **2**

	$[DMP]_2[C_5H_3(CO_2)_2H]$ ·MeCN (**1a·MeCN**)	$[DMP]_2[C_5H_3(CO_2)_2H]$ ·MeOH (**1a·MeOH**)	$[DMP]_2[C_5H_3(COS)_2H]$ (**2a**)
CCDC code	2055719	2055720	2055723
Identification code	**1a·MeCN**	**1a·MeOH**	**2a**
Empirical formula	C21 H35 N3 O4	C20 H36 N2 O5	C19 H32 N2 O2 S2
Formula weight	393.52	384.51	384.58
Temperature	100(2) K	100(2) K	100(2) K
Wavelength	1.54178 Å	1.54178 Å	0.71073 Å
Crystal system	Triclinic	Monoclinic	Monoclinic
Space group	P -1	P 21/c	P 21/c
Unit cell dimensions	a = 8.034(6) Å	a = 8.27130(10) Å	a = 8.921(2) Å
	b = 10.880(7) Å	b = 9.2215(2) Å	b = 13.110(3) Å
	c = 13.077(8) Å	c = 27.2279(4) Å	c = 34.179(7) Å
	α = 71.73(5)°.	α = 90°.	α = 90°.
	β = 76.41(6)°.	β = 94.2490(10)°.	β = 93.78(3)°.
	γ = 85.07(6)°.	γ = 90°.	γ = 90°.
Volume	1055.0(13) $Å^3$	2071.07(6) $Å^3$	3988.5(15) $Å^3$
Z	2	4	8
Density (calculated)	1.239 Mg/m^3	1.233 Mg/m^3	1.281 Mg/m^3
Absorption coefficient	0.691 mm^{-1}	0.712 mm^{-1}	0.282 mm^{-1}
F(000)	428	840	1664
Crystal size	0.282 x 0.179 x 0.116 mm^3	0.140 x 0.125 x 0.113 mm^3	0.473 x 0.232 x 0.195 mm^3
Theta range for data collection	3.649 to 66.499°.	3.255 to 66.498°.	2.288 to 25.023°.
Index ranges	-9<=h<=9, -12<=k<=10, -15<=l<=8	-9<=h<=9, -10<=k<=10, -32<=l<=15	-10<=h<=9, -15<=k<=15, -40<=l<=40
Reflections collected	18324	18491	56329
Independent reflections	3700 [R(int) = 0.0297]	3630 [R(int) = 0.0236]	7047 [R(int) = 0.0438]
Completeness to theta = x	99.5 % (x=64.499°)	99.6 % (x= 66.498°)	99.9 % (x= 25.023°)
Absorption correction	Semi-empirical from equivalents	Semi-empirical from equivalents	Semi-empirical from equivalents

Max. and min. transmission	0.9033 and 0.3555	0.9000 and 0.2861	0.95 and 0.89
Refinement method	Full-matrix least-squares on F^2	Full-matrix least-squares on F^2	Full-matrix least-squares on F^2
Data / restraints / parameters	3700 / 0 / 263	3630 / 0 / 258	7047 / 363 / 593
Goodness-of-fit on F2	1.026	1.042	1.061
Final R indices [I>2sigma(I)]	R1 = 0.0446, wR2 = 0.1300	R1 = 0.0648, wR2 = 0.1824	R1 = 0.0375, wR2 = 0.0780
R indices (all data)	R1 = 0.0489, wR2 = 0.1335	R1 = 0.0693, wR2 = 0.1902	R1 = 0.0502, wR2 = 0.0826
Extinction coefficient	0.0050(10)	0.0152(15)	-
Largest diff. peak and hole	0.325 and -0.245 e.Å^{-3}	0.423 and -0.304 e.Å^{-3}	0.280 and -0.267 e.Å^{-3}

Table S2: XRD-data of **3a, 7** and **9**

	[DMP]$_2$[η^5-C$_5$H$_4$(CO$_2$)Mo(CO)$_3$] · 0.5 MeCN (**3a**)	[η^6-p-cymene-Ru-η^5-C$_5$H$_3$(CO$_2$)$_2$H] (**7**)	[DMP]$_5$[La(κ^2-O$_2$C-C$_5$H$_4$)$_4$] (**9**)
CCDC code	2055722	2055721	2055724
Identification code	**3a**	**7**	**9**
Empirical formula	C44 H67 Mo2 N5 O10	C17 H18 O4 Ru	C54 H86 La N5 O8
Formula weight	1017.90	387.38	1072.18
Temperature	100(2) K	100(2) K	100(2) K
Wavelength	1.54186 Å	0.71073 Å	0.71073 Å
Crystal system	Monoclinic	Monoclinic	Monoclinic
Space group	P 2/n	P 21	P 21/c
Unit cell dimensions	a = 11.3848(2) Å	a = 7.976(2) Å	a = 15.4868(17) Å
	b = 9.73320(10) Å	b = 13.652(3) Å	b = 21.129(2) Å
	c = 20.9591(3) Å	c = 13.773(3) Å	c = 15.7368(16) Å
	α = 90°.	α = 90°.	α = 90°.
	β = 98.5640(10)°.	β = 91.35(3)°.	β = 90.179(3)°.
	γ = 90°.	γ = 90°.	γ = 90°.
Volume	2296.59(6) Å^3	1499.4(6) Å^3	5173.2(9) Å^3
Z	2	4	4
Density (calculated)	1.472 Mg/m^3	1.716 Mg/m^3	1.383 Mg/m3
Absorption coefficient	4.973 mm^{-1}	1.061 mm^{-1}	0.884 mm^{-1}
F(000)	1060	784	2264
Crystal size	0.146 x 0.144 x 0.141 mm^3	0.655 x 0.181 x 0.108 mm^3	0.188 x 0.162 x 0.146 mm^3
Theta range for data collection	4.266 to 75.858°.	2.101 to 24.956°.	2.322 to 28.320°.
Index ranges	-14<=h<=14, -11<=k<=12, -26<=l<=20	-9<=h<=9, -16<=k<=16, -15<=l<=16	-16<=h<=20, -27<=k<=27, -20<=l<=20
Reflections collected	51602	32761	110419
Independent reflections	5231 [R(int) = 0.0279]	5231 [R(int) = 0.0576]	12407 [R(int) = 0.0525]
Completeness to theta = x	100 % (x= 67.686°)	99.5 % (x= 24.956°)	99.8 % (x= 25.242°)
Absorption correction	Semi-empirical from equivalents	Semi-empirical from equivalents	Numerical Mu From Formula
Max. and min. transmission	0.5334 and 0.1872	0.7451 and 0.5715	1.0000 and 0.8291
Refinement method	Full-matrix least-squares on F^2	Full-matrix least-squares on F^2	Full-matrix least-squares on F2
Data / restraints / parameters	4755 / 0 / 282	5231 / 3 / 315	12407 / 269 / 690
Goodness-of-fit on F2	1.003	1.069	1.027
Final R indices [I>2sigma(I)]	R1 = 0.0212, wR2 = 0.0572	R1 = 0.0245, wR2 = 0.0590	R1 = 0.0437, wR2 = 0.1002
R indices (all data)	R1 = 0.0265, wR2 = 0.0588	R1 = 0.0263, wR2 = 0.0597	R1 = 0.0855, wR2 = 0.1295

Extinction coefficient	-	-	-
Largest diff. peak and hole	0.320 and -0.319 e.Å^{-3}	0.460 and -0.682 e.Å^{-3}	1.921 and -1.734 e.Å^{-3}

b. Molecular structures of the title compounds

[DMP]$_2$[C$_5$H$_3$(CO$_2$)$_2$H] · MeCN (1a · MeCN)

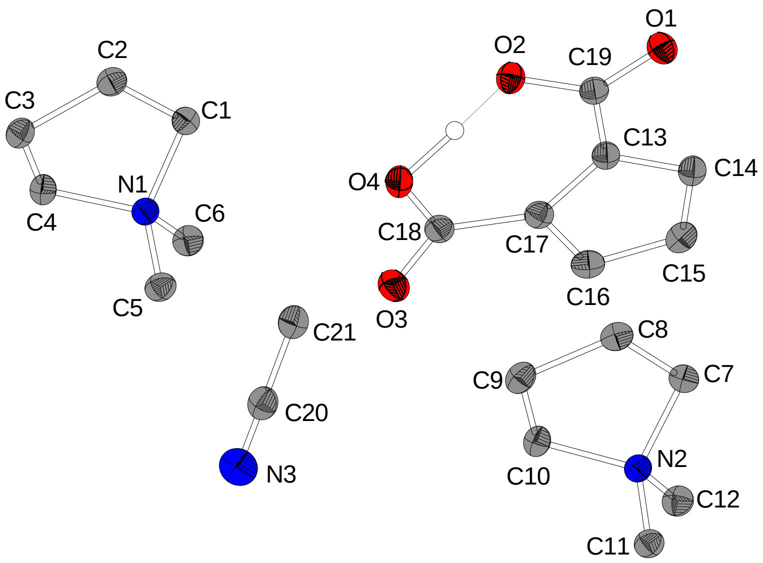

Figure S29. Reduced cell of the crystallographically determined molecular structure of [DMP]$_2$[C$_5$H$_3$(CO$_2$)$_2$H] · MeCN (**1 · MeCN**). Protons are not shown. Selected bond lengths (in Å) and bond angles (in °): O4-H1 1.2017(238), O2-H1 1.2308(241), O1-C19 1.2467(18), O2-C19 1.3088(19), O3-C18 1.2440(18), O4-C18 1.3142(19), C13-C19 1.469(2), C17-C18 1.468(2), O3-C18-O4 119.95(13), O3-C18-C17 121.17(14), O4-C18-C17 118.88(13), O1-C19-O2 120.26(13), O1-C19-C13 121.23(13), O2-C19-C13 118.50(13).

[DMP]$_2$[C$_5$H$_3$(CO$_2$)$_2$H] · MeOH (1a · MeOH)

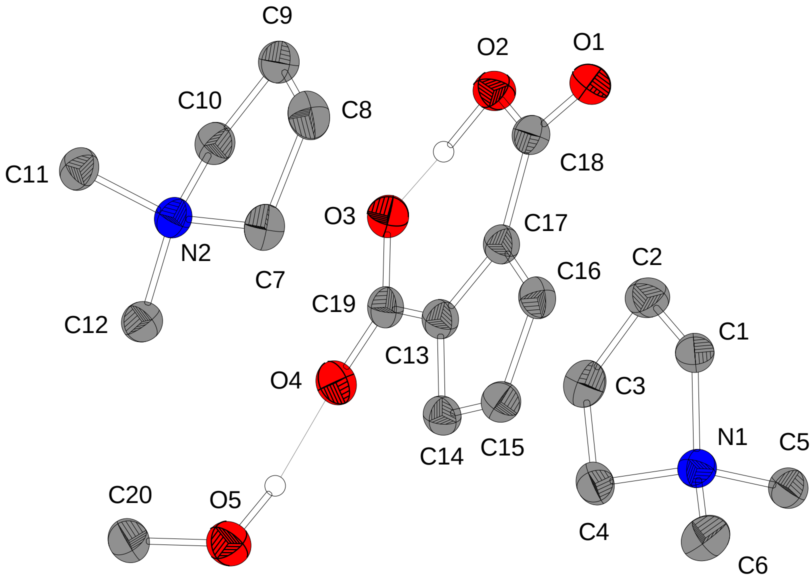

Figure S30. Reduced cell of the crystallographically determined molecular structure of [DMP]$_2$[C$_5$H$_3$(CO$_2$)$_2$H] · MeOH (**1 · MeOH**). Protons are not shown. Selected bond lengths (in Å) and bond angles (in °): O1-C18 1.238(3), O2-C18 1.321(3), O2-H2 1.13(4), O3-C19 1.308(3), O3-H2 1.31(4), O4-C19 1.254(3), O5-C20 1.423(3), O5-H5 1.03(4), C13-C14 1.414(3), C13-C17 1.439(3), C13-C19 1.464(3),

C14-C15 1.394(3), C15-C16 1.396(3), C16-C17 1.410(3), C17-C18 1.469(3), C18-O2-H2 108.7(18), C19-O3-H2 109.0(15), C20-O5-H5 110(2), C14-C13-C17 107.0(2), C14-C13-C19 122.4(2), C17-C13-C19 130.6(2), C15-C14-C13 108.9(2), C14-C15-C16 108.23(19), C15-C16-C17 109.1(2), C16-C17-C13 106.85(19), C16-C17-C18 122.5(2), C13-C17-C18 130.5(2), O1-C18-O2 119.5(2), O1-C18-C17 121.7(2), O2-C18-C17 118.8(2), O4-C19-O3 119.1(2), O4-C19-C13 121.6(2), O3-C19-C13 119.3(2).

[DMP]$_2$[C$_5$H$_3$(COS)$_2$H] (2a)

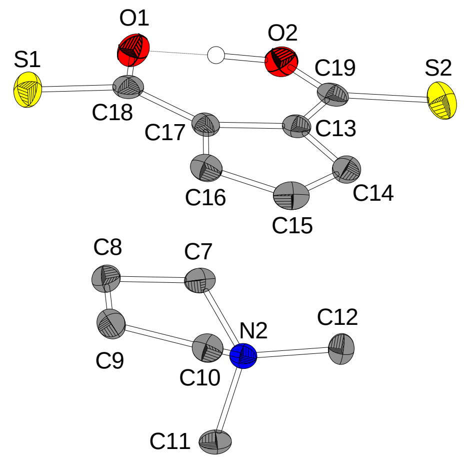

Figure S31. Reduced cell of the crystallographically determined molecular structure of [DMP]$_2$[C$_5$H$_3$(COS)$_2$H] (**2**). Protons are not shown. Selected bond lengths (in Å) and bond angles (in °): O1-H1 1.3713(262), O2-H1 1.2017(238), S1-C18 1.716(2), O1-C18 1.293(2), O1-H1 1.37(3), S2-C19 1.698(2), O2-C19 1.299(2), O2-H1 1.05(3), C19-C13 1.440(3), C18-C17 1.438(3), C18-O1-H1 111.6(12), C19-O2-H1 111.5(16), O2-C19-C13 119.78(17), O2-C19-S2 117.55(14), C13-C19-S2 122.66(15), O1-C18-C17 119.90(16), O1-C18-S1 118.12(14), C17-C18-S1 121.98(14).

[DMP]$_2$[C$_5$H$_4$(CO$_2$)Mo(CO)$_3$] (3a)

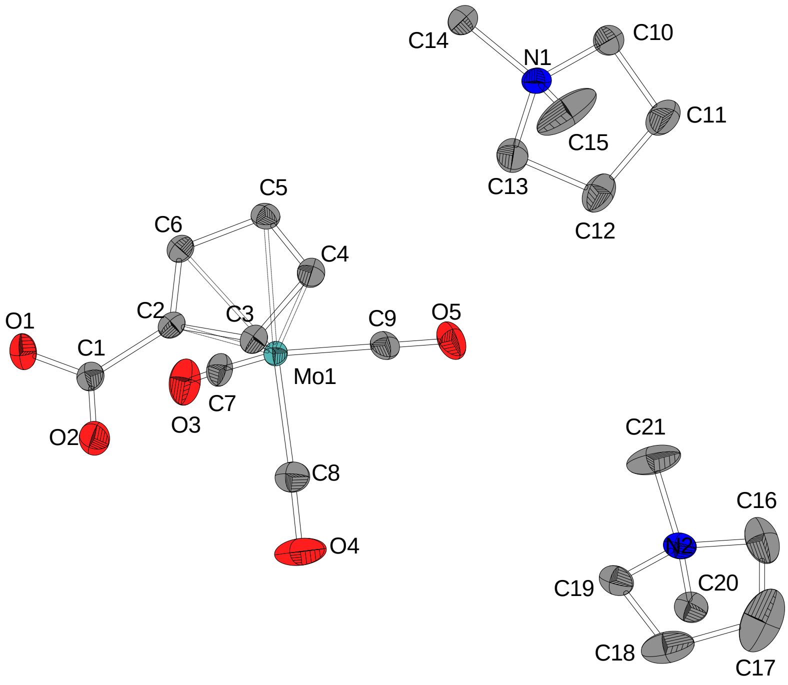

Figure S32. Molecular structure of [DMP]$_2$[C$_5$H$_4$(CO$_2$)Mo(CO)$_3$] (**3a**). Protons and disordered atoms are neglected for clarity. Ellipsoids are shown in 50% level. Selected bond lengths (Å) and angles (°) for a representative anion of **3a**: Mo1-C$_{Centroid}$ 2.0532(4), Mo1-C7 1.9304(17), O1-C1 1.2653(19), C1-O2 1.2551(19), C1-C2 1.504(2), C2-C6 1.430(2), C2-C3 1.4370(19), C3-C4 1.412(2), O3-C7 1.171(2),

C4-C5 1.434(2), C5-C6 1.417(2); C8-Mo1-C9 86.86(7), O2-C1-O1 125.67(15), O2-C1-C2 117.35(14), O1-C1-C2 116.93(13), C6-C2-C3 106.61(14), C6-C2-C1 126.93(13), C3-C2-C1 126.27(13), C4-C3-C2 108.80(13), C3-C4-C5 107.96(13), C6-C5-C4 107.65(13), C5-C6-C2 108.98(13).

[η^6-p-cymene-Ru-η^5-$C_5H_3(CO_2)_2H$] (7)

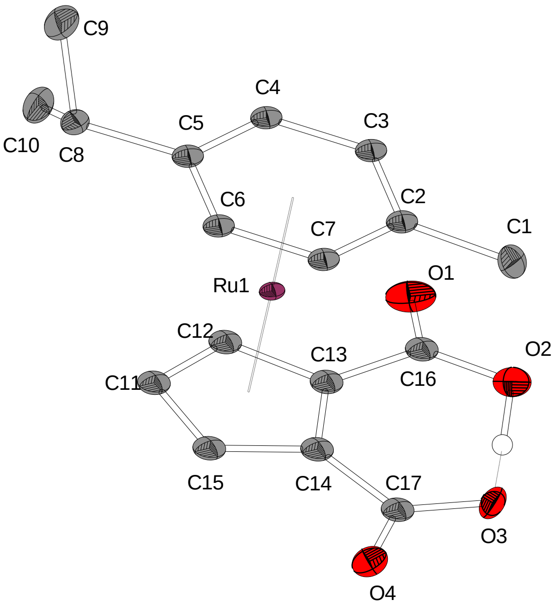

Figure S33. Reduced cell of the crystallographically determined molecular structure of [η^6-p-cymene-Ru-η^5-$C_5H_3(CO_2)_2H$] (**7**). Protons are not shown. Selected bond lengths (in Å) and bond angles (in °): O2-H2 1.1324(463), O3-H2 1.3008(453), Ru1-$C_{centroid}$ (Cp) 1.8178(6), Ru1-$C_{centroid}$ (p-cymene) 1.6994(6), O1-C16 1.206(7), C1-C2 1.505(7), O2-C16 1.305(6), O2-H2 1.14(3), C2-C3 1.413(8), C2-C7 1.432(8), C13-C16 1.519(7), C17-C14 1.501(7), O4-C17-O3 125.5(5), O4-C17-C14 118.5(5), O3-C17-C14 116.0(5), O1-C16-O2 124.3(5), O1-C16-C13 119.3(5), O2-C16-C13 116.4(4).

$[DMP]_5[La(C_5H_4CO_2)_4]$ (9)

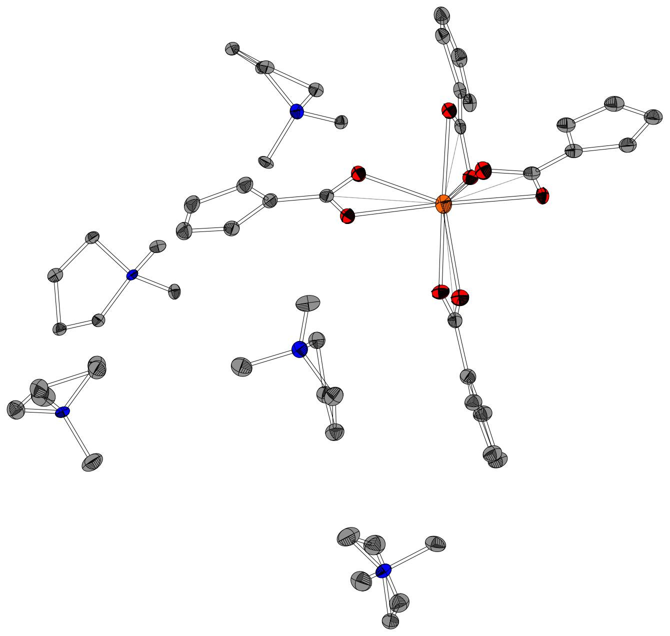

Figure S34. Reduced cell of the crystallographically determined molecular structure of $[DMP]_5[La(\kappa^2\text{-}O_2C\text{-}C_5H_4)_4]$ (**9**). Protons are not shown. Selected bond lengths (in Å) and bond angles (in °): La1-O1 2.507(6), La1-O2 2.562(5), La1-C6 2.920(9), O1-C6 1.292(11), O2-C6 1.284(10), C5-C6 1.448(12), O1-La1-O8 126.6(2),O1-La1-O5 82.06(19), O8-La1-O5 123.4(2), O8-La1-O3 134.22(18), O5-La1-O3 91.2(2), O8-La1-O7 52.08(19), O3-La1-O7 172.70(18), C6-O1-La1 95.0(5), C6-O2-La1 92.7(5), O2-C6-O1 119.6(8)

4. Thermogravimetric measurement of $[NMe_4]_2[(C_5H_3(CO_2)_2H]$ (1b)

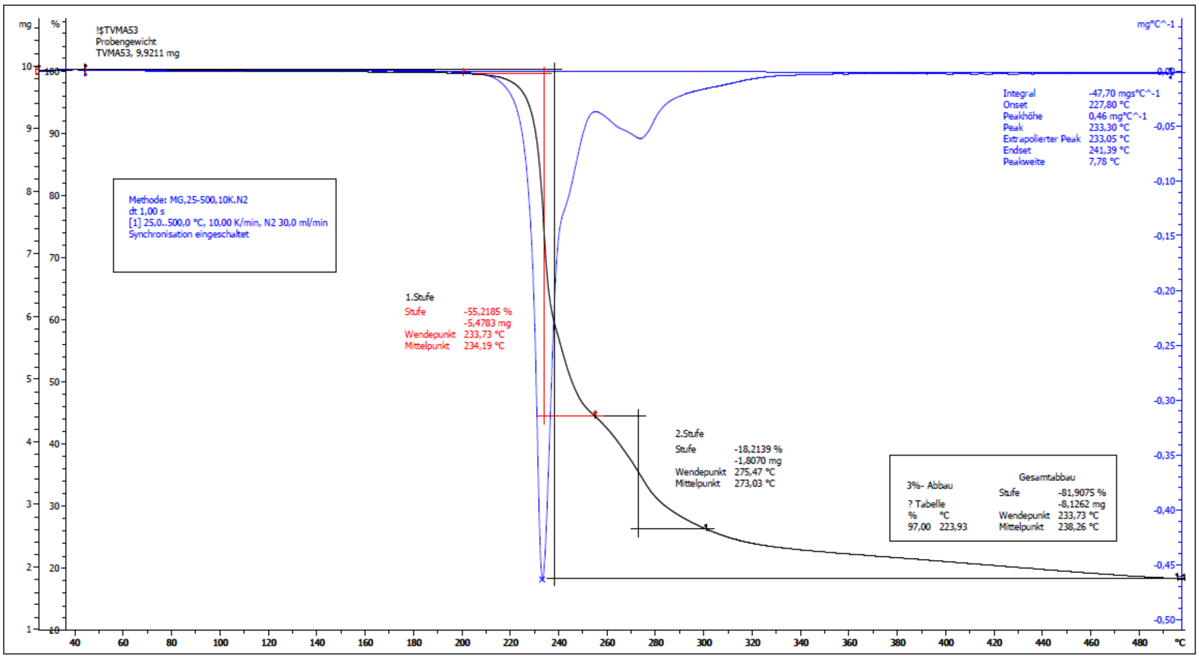

Figure S35. TGA-DSC of **1b** (heating rate 10 K min^{-1}; 25 °C - 500 °C, then 30 min at 500 °C).

5. Acidic and thermal decarboxylation study of 1a, 3 and 5

Samples of 10 mg of **1a**, **3** and **5** were dissolved in DMSO-d_6 in a tightly sealed NMR tube and thermally treated as described in the figures. The signals of the cation have been hidden for clarity, since they are unchanged.

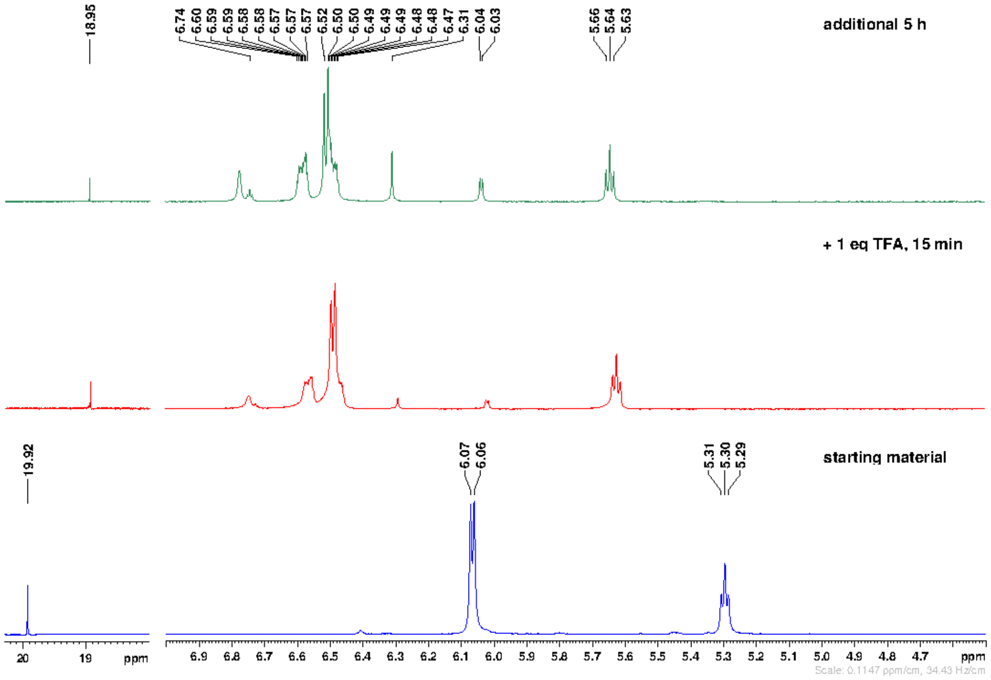

Figure S36. ^1H NMR spectra after addition of TFA to $[DMP]_2[(C_5H_3(CO_2)_2H]$ (**1a**) lead to mixture of not identified decomposition products (DMSO-d_6, 25 °C, 300.1 MHz).

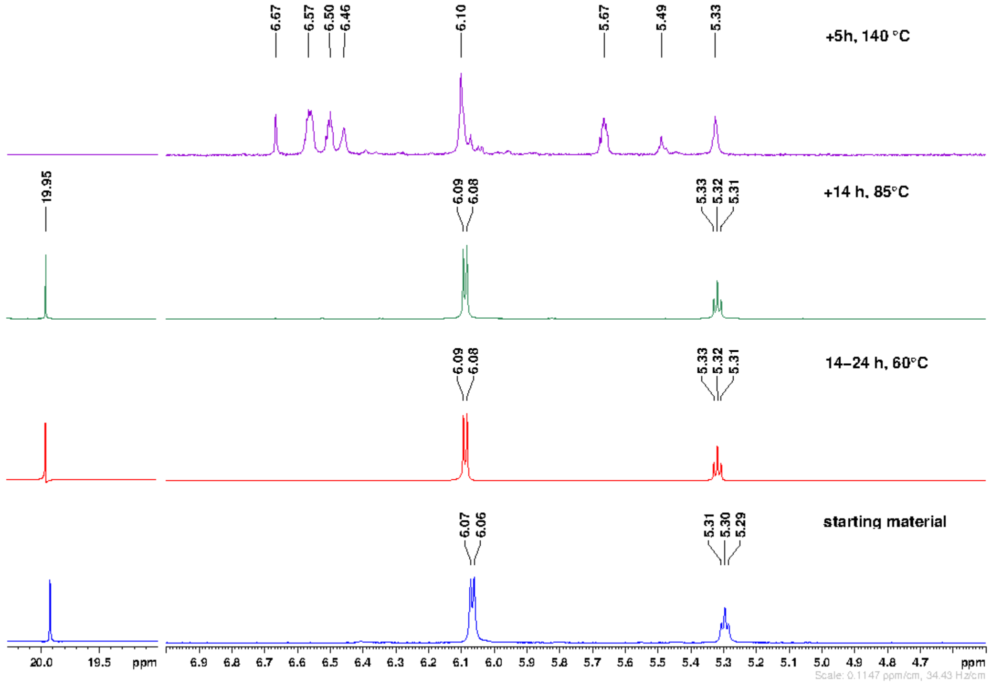

Figure S37. ^1H NMR spectrum of $[DMP]_2[(C_5H_3(CO_2)_2H]$ (**1a**) after heading to 140 °C for 5 h leading to an almost complete decomposition of the Cp backbone (DMSO-d_6, 25 °C, 300.1 MHz).

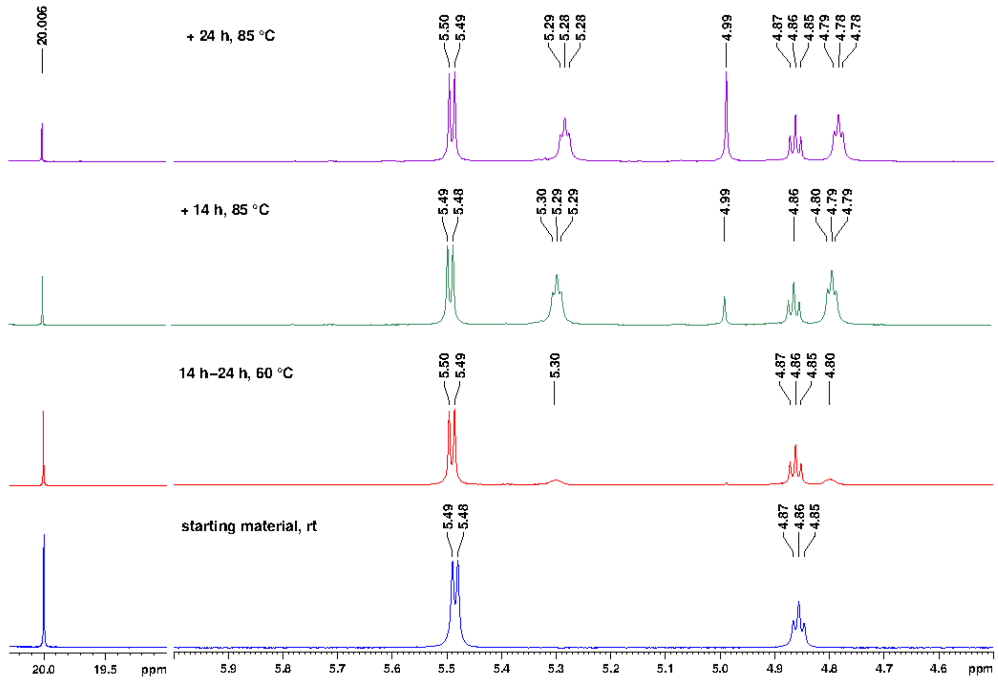

Figure S38. ^{1}H NMR spectrum of $[DMP]_2[(\eta^5\text{-}C_5H_3(CO_2)_2H)Mo(CO)_3]$ (**3**) after heading it to 60-85 °C leading to a selective first decarboxylation product **3a** in equilibrium with **3**. (DMSO-d_6, 25 °C, 300.1 MHz).

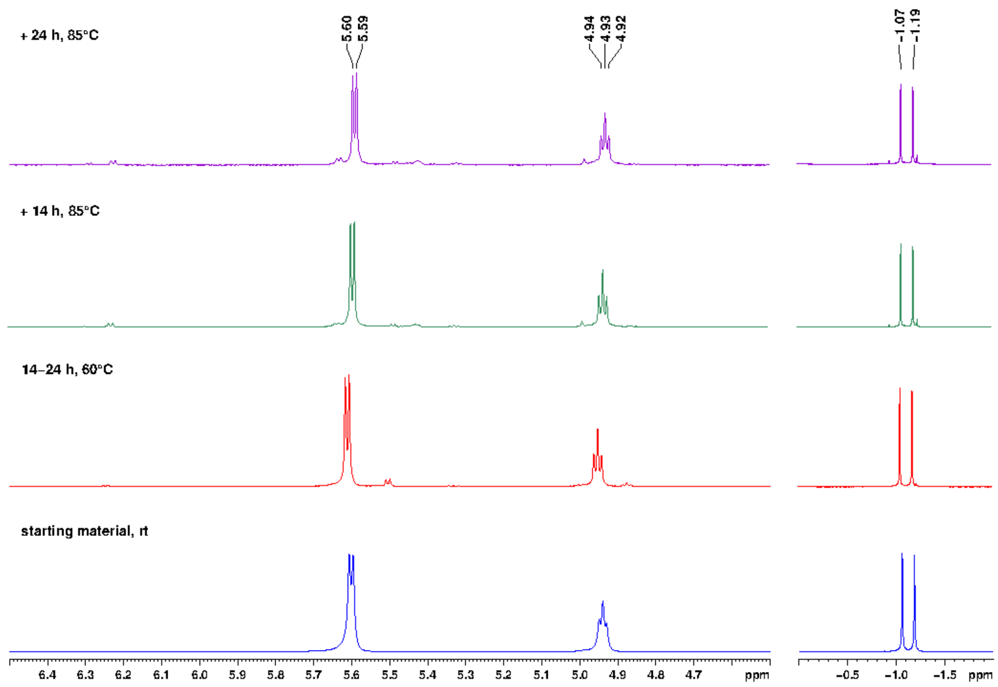

Figure S39. ^{1}H NMR spectrum of $[DMP]_2[(\eta^5\text{-}C_5H_3(CO_2)_2AlMe_2)Mo(CO)_3]$ (**5**) after heading it to 60-85 °C does not allow a kinetically favored decarboxylation path at these temperatures (DMSO-d_6, 25 °C, 300.1 MHz).

6. References

(1) W. L. F. Armarego, D. D. Perrin, *Purification of laboratory chemicals*, 4th Ed., Butterworth-Heinemann, Oxford, **1997**.

(2) S. Harder, M. H. Prosenc, U. Rief, *Organometallics* **1996**, *15*, 118.

(3) Sheldrick, G. M. Crystal structure refinement with SHELXL. *Acta Cryst. C, Structural Chemistry* **2015**, *71*, 3–8, DOI: 10.1107/S2053229614024218.

(4) Hübschle, C. B.; Sheldrick, G. M.; Dittrich, B. ShelXle: A Qt graphical user interface for SHELXL. *J. Appl. Cryst.* **2011**, *44*, 1281–1284, DOI: 10.1107/S0021889811043202.

(5) Spek, A. L. Structure validation in chemical crystallography. *Acta Cryst. D, Biological Crystallography* **2009**, *65*, 148–155, DOI: 10.1107/S090744490804362X.

(6) K. Brandenburg, H. Putz. Diamond; *Crystal Impact GbR:* Bonn, **2012**.
